# Impacts of systemic treatments on health-related quality of life for patients with metastatic colorectal cancer: a systematic review and network meta-analysis

**DOI:** 10.1186/s12885-024-11937-z

**Published:** 2024-02-09

**Authors:** Yunlin Jiang, Mingye Zhao, Wenxi Tang, Xueping Zheng

**Affiliations:** 1 Nanjing Hospital of Chinese Medicine Affiliated to Nanjing University of Chinese Medicine, Nanjing, China; 2 Nanjing University of Chinese Medicine, Nanjing, China; 3 Center for Pharmacoeconomics and Outcomes Research & Department of Public Affairs Management, School of International Pharmaceutical Business, China Pharmaceutical University, Nanjing, Jiangsu China

**Keywords:** Metastatic colorectal cancer, Health-related quality of life, Network meta-analysis, Systematic review, Treatment selection

## Abstract

**Objective:**

There is limited evidence of comparative results among different treatments regarding impacts of Health-Related Quality of Life (HRQoL) for patients with metastatic colorectal cancer (mCRC). We aimed to compare efficacy of systemic treatments on HRQoL among patients with mCRC.

**Methods:**

We collected randomized controlled trials (RCTs) reported in English up until July 2023, from databases including PubMed, Embase, Cochrane Library, ClinicalTrials.gov, and prominent conference databases, for this Bayesian network meta-analysis. Phase 2 or 3 trials that evaluated at least two therapeutic regimens were included. Primary outcomes were short-term and long-term mean changes in EORTC QLQ-C30 global health status/quality of life (GHS/QoL) scores. Secondary outcome was mean change in EQ-5D health utility scores. Mean differences (MDs) with 95% confidence intervals (CIs) were used as effect size. Subgroup analysis was performed based on whether patients received systemic treatments before. We conducted various sensitivity analyses, including differentiating between chemotherapy types, and analyzed patient cohorts with non-specified gene expression levels as well as those with target KRAS expression statuses. The current systematic review protocol was registered on PROSPERO (CRD42023453315 and CRD42023420498).

**Results:**

Immunotherapy and targeted therapy significantly improved HRQoL over chemotherapy, with MDs of 9.27 (95% CI: 3.96 to 14.6) and 4.04 (95% CI: 0.11 to 7.94), respectively. Monotherapy significantly outperformed both combination therapy (MD 5.71, 95%CI 0.78 to 10.63) and no active treatment (MD 3.7, 95%CI 1.41 to 6.01) regarding GHS/QoL in the short-term. Combining targeted therapy with chemotherapy did not improve HRQoL. Focusing on HRQoL, cetuximab excelled when gene expression baselines were unspecified. Subgroup and sensitivity analyses upheld these robust findings, unaffected by model or patient baseline characteristics. Evidence from clinical trials without specific gene level data suggested that monotherapies, especially targeted therapies such as cetuximab, demonstrated superiority in HRQoL. For KRAS wild-type patients, no significant HRQoL differences emerged between chemotherapy, targeted therapy, or their combination..

**Conclusions:**

Targeted therapies and immunotherapy demonstrate superior HRQoL benefits, monotherapy such as cetuximab is associated with significant improvements as compared to combination therapy. However, tailoring these results to individual gene expression profiles requires more evidence.

**Supplementary Information:**

The online version contains supplementary material available at 10.1186/s12885-024-11937-z.

## Highlights

It is the first to systematically compare the impacts of different drug mechanisms, drug quantities, and specific treatments on the HRQoL of mCRC patients. The findings of our investigation are as follows:

• Monotherapy was associated with great improvements in patient HRQoL compared with combination therapy and NAT.

• Immunotherapy and targeted therapy exhibited the most favorable outcomes regarding HRQoL. Incorporating targeted therapy with chemotherapy did not enhance patient performance in terms of HRQoL.

• In managing mCRC, cetuximab was particularly effective in enhancing HRQoL when gene expression baselines were unspecified, especially for those previously received systematic treatment. Considering HRQoL, efficacy, and safety collectively, cetuximab proved to be an advantageous treatment for patients with wild-type KRAS mutations.

**Supplementary Information:**

The online version contains supplementary material available at 10.1186/s12885-024-11937-z.

## Introduction

Colorectal cancer (CRC) ranks as the third most prevalent cancer globally and is responsible for the third highest number of cancer-induced fatalities [1]. It is estimated that more than 1.9 million new cases of CRC occurred in 2020, resulting in 935,000 deaths [2]. Metastatic CRC (mCRC) is observed in approximately 25% of all patients, while roughly 50% of patients without metastases will eventually develop metastasis [3]. In recent years, advancements in targeted therapies have led to an increase in the lifespan of patients with mCRC. Over the 10-year course, the 3-year survival rate for patients with metastatic rectal cancer has increased from 25 to 30% [1].

The fact that cancer and its treatment impact not only patients' health but also their Health-Related Quality of Life (HRQoL) is widely acknowledged [4, 5]. HRQoL has been extensively created and employed as an indicator of patients' reported outcomes [6]. HRQoL assessment instruments typically gauge five dimensions of quality of life (QoL), namely physical, role, cognitive, emotional, and social functioning. The evaluation of HRQoL carries numerous potential applications and implications for both clinical practice and research, making it a significant outcome in clinical trials. Generally, HRQoL can be measured using various scales, and the two most commonly used are the following: European Organization for Research and Treatment of Cancer general health status and quality-of-life questionnaire (EORTC QLQ-C30), and EuroQoL-five dimension index questionnaire (EQ-5D) [7]. EORTC QLQ-C30 consists of 15 dimensions, among which global health status/quality of life (GHS/QoL), composed of two items, is commonly used to reflect the overall level of patient's HRQoL [8]. The GHS/QoL score is based on a seven-point Likert scale that spans from 'very poor' to 'excellent'. EQ-5D questionnaire is composed of two components, namely the visual analogue scale (VAS) and health utility scores (HUS) [9], and it is frequently used to evaluate health outcomes using a descriptive system comprising five dimensions, which include mobility, self-care, usual activities, pain/discomfort, and anxiety/depression [10].

As survivorship rates and treatment options increase, ensuring a good HRQoL has become essential in conjunction with prolonging life [11]. Therefore, HRQoL is widely acknowledged as one of the primary endpoints for treatment evaluation [12]. However, studies about comparisons of HRQoL between different treatments for mCRC patients are quite limited. Thus, we conducted a network meta-analysis of randomized controlled trials (RCTs) to comprehensively compare the impacts of systemic treatments on HRQoL, and to provide references for healthcare clinicians, patients, and relevant guidelines in clinical medication and disease management.

## Methods

Our study was conducted in accordance with the guidelines of the Preferred Reporting Items for Systematic Reviews and Meta-Analyses (PRISMA) extensions for network meta-analysis (NMA) [13]. See Supplementary File 1. This systematic review protocol was registered on PROSPERO (CRD42023453315 and CRD42023420498).

### Data sources and search strategy

The search strategy is provided in Supplementary File 2. By July 31, 2023, we conducted a comprehensive search on PubMed, EMBASE, Cochrane Library, and ClinicalTrials.gov to find relative RCTs and published studies. There were no restrictions on the publication date, and we limited our review to studies published in English to ensure the precision of data interpretation and analysis, given the research team's proficiency.. Moreover, abstracts from the European Society for Medical Oncology and American Society of Clinical Oncology between 2020 to 2023 were also included in the search.

### Selection criteria

Two researchers initially screened the titles and abstracts of the included articles. The eligibility criteria based on the PICOS framework were as follows:

1. Population: Adult patients with confirmed advanced or metastatic unresectable CRC, diagnosed either histologically or cytologically. No limitations were imposed regarding individual-level characteristics.
2. Interventions and comparisons: Any systemic interventions, including pharmaceutical, surgical, radiological, and combination therapies, were evaluated.
3. Outcomes: Trials should be reported on at least one of the following outcomes: EORTC QLQ-C30 GHS/QoL score; EQ-5D VAS and HUS. We chose EORTC QLQ-C30, and EQ-5D as HRQoL measurements as they were the most frequently used in RCTs on mCRC [7]. Regarding the range of the GHS/QoL subscale from the EORTC QLQ-C30 questionnaire, it typically ranges from 0 to 100. A high value on the GHS/QoL scale is considered indicative of good global health status and higher quality of life, whereas a low score would suggest poorer health and quality of life issues. As for the minimal clinically important difference for the GHS/QoL, a difference of 5 to 10 points on the GHS/QoL scale is often considered as a minimal clinically significant change.
4. Study design: Phase 2 or 3 studies that compared multiple distinct treatments were primarily considered.

To avoid repetition, we only considered trials that provided the most recent and informative data. Furthermore, we disregarded trials that studied treatments not related to any comparisons. Additionally, trials that examined different dosages but with the same administrations were also excluded.

### Data extraction and quality assessment

Two independent researchers (YJ and MZ) were responsible for extracting the required data. Discrepancies were sorted out through discussions involving other researchers (YJ, MZ, WT, and XZ). The extracted information encompassed the characteristics of eligible trials (publication year, registration information, etc.), characteristics of populations (age, sample size, countries, etc.), and characteristics of the program (interventions, outcomes of endpoints, etc.). The clinical outcomes extracted included were mean changes from baseline in EORTC QLQ-C30 GHS/QoL scores and EQ-5D HUS. EQ-5D VAS scores were not finally considered for two reasons: Firstly, the validity of EQ-5D HUS is higher for cancer patients [14]; Secondly, the trails included in this study reported EQ-5D HUS more frequently.

Cochrane Collaboration's risk of bias (ROB) tool was used to evaluate the quality of the studies included [15]. The eligible studies were categorized into three groups: high, low, or unclear risk [16]. Publication bias was evaluated by Egger regression test was utilized, with p-values < 0.05 being interpreted as evidence of bias.

### Statistical analyses

The primary outcomes were short-term and long-term least squares mean changes in EORTC QLQ-C30 GHS/QoL scores. The short-term period was defined as 8–12 weeks from baseline, and the long-term was defined as the time from baseline to the endpoint. Secondary outcome encompassed the mean changes in EQ-5D HUS from baseline to endpoint. Mean difference (MD) with 95% confidence interval (CI) was utilized as the effect size. Data solely presented in figures, was extracted using image extraction software (WebPlotDigitizer). In the absence of standard deviation (SD), SDs were calculated using SEs, 95% CIs, or P-values assuming that the values followed a normal distribution [17]. When SDs of changes from baseline were missing, assuming a correlation coefficient of 0.5, we estimated them used baseline and final SDs, as suggested by the Cochrane Collaboration Handbook (https://handbook-5-1.cochrane.org/chapter_16). In our analysis, we considered best supportive care or placebo as no active treatment (NAT), chemotherapy regimens were not divided in overall analysis for statistical purposes.

Network plots were generated to compare and visualize the various treatment arms. The Bayesian approach was employed for the analysis of synthesized MDs. Considering that most of direct evidence was from one trial, the fixed-effects consistency model was employed [18]. The Bayesian NMA was conducted using the R statistical packages Gemtc and BUGSnet. Four sets of Markov chains were used, consisting of 50,000 samples each, with 10,000 burn-in samples. Non-informative uniform and normal prior distributions were employed [19]. Furthermore, we computed the probability ranking for every accessible treatment, illustrating it through the surface under the cumulative ranking (SUCRA). SUCRA values are interpreted as indicating the relative ranking of treatments. These values, which range from 0 to 100%, reflect the likelihood of each treatment being the most effective option within the network, with higher values suggesting better relative performance.

In our network meta-analysis, we adhered to the transitivity assumption, ensuring comparability across interventions. Heterogeneity among studies was evaluated utilizing the I^2^ statistic. A value exceeding 50% indicated a moderate level of heterogeneity [17]. Coherence between direct and indirect evidence was assessed using node-splitting methods, with discrepancies scrutinized for methodological or clinical reasons [17]. Several comparisons with pairwise meta-analysis were performed to verify the robustness of this study. Trace plots and Gelman-Rubin diagnostic statistics were employed to verify the convergence of Markov chains [20]. We utilized the CINeMA (Confidence in Network Meta-Analysis) framework to assess the certainty of evidence for this network-meta-analysis.

To evaluate the strength and dependability of the findings, we conducted subgroup and sensitivity analyses. Subgroup analyses were conducted to assess the influence of the number of treatment lines received (first-line or subsequent-line). In the sensitivity analyses, first, we made a distinction between chemotherapy treatments. Second, we reappraised the primary outcomes in the studies using a random-effects model to confirm the robustness of the fixed-effects model findings. Third, we excluded RCTs targeting on patients with BRAF/RAS mutated and microsatellite instability-high or mismatch repair deficient (MSI-H/dMMR). Fourth, we included data from clinical trials that analyzed patient cohorts with non-specified baseline gene expression profiles. Finally, we exclusively incorporated data from KRAS wild-type populations for targeted analysis within this group. Regrettably, among the final included articles, only one study each reported outcomes for populations with MSI-H, KRAS, or BRAF mutations [21–23], preventing us from conducting independent analyses for these subgroups. We did not differentiate standard chemotherapy regimens (see definition in Supplementary File 3) in order to establish connected networks. We believe it is justified due to the similarity in medication for these regimens. Furthermore, there is no difference in patients' HRQoL, as assessed by QLQ-C30 GHS/QoL, among these regimens [24, 25].

## Results

### Characteristics of the included studies

**Fig. 1:**
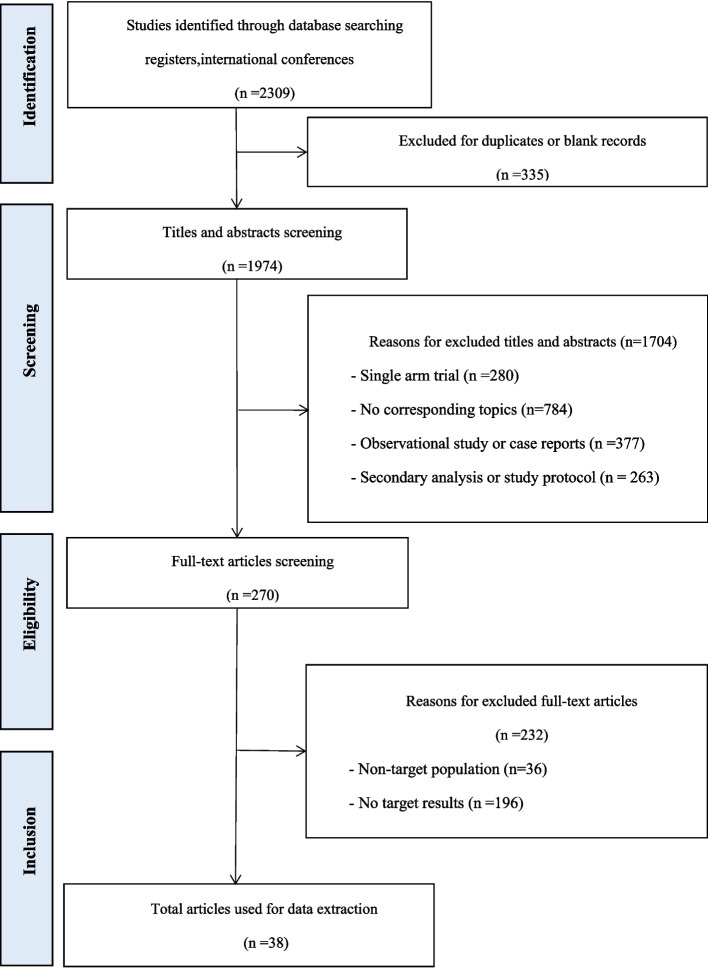
Study flow chart

Our study included 38 articles, comprising a total of 20 intervention schemes. Specificlly, A total of 2,309 records were identified from the aforementioned databases. Among them, 270 studies were found eligible for full-text review. Eventually, the analysis included 36 RCTs comprising 38 articles. Flow chart is presented in Fig. 1. Characteristics of included studies are provided in Table 1 and Supplementary File 4.

**Table 1:** Characteristics of the included studies

Trial	Study arms		Number of randomized patients		Age (Mean, range or SD)		Sex (Male,%)		Line	Outcomes
	I	C	I	C	I	C	I	C		
Antonio 2021 [26]	modified FOLFOX-6/modified CAPOX + bevacizumab administered 4 days before chemotherapy	modified FOLFOX-6/modified CAPOX + bevacizumab administered on the same day as chemotherapy	115	115	61(53–68)	63(56–68)	60	58.3	F	GHS (ST and LT)
ALTER0703 [27]	Anlotinib	Placebo	282	137	56.2 ± 10.5	55.2 ± 10.8	63	66	S	GHS (ST and LT)
Liu 2020 [28]	Traditional Chinese Medicine Combined With Chemotherapy and Cetuximab or Bevacizumab	Placebo Combined With Chemotherapy and Cetuximab or Bevacizumab	160	160	63.89 ± 10.11	62.19 ± 7.84	54	69	F + S	GHS (LT)
CONCUR [29, 30]	Regorafenib	placebo	136	68	58.0 (31.0–79.0)	55.0 (33.0–84.0)	66	45	S	GHS(LT) HUS
Xu 2017 [31]	Famitinib	placebo	99	55	55 (24–70)	54 (32–71)	57	60	S	GHS (ST and LT)
BEACON CRC [21]	I1:Encorafenib + cetuximab with binimetinib;I2:Encorafenib + cetuximab;I3:Irinotecan or FOLFIRI + cetuximab		224/220/221		62 (26–85)/61(30–91)/60(27–91)		47/52/43		S	GHS (ST and LT)
PRODIGE 18 [32]	Bevacizumab + chemotherapy	Cetuximab + chemotherapy	65	67	61 (33–83)	63(37–84)	63	66	S	GHS (ST and LT)
NORDIC9 [33]	reduced-dose S1 + oxaliplatin	S1	71	79	77.8 ± 13.7	78.4 ± 4.0	49	51	F	GHS (ST and LT)
KEYNOTE-177 [22]	pembrolizumab	mFOLFOX6 or FOLFIRI	153	154	63.0(24–93)	62.5(26–90)	46	53	F	GHS (ST and LT) HUS
Valentino [34]	Panitumumab + 5-FU/LV	Panitumumab	107	103	64 (54–69)	63 (56–70)	66	65	F	GHS(ST and LT)
Jane 2020 [35]	FOLFOX + SIRT	FOLFOX	NA	NA	NA	NA	NA	NA	F	GHS (ST and LT)
Filippo 2020 [36]	Capecitabine + temozolomide	FOLFIRI	43	43	70.0(63.0–74.5)	67.0(61.0–73.0)	42	56	S	GHS (ST and LT)
REVERCE [37]	Regorafenib	cetuximab	51	50	68(62–74)	65(57–70)	61	66	S	HUS
NORDIC-VII [38]	I1:FLOX/I2: Cetuximab + FLOX/C:Cetuximab and intermittent FLOX		172/171/169		60.1(29.9–74.8)/ 59.5(26.9–74.4)/62.2(35.7–74.9)		55/61/62		F	GHS (ST and LT)
CRYSTAL Subgroup 1 [39]	Cetuximab + FOLFIRI	FOLFIRI	170	181	60(24–79)	59(19–82)	64	66	F	GHS (ST and LT)
AIO KRK 0207 [40]	I1:fluoropyrimidine(FP) + BEV/I2:BEV/C:No treatment		136/142/135		64(25–82)/65(32–82)/66(32–82)		67/68/63		S	GHS (ST and LT)
CRYSTAL Subgroup 2 [41]	Cetuximab + FOLFIRI	FOLFIRI	300	327	60(24–80)	59(19–84))	63	62	F	GHS (ST and LT)
Yong 2013 [42]	Capecitabine	Capecitabine + oxaliplatin (CAPOX)	40	40	71.0 (66–81)	72.0 (65–79)	58	55	F	GHS (ST and LT)
PRIME 1 [43]	Panitumumab + FOLFOX4	FOLFOX4	284	292	60.5(10.5)	60.1(11.3)	67	64	F	HUS
PRIME 2 [43]	Panitumumab + FOLFIRI	FOLFIRI	263	267	60.1(10.1)	60.6(10.1)	61	64	S	HUS
DaVINCI [44]	Irinotecan	FOLFIRI	44	44	66(26–84)	64(35–78)	59	70	S	GHS (LT)
Dawn 2010 [45]	Panitumumab + BSC	BSC	112	96	62 (10)	62 (10)	71	65	S	HUS
CO.17 Trial [46]	Petuximab + BSC	BSC	287	285	NA	NA	NA	NA	S	GHS (ST and LT)
Jolien 2009 [47]	Chemotherapy + bevacizumab	Chemotherapy + bevacizumab + cetuximab	368	368	62(27–83)	62(33–80)	56	63	F	GHS (LT)
Southern Italy Cooperative Oncology study 0401 [48]	OXAFAFU	OXXEL	164	158	65 (37–79)	64 (39–84)	54	66	F	GHS (ST and LT)
EPIC [49]	Cetuximab + irinotecan	Irinotecan	648	650	61(23–85)	62(21–90)	63	63	S	GHS (ST and LT)
Rohit 2004 [50]	8 cycle irinotecan	Continue Irinotecan	30	25	65(42–76)	64(45–78)	67	64	S	GHS (ST and LT)
EORTCG Group Study 40,952 [51]	FU24h/FU24h + LV/FU + LV		166/164/167		61(25–76)/62(23–76)/61(32–76)		54/62/63		F	GHS (ST and LT)
Charles 2003 [52]	Weekly Irinotecan	Every 3-Week Irinotecan	95	196	NA	NA	62	58	S	GHS (ST and LT)
Saltz 2000 [53]	Irinotecan + fluorouracil + leucovorin	Fluorouracil and leucovorin	231	226	62(25–85)	61(19–85)	65	54	F	GHS (ST and LT)
Hill 1995 [54]	Fluorouracil + Interferon Alfa-2b	Fluorouracil	77	78	57(16–79)	61(36–78)	68	54	F	GHS (ST and LT)
ASPECCT [55]	Panitumumab	Cetuximab	499	500	61.0 (54–67)	60.5 (53–68)	63	64	S	HUS
Peeters 2014 [56]	Panitumumab + FOLFIRI	Panitumumab	303	294	60 (28–84)	61 (29–86)	62	65	S	HUS
PICCOLO [57]	Panitumumab + irinotecan	irinotecan	230	230	64 (57–70)	63 (56–69)	70	69	S	GHS (LT)
LUME-Colon 1 [58]	Nintedanib	Placebo	386	382	62 (22–85)	62 (23–83)	61	57	S	GHS (LT)
CORRECT [59]	Regorafenib	placebo	505	255	61 (54·0–67·0)	61 (54·0–68·0)	62	60	S	GHS (LT) and HUS
Yong 2012 [60]	S-1 + oxaliplatin	CAPOX	168	172	61 (53–66)	60 (52–66)	65	59	F	GHS (LT)
PanaMa [61]	Panitumumab + FU/FA	FU/FA	125	123	66 (44–84)	65 (30–86)	70	63	F	GHS (ST and LT)

*F* First-line, *FA* Folinic acid, *FU* Fluorouracil, *GHS* QLQ-C30 global health status, *HUS* EQ-5D health utility score, *LT* long-term, *LV* leucovorin, *S* second-line, *ST* short-term. More information about medication, please see Supplementary File 3

A total of 18,385 patients diagnosed with mCRC were included in this research study. Among them, 18 RCTs examined first-line treatments, while the others evaluated subsequent-line treatments for previous treated patients. Briefly, 20 treatments were involved, comprising of chemotherapy, anlotinib, bevacizumab, bevacizumab plus chemotherapy, cetuximab, cetuximab plus chemotherapy, cetuximab plus intermittent chemotherapy, cetuximab combined with bevacizumab plus chemotherapy, encorafenib plus cetuximab, encorafenib combined with cetuximab plus binimetinib, famitinib, interferon plus chemotherapy, nintedanib, panitumumab, panitumumab plus chemotherapy, pembrolizumab, regorafenib, SIRT plus chemotherapy, traditional Chinese medicine combined with bevacizumab plus chemotherapy, and NAT. Detailed regimen information has been presented in Supplementary File 3.

### Risk of bias

The assessment of ROB is presented in Supplementary File 5. Overall, ROB in all RCTs was generally low. However, multiple RCTs were open-label [21, 22, 26, 32, 34, 36, 37, 39, 41–43, 47, 49, 52, 55–57, 60, 61], thereby raising concerns about participant and personnel blinding, outcome assessment, and allocation concealment. Additionally, there have been concerns regarding potential bias in several RCTs due to inadequate outcome data availability [28, 38, 42, 57]. The results of the Egger test indicated no publication bias in our network, the funnel plots are displayed in Supplementary File 6.

### Efficacy outcomes

#### Overall analysis

**Fig. 2:**
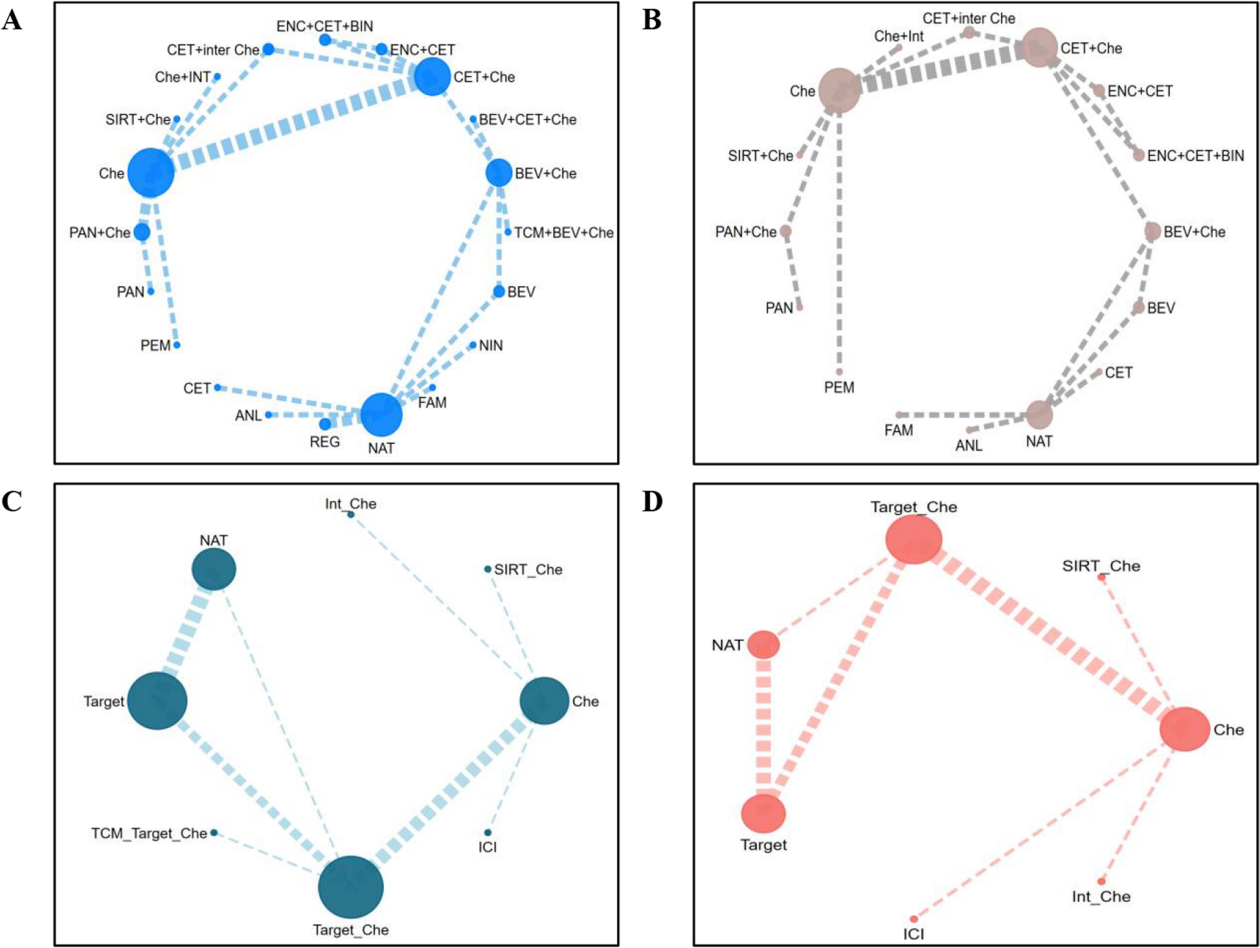
Network Plot. **A** Overall analysis of long-term QLQ-C30 for all patients; **B** Overall analysis of short-term QLQ-C30 for all patients; **C** Overall analysis of long-term mechanism comparisons; **D** Overall analysis of short-term mechanism comparisons

**Fig. 3:**
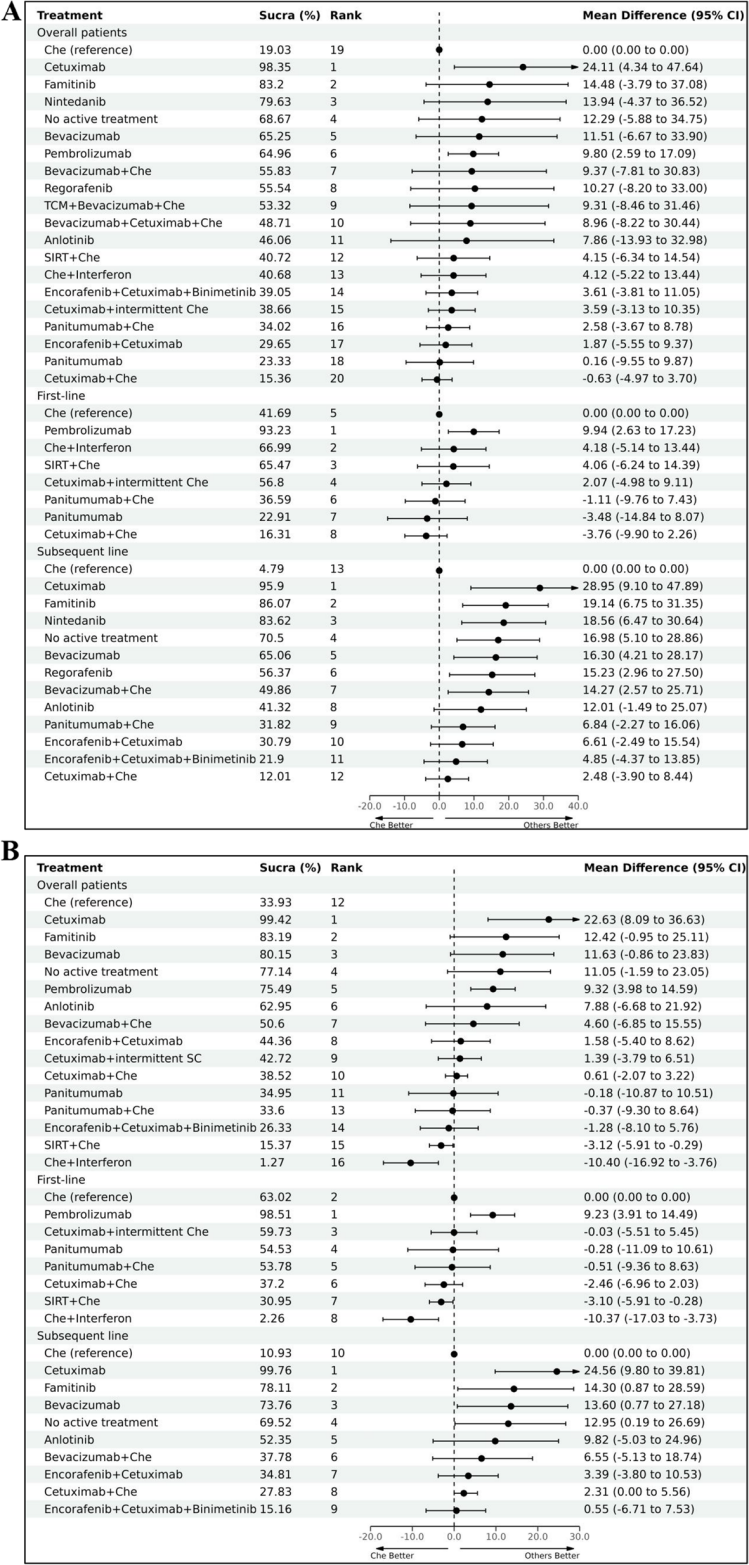
Overall analysis of QLQ-C30 (**A** long-term; **B** short-term)

In terms of QLQ-C30 GHS/QoL, long-term results are summarized in Fig. 2A, Fig. 3A and Supplementary File 7 (Fig S2). Below are the top five treatment options: cetuximab (SUCRA 98.35%), famitinib (SUCRA 83.2%), nintedanib (SUCRA 79.63%), NAT (SUCRA 68.67%) and bevacizumab (SUCRA 65.25%). Cetuximab and pembrolizumab showed significantly greater improvements compared to chemotherapy (MD 24.11, 95% CI 4.34 to 47.64 and MD 9.8, 95% CI 2.59 to 17.09, respectively). There were non-significant improvements observed for famitinib (MD 14.48, 95% CI -3.79 to 37.08), nintedanib (MD 13.94, 95% CI -4.37 to 36.52), and other treatments, with the exception of cetuximab plus chemotherapy (MD -0.63, 95% CI -4.97 to 3.7). The advantages of cetuximab and pembrolizumab compared to chemotherapy were also deemed clinically significant (a difference of 7-point is considered as clinically meaningful regarding GHS/QoL) [62]. Cetuximab showed a significant improvement in GHS/QoL compared to all other regimens, except for pembrolizumab. Short-term results were similar to long-term results. The following are the top five treatments: cetuximab (SUCRA 99.42%), famitinib (SUCRA 83.19%), bevacizumab (SUCRA 80.15%), NAT (SUCRA 77.14%) and pembrolizumab (SUCRA 75.49%). Cetuximab (MD 22.63, 95% CI 8.09 to 36.63) and pembrolizumab (MD 9.32, 95% CI 3.98 to 14.59) were the options significantly improved QLQ-C30 GHS/QoL compared with chemotherapy. Similarly, the observed enhancements had clinical significance. Interferon plus chemotherapy (MD -10.4, 95% CI -16.92 to -3.76) and SIRT plus chemotherapy (MD -3.12, 95% CI -5.91 to -0.29) was significantly inferior to chemotherapy. Similarly, cetuximab exhibited substantial enhancement in short-term GHS/QoL in comparison to all other treatments except for pembrolizumab. Detailed results can be seen in Fig. 2B, Fig. 3B and Supplementary File 7 (Fig S4).

In the long-term, compared to NAT, treatments showed significant improvements in GHS/QoL for patients who received monotherapy (MD 1.31, 95% CI 0.05 to 2.59). Likewise, monotherapy yielded better results than combination therapy (MD 2.71, 95% CI -0.44 to 4.78). In the short-term, monotherapy performed significantly better than both combination therapy (MD 5.71, 95%CI 0.78 to 10.63) and NAT (MD 3.7, 95%CI 1.41 to 6.01). More details are provided in Supplementary File 8.

**Fig. 4:**
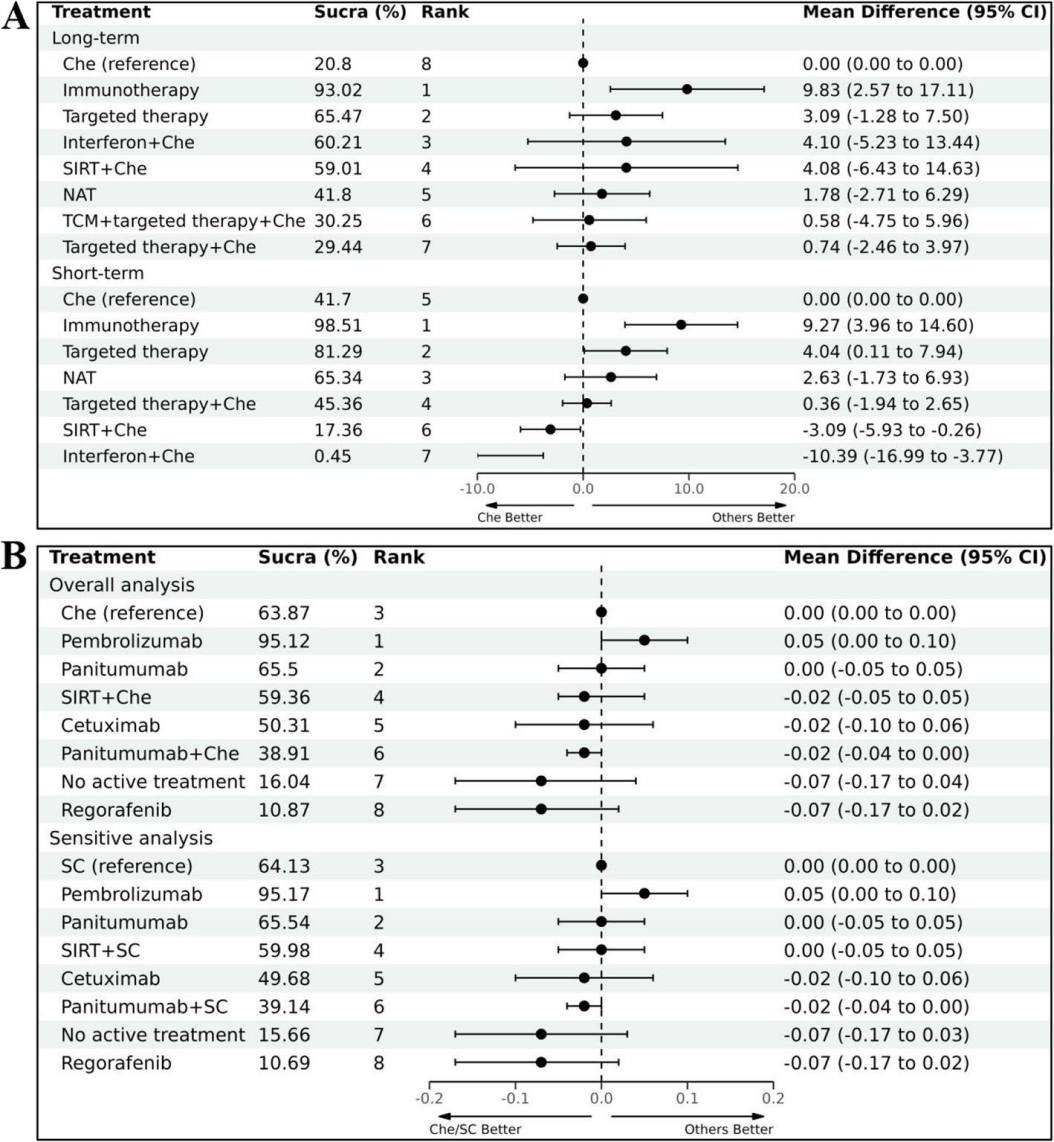
Mechanisms comparisons and comparisons of patients using EQ-5D (**A**. mechanism comparisons; **B** EQ-5D)

The comparison of mechanisms shows that immunotherapy performed the best, followed by targeted therapy. Compared to chemotherapy, treatment mechanisms with significant improvements were immunotherapy (MD 9.27, 95% CI 3.96 to 14.6) and targeted therapy (MD 4.04, 95% CI 0.11 to 7.94) for the short-term and immunotherapy (MD 9.83, 95% CI 2.57 to 17.11) for the long-term. Combination of targeted therapy with chemotherapy demonstrated inferior performance in terms of GHS/QoL. More details are provided in Fig. 2C, Fig. 2D, Fig. 4A and Supplementary File 7 (Fig S5).

Pembrolizumab performed the best in terms of EQ-5D HUS (SUCRA 95.12%) among all treatments, and it was the sole treatment that exhibited a significant advantage compared to chemotherapy (MD 0.05, 95% CI, 0 to 0.10). There is no significant difference in efficacy between other options when compared to chemotherapy. More details see Fig. 4B and Supplementary File 9.

#### Subgroup analysis

In the first-line, eight treatments were included. In the long-term, similar to the overall analysis results, pembrolizumab ranked first. It was the only option that showed a significant improvement compared to chemotherapy (MD 9.94, 95% CI 2.63 to 17.23). Conversely, cetuximab plus chemotherapy exhibited the lowest performance (VS chemotherapy: MD -3.76, 95% CI -9.9 to 2.26). For other treatments, no significant difference was observed when compared with chemotherapy. In the short-term, pembrolizumab still was the best choice (VS chemotherapy: MD 9.23, 95% CI 4.01 to 14.59). Similarly, Interferon plus chemotherapy (MD -3.1, 95% CI -5.91 to -0.28) and SIRT plus chemotherapy (MD -10.37, 95% CI -17.03 to -3.73) were the treatments that significantly worse than chemotherapy. For more information, please see Fig. 3 and Supplementary File 7 (Fig.S1, Fig.S3 and Fig.S6).

In the subsequent-line, 13 treatments were included. Long-term results showed that, cetuximab performed the best. Compared with chemotherapy, treatments associated with significantly improved GHS/QoL were cetuximab (MD 28.95, 95% CI 9.1 to 47.89), famitinib (MD 19.14, 95% CI 6.75 to 31.35), nintedanib (MD 18.56, 95% CI 6.47 to 30.64), NAT (MD 16.98, 95% CI 5.1 to 28.86), bevacizumab (MD 16.3, 95% CI 4.21 to 28.17), regorafenib (MD 15.23, 95% CI 2.96 to 27.5) and bevacizumab plus chemotherapy (MD 14.27, 95% CI 2.57 to 25.07). Short-term results were similar to the long-term, Cetuximab ranked the first, with a MD (95% CI) of 24.56 (9.8 to 39.81) compared with chemotherapy, other treatments that associated with significant improvements compared with chemotherapy were famitinib (MD 14.3, 95% CI 0.87 to 28.59), bevacizumab (MD 13.6, 95% CI 0.77 to 27.18), and NAT (MD 12.95, 95% CI 0.19 to 26.69). Both short-term and long-term results suggest that, when compared to monotherapies, combination therapies result in greater damage to HRQoL, regardless of treatment lines. More details are provided in Fig. 3 and Supplementary File 7 (Fig.S1, Fig.S3 and Fig.S6).

#### A concise approach to evidence assessment: heterogeneity, inconsistency, transitivity, and certainty

Heterogeneity test results are summarized in Supplementary Files 10. It was observed that minimal or low heterogeneity in most of the comparisons. However, moderate to high heterogeneity was detected in comparisons of:

A. Regorafenib VS NAT for the long-term QLQ-C30 GHS/QoL (I^2^ = 59%);
B. Monotherapy VS combination therapy (I^2^ = 64% for the long-term and I^2^ = 77% for the short-term); C. Monotherapy VS NAT (I^2^ = 66% for the long-term and I^2^ = 68% for the short-term) for the long-term or short-term QLQ-C30 GHS/QoL.

Results of certainty of evidence assessment are provided in Supplementary File 11, Part A. The assessment of transitivity for baseline patient age, Eastern Cooperative Oncology Group (ECOG) performance status, and the proportion of males showed significant consistency and transitivity across the included studies (Supplementary File 11, Part B). Results of the direct evidence contribution analysis can be found in Supplementary File 11, Part C.

After comparing the results from pairwise meta-analyses, we noticed that there was coherence between direct and indirect evidence. In the analysis of node splitting, we did not find any noticeable divergences between direct and indirect estimates, as all the P values in the inconsistency test were over 0.05. More details are provided in Supplementary Files 12–13. Again, for a comprehensive review of the predictive interval findings, please refer to Supplementary File 11, Part D. The trace plots indicated a satisfactory convergence of iterations, more details are provided in Supplementary File 14.

#### Sensitivity analysis

The findings here did not indicate significant deviations when compared to the overall analysis.

In this section, we distinguished between different types of chemotherapy, encompassing 33 treatments. The findings did not indicate any significant deviations when compared to the overall analysis. In the long-term, cetuximab (MD 28.95, 95% CI 9.1 to 47.89) was the only treatment that significantly improved GHS/QoL, as compared to standard chemotherapy (SC). Fluorouracil (FU), whether used alone or in combination with other drugs, caused significant damage to GHS/QoL. Compared with standard chemotherapy (SC), treatments with significant improvements for GHS/QoL were panitumumab plus irinotecan (MD 22.1, 95% CI 6.6 to 33.52), cetuximab plus irinotecan (MD 15.81, 95% CI 4.38 to 27.35), irinotecan (MD 13.1, 95% CI 3.32 to 22.8) and pembrolizumab (MD 9.81, 95% CI 2.51 to 17.07). In the first-line subgroup, pembrolizumab outperformed SC (MD 9.88, 95% CI 2.56 to 17.14) and ranked the first. Conversely, there were no notable distinctions observed in comparison with SC for other treatments. Similarly, cetuximab plus SC and FU-based treatments performed the worst. In the second-line, due to the impossibility of incorporating all schemes into one network, we constructed two separate networks. For overall patients, the top three options that showed significant improvements in terms of HRQoL when compared with SC were panitumumab plus irinotecan (MD 19.92, 95% CI 6.01 to 34.25), cetuximab plus irinotecan (MD 15.76, 95% CI 3.54 to 28.11), and irinotecan alone (MD 13.04, 95% CI 2.61 to 23.71). Cetuximab ranked first (MD 11.65, 95% CI -3.92 to 27) when compared to NAT, while no significant differences were found among the other treatments except for cetuximab plus SC (MD -15.22, 95% CI -26.97 to -2.67). The results from EQ-5D HUS indicated that pembrolizumab ranked first and was the only treatment to demonstrate significant advantages when compared to SC (MD 0.05, 95% 0 to 0.10). In the short-term, for all patients, cetuximab (MD 20.67, 95% CI 4.96 to 37.44), capecitabine plus temozolomide (MD 15.23, 95%CI 3.62 to 26.95), and pembrolizumab (MD 9.23, 95% CI 3.94 to 14.49) demonstrated significant improvements compared to SC. The combination of FU and interferon showed the poorest performance, with significant inferiority compared to SC (MD -13.65, 95% CI -24.75 to -2.52). Within the first-line subgroup, pembrolizumab demonstrated the highest ranking and exhibited notable improvements compared to SC (MD 9.28, 95% CI 4.01 to 14.59). No significant differences were observed between the other regimens when compared with SC, except for the combination of FU plus interferon (MD -13.86, 95% CI -25.03 to -2.81). In the second-line subgroup, cetuximab ranked first and was the only treatment to demonstrate significant advantages when compared with NAT (MD 11.54, 95% CI 4.48 to 18.55), followed by famitinib and bevacizumab. Bevacizumab plus SC (MD -6.45, 95% CI -11.84 to -1.06) was found to be significantly worse than NAT. More information is presented in Supplementary File 15.

After employing random-effects models, we found that the overall conclusions were largely consistent with those from the fixed-effects models, but with diminished differences in efficacy between treatments. Specifically, the results indicated that monotherapy consistently outperformed other approaches in improving patients' HRQoL, both in the short and long term, followed by NAT, with combination therapy showing the least benefit. In terms of treatment mechanisms, immunotherapy and targeted therapy remained the most effective, regardless of the duration, while multi-mechanism treatment regimens were the least effective. Finally, at the level of specific treatments and without distinguishing baseline gene expression, cetuximab continued to show the best performance in the overall population; pembrolizumab was the best performer in first-line treatments, and cetuximab remained the optimal choice in subsequent lines of therapy regarding HRQoL, both in the short and long term. More details are provided in Supplementary File 16.

After excluding trials targeting on BRAF/RAS mutated and MSI-H patients, similar to overall analysis, cetuximab (MD 25.63, 95% CI 8.13 to 44.84) and famitinib (MD 16.03, 95% CI 0.43 to 33.88) were the treatments that showed significant improvements compared to chemotherapy. In the first-line subgroup, there were no significant differences among any of included treatments. In the subsequent-line subgroup, cetuximab was the best choice. See more information in Supplementary File 17.

When we only considered clinical evidence from studies where patients' baseline gene expression levels were not specified, we observed that compared to chemotherapy, certain treatments notably improved GHS/QoL. For patients who received cetuximab, the MD was 26.71 with a 95% confidence interval (CI) ranging from -1.28 to 44.44, followed by famitinib (MD 17.82, 95% CI -9.74 to 33.85) and nintedanib (MD 17.19, 95% CI -10.38 to 33.29). Specifically, in the first-line, chemotherapy combined with interferon showed the best performance, followed by SIRT combined with chemotherapy. However, it should be noted that there were no significant differences in the impact on HRQoL between these regimens. In subsequent lines, cetuximab emerged as the optimal choice, with chemotherapy and chemo-combination therapies significantly underperforming compared to targeted monotherapy regarding HRQoL. More details are provided in Supplementary File 18.

The comparison for KRAS wild-type patients, as indicated by the QLQ-C30 scores, suggests that chemotherapy had some advantages over panitumumab alone or the combination of panitumumab or cetuximab with chemotherapy, although there were no significant differences between these regimens. Similarly, results from the EQ-5D HUS indicated that chemotherapy had a certain advantage over the combination of panitumumab with chemotherapy (MD, 0.02; 95% CI, -0.00 to 0.04); at the same time, cetuximab had an edge over regorafenib (MD, 0.05; 95% CI, -0.00 to 0.11), and panitumumab was superior to NAT (MD, 0.17; 95% CI, -0.08 to 0.42). Unfortunately, due to the lack of sufficient clinical evidence, we were unable to make a more systematic comparison. More details are provided in Supplementary File 19.

## Discussion

### Main findings

Impacts of systematic treatments on HRQoL for patients with mCRC is comprehensively analyzed in this NMA. The key findings of this study are summarized as follows:

1. Monotherapy was associated with significant improvements in patient HRQoL compared with combination therapy and NAT.
2. Immunotherapy and targeted therapy exhibited the most favorable outcomes regarding HRQoL. Incorporating targeted therapy with chemotherapy did not enhance patient performance in terms of HRQoL.
3. In managing mCRC, cetuximab was particularly effective in enhancing HRQoL when gene expression baselines were unspecified, especially for those previously received systematic treatment. Considering HRQoL, efficacy, and safety collectively, cetuximab proved to be an advantageous treatment for patients with wild-type KRAS mutations [46, 63, 64].

The sensitivity analyses demonstrated that the overall results were robust. That is, after distinguishing between chemotherapy medications and excluding RCTs with inconsistent baseline characteristics, the overall conclusions remained unchanged.

Several explanations may account for our findings. Firstly, monotherapy outperformed combination therapy and NAT in this study. Firstly, drugs has the potential to inhibit the growth and spread of tumor cells, delaying the progression of the patient's disease [65]. Innovative treatments that can effectively control patients' conditions have emerged nowadays, such as immunotherapy [66], and novel targeted drugs [65]. Taking these regimens can improve the patients' physical condition and alleviate the burden of diseases, while also enhancing their mental well-being [22]. For mCRC patients, who already have poor physical condition, medications may induce severe adverse reactions (SAE). Additionally, more frequent use of medications would result in more hospitalizations or monitoring, causing inconvenience in patient life. Additionally, these medications may come with significant costs, increasing patients' psychological burden [67]. These aspects are indeed important components of HRQoL [6]. Monotherapy was observed to have fewer SAEs compared to combination therapy for mCRC patients [68], while SAEs are one of the most detrimental factors affecting HRQoL [69]. Therefore, for patients receiving monotherapy, HRQoL is likely to be higher compared to combination therapy, provided that the efficacy is ensured. It is worth noting that in this study, NAT is not a non-treatment, but a palliative therapy. Compared to combination therapy, NAT may have a lower efficacy but also avoids the potential occurrence of drug-related SAEs [31, 65, 70, 71]. This might also be a reason why patients treated by multiple mechanisms therapy had worse HRQoL when compared to single mechanism therapy. Cetuximab, an EGFR inhibitor, was significantly associated with higher tumor response. Additionally, it enhanced and expedited symptom relief in patients whose tumors had responded [46, 63]. Cetuximab had low incidences of SAEs, such as skin rash, infusion reactions, and gastrointestinal toxicity [64]. There could be several reasons why cetuximab has shown significant improvements in patient HRQoL. The benefits of cetuximab are particularly remarkable in patients with wild-type KRAS status [46]. Pembrolizumab significantly enhancing the HRQoL for patients, particularly those undergoing first-line treatment with high tumor mutation burden. Possible reasons are as follows: According to KEYNOTE-177 trial, pembrolizumab improved survival rate, and had fewer SAEs, such as diarrhea, fatigue, nausea, vomiting, and decreased appetite [66]. Consistent with our findings, improvements were generally observed in the corresponding symptom scores of the EORTC QLQ-C30 [22]. Chemotherapy, especially FU-based combination therapy, takes more infusion times, longer hospital stays for infusion, and shorter intervals between treatments compared to immunotherapy and targeted therapy. The occurrence and burden of chemotherapy are higher in vomiting, loss of appetite, fatigue, nausea and diarrhea [22].

More recent, encorafenib plus cetuximab with or without binimetinib compared to cetuximab in combination with chemotherapy showed significant improvement in mortality. However, this novel option did not improve the HRQoL for the patients [21]. Trifluridine/tipiracil, also known as TA-102, has shown superiority over bevacizumab in terms of progression-free survival. However, the addition of TA-102 has not improved HRQoL for patient [72]. According to CheckMate 142, a non-randomized study, nivolumab with or without ipilimumab demonstrated significant improvements in patients with MSI-H/dMMR mCRC [73].

Our study had several strengths: We compared impacts of drug mechanisms, drug quantities, and specific medication on HRQoL of mCRC patients for the first time. In order to obtain reliable conclusions, we used multiple outcome indicators, and conducted subgroup and scenario analyses to reduce the uncertainty caused by heterogeneity. Patient-reported outcomes provide valuable insights that surpass assessments made by clinicians, and HRQoL is greatly appreciated in the decision-making process in oncology [74]. Our analysis can provide valuable information for patients and clinicians in clinical medication and disease management.

### Limitations

HRQoL studies face a significant challenge due to the absence of data [75–77], particularly in settings of advanced stage. Over time, there has been a decline in questionnaire compliance, which has restricted the collection of HRQoL data. Thus, many current treatment strategies lack HRQoL data. This also leads to the following limitations in our research: We didn’t make indirect comparison based on the mutation target or gene expression levels of patients. The majority of the included RCTs did not differentiate between mutation types and gene expression levels when reported HRQoL. There are few studies reported HRQoL for patients with BRAF V600E-mutant [21], MSI-H/dMMR or KRAS mutation [22, 23], and several studies which reported data targeting wild-type RAS or KRAS patients cannot establish an effective network [34, 39, 43, 45, 57]. Most of the HRQoL-related evidence in this study was derived from populations without specified baseline expression levels of patients. Therefore, additional clinical evidence is required to validate the performance of different therapeutic modalities within populations characterized by specific target expression levels. For example, patients in Keynote 177 trial is MSI-H/dMMR [22], the impact of pembrolizumab on microsatellite stable patients is still unclear. Lack of data also prevented us from considering more detailed aspects of HRQoL, such as physical functioning, social functioning, and fatigue scores. Additionally, fixed-effects models were used for this NMA, considering the included studies had low heterogeneity. When networks are sparse, random-effect models might result in overly wide credible intervals, which could lead to unrealistic estimates in NMA [78]. Finally, it should be noted that our protocol was registered at a very late stage and the subgroup analyses were not pre-specified.

## Conclusions

In terms of patient HRQoL, targeted therapies and immunotherapies yield the most favorable outcomes, whereas complex treatment regimens are less effective. Monotherapy, particularly with cetuximab or pembrolizumab, significantly improves HRQoL over combination therapies or NAT. This review is focused strictly on HRQoL results, excluding other clinical endpoints.

### Supplementary Information

**Additional file 1: Supplementary File 1.** Checklist of the PRISMA Extension for Systematic Review and Network Meta-analysis. **Supplementary File 2.** Search Terms for Meta-analysis (Pubmed). **Supplementary File 3.** Definitions of Standard Chemotherapy and Regimen Information. **Supplementary File 4.** Other Characteristics of the Included Studies. **Supplementary File 5.** Cochrane Risk of the Bias Assessment Tool. **Supplementary File 6.** Funnel Plots to Show Publication Bias. **Supplementary File 7.** League Tables and Network Plots for Subgroup Analysis. **Supplementary File 8.** Summarized Result of Comparison of Monotherapy, Combination Therapy and No Active Treatment. **Supplementary File 9.** Summarized Result of EQ-5D. **Supplementary File 10.** Heterogeneity Assessment Results. **Supplementary File 11.** Evidence Level Assessment (A), Transitivity (B), Direct Evidence Contribution (C), Predictive Interval (D). **Supplementary File 12.** Forest Plots Depicting Results of Head-to-head Comparisons. **Supplementary File 14.** Brooks-Gelman-Rubin Diagnostic. **Supplementary File 15.** Summarized Results of Sensitivity Analysis, Part 1 (QLQ-C30, Differentiating Chemotherapy). **Supplementary File 16.** Summarized Results of Sensitivity Analysis, Part 2 (QLQ-C30, Random Effects Models). **Supplementary File 17.** Summarized Results of Sensitivity Analysis, Part 3 (QLQ-C30, Long-term, Excluding RCTs Targeting on BRAF/RAS Mutated and MSI-H Patients). **Supplementary File 18.** Summarized Results of Sensitivity Analysis, Part 4 (QLQ-C30, Long-term, Evidence from Randomized Controlled Trials Focused Solely on Patients with Unrestricted Gene Expression). **Supplementary File 19.** Summarized Results of Sensitivity Analysis, Part 5 (QLQ-C30, Long-term, Focused on Wild-type KRAS Patients).

## Data Availability

Full data set is available, on request from the corresponding author, e-mail, zhengxp@njucm.edu.cn.
