# Supplementary material for: Impacts of systemic treatments on health-related quality of life for patients with metastatic colorectal cancer: a systematic review and network meta-analysis
Source: BMC Cancer. 2024 Feb 9;24:188. doi: 10.1186/s12885-024-11937-z (PMC10854105; doi:10.1186/s12885-024-11937-z)
Supplement: Supplementary file 1 — Additional file 1: Supplementary File 1. Checklist of the PRISMA Extension for Systematic Review and Network Meta-analysis. Supplementary File 2. Search Terms for Meta-analysis (Pubmed). Supplementary File 3. Definitions of Standard Chemotherapy and Regimen Information. Supplementary File 4. Other Characteristics of the Included Studies. Supplementary File 5. Cochrane Risk of the Bias Assessment Tool. Supplementary File 6. Funnel Plots to Show Publication Bias. Supplementary File 7. League Tables and Network Plots for Subgroup Analysis. Supplementary File 8. Summarized Result of Comparison of Monotherapy, Combination Therapy and No Active Treatment. Supplementary File 9. Summarized Result of EQ-5D. Supplementary File 10. Heterogeneity Assessment Results. Supplementary File 11. Evidence Level Assessment (A), Transitivity (B), Direct Evidence Contribution (C), Predictive Interval (D). Supplementary File 12. Forest Plots Depicting Results of Head-to-head Comparisons. Supplementary File 14. Brooks-Gelman-Rubin Diagnostic. Supplementary File 15. Summarized Results of Sensitivity Analysis, Part 1 (QLQ-C30, Differentiating Chemotherapy). Supplementary File 16. Summarized Results of Sensitivity Analysis, Part 2 (QLQ-C30, Random Effects Models). Supplementary File 17. Summarized Results of Sensitivity Analysis, Part 3 (QLQ-C30, Long-term, Excluding RCTs Targeting on BRAF/RAS Mutated and MSI-H Patients). Supplementary File 18. Summarized Results of Sensitivity Analysis, Part 4 (QLQ-C30, Long-term, Evidence from Randomized Controlled Trials Focused Solely on Patients with Unrestricted Gene Expression). Supplementary File 19. Summarized Results of Sensitivity Analysis, Part 5 (QLQ-C30, Long-term, Focused on Wild-type KRAS Patients). [file 12885_2024_11937_MOESM1_ESM.docx]

## Supplementary Appendix

[Supplementary Appendix 1](#_Toc21364)

[Supplementary File 1. Checklist of the PRISMA Extension for Systematic Review and Network Meta-analysis 2](#_Toc24951)

[Supplementary File 2. Search Terms for Meta-analysis (Pubmed) 7](#_Toc22040)

[Supplementary File 3. Definitions of Standard Chemotherapy and Regimen Information 8](#_Toc2729)

[Supplementary File 4 Other Characteristics of the Included Studies 10](#_Toc10821)

[Supplementary File 5. Cochrane Risk of the Bias Assessment Tool 13](#_Toc30066)

[Supplementary File 6. Funnel Plots to Show Publication Bias 14](#_Toc12314)

[Supplementary File 7. League Tables and Network Plots for Subgroup Analysis 17](#_Toc7434)

[Supplementary File 8 Summarized Result of Comparison of Monotherapy, Combination Therapy and No Active Treatment 30](#_Toc27445)

[Supplementary File 9: Summarized Result of EQ-5D 31](#_Toc16051)

[Supplementary File 10 Heterogeneity Assessment Results 34](#_Toc21352)

[Supplementary File 11 Evidence Level Assessment (A), Transitivity (B), Direct Evidence Contribution (C), Predictive Interval (D) 36](#_Toc19502)

[Supplementary File 12. Forest Plots Depicting Results of Head-to-head Comparisons 103](#_Toc20444)

[Supplementary File 14. Brooks-Gelman-Rubin Diagnostic 118](#_Toc705)

[Supplementary File 15 Summarized Results of Sensitivity Analysis, Part 1 (QLQ-C30, Differentiating Chemotherapy) 128](#_Toc22653)

[Supplementary File 16 Summarized Results of Sensitivity Analysis, Part 2 (QLQ-C30, Random Effects Models) 136](#_Toc21367)

[Supplementary File 17 Summarized Results of Sensitivity Analysis, Part 3 (QLQ-C30, Long-term, Excluding RCTs Targeting on BRAF/RAS Mutated and MSI-H Patients) 140](#_Toc28307)

[Supplementary File 18 Summarized Results of Sensitivity Analysis, Part 4 (QLQ-C30, Long-term, Evidence from Randomized Controlled Trials Focused Solely on Patients with Unrestricted Gene Expression) 144](#_Toc30336)

[Supplementary File 19 Summarized Results of Sensitivity Analysis, Part 5 (QLQ-C30, Long-term, Focused on Wild-type KRAS Patients) 145](#_Toc4926)

# **Supplementary File 1. Checklist of the PRISMA Extension for Systematic Review and Network Meta-analysis**

| **Section/Topic** | **Item #** | **Checklist Item** | **Reported on Page #** |
| --- | --- | --- | --- |
| **TITLE** |  |  |  |
| Title | 1 | Identify the report as a systematic review *incorporating a network meta-analysis (or related form of meta-analysis).* | ***1*** |
|  |  |  |  |
| **ABSTRACT** |  |  |  |
| Structured summary | 2 | Provide a structured summary including, as applicable:  **Background:** main objectives  **Methods:** data sources; study eligibility criteria, participants, and interventions; study appraisal; and *synthesis methods, such as network meta-analysis.*  **Results:** number of studies and participants identified; summary estimates with corresponding confidence/credible intervals; *treatment rankings may also be discussed. Authors may choose to summarize pairwise comparisons against a chosen treatment included in their analyses for brevity.*  **Discussion/Conclusions:** limitations; conclusions and implications of findings.  **Other:** primary source of funding; systematic review registration number with registry name. | 2 |
|  |  |  |  |
| **INTRODUCTION** |  |  |  |
| Rationale | 3 | Describe the rationale for the review in the context of what is already known*, including mention of why a network meta-analysis has been conducted.* | ***3*** |
| Objectives | 4 | Provide an explicit statement of questions being addressed, with reference to participants, interventions, comparisons, outcomes, and study design (PICOS). | 3 |
|  |  |  |  |
| **METHODS** |  |  |  |
| Protocol and registration | 5 | Indicate whether a review protocol exists and if and where it can be accessed (e.g., Web address); and, if available, provide registration information, including registration number. | 3 |
| Eligibility criteria | 6 | Specify study characteristics (e.g., PICOS, length of follow-up) and report characteristics (e.g., years considered, language, publication status) used as criteria for eligibility, giving rationale. *Clearly describe eligible treatments included in the treatment network, and note whether any have been clustered or merged into the same node (with justification).* | ***4*** |
| Information sources | 7 | Describe all information sources (e.g., databases with dates of coverage, contact with study authors to identify additional studies) in the search and date last searched. | 4 |
| Search | 8 | Present full electronic search strategy for at least one database, including any limits used, such that it could be repeated. | 4 |
| Study selection | 9 | State the process for selecting studies (i.e., screening, eligibility, included in systematic review, and, if applicable, included in the meta-analysis). | 4 |
| Data collection process | 10 | Describe method of data extraction from reports (e.g., piloted forms, independently, in duplicate) and any processes for obtaining and confirming data from investigators. | 4 |
| Data items | 11 | List and define all variables for which data were sought (e.g., PICOS, funding sources) and any assumptions and simplifications made. | 4 |
| **Geometry of the network** | **S1** | Describe methods used to explore the geometry of the treatment network under study and potential biases related to it. This should include how the evidence base has been graphically summarized for presentation, and what characteristics were compiled and used to describe the evidence base to readers. | ***4-5*** |
| Risk of bias within individual studies | 12 | Describe methods used for assessing risk of bias of individual studies (including specification of whether this was done at the study or outcome level), and how this information is to be used in any data synthesis. | 4 |
| Summary measures | 13 | State the principal summary measures (e.g., risk ratio, difference in means). *Also describe the use of additional summary measures assessed, such as treatment rankings and surface under the cumulative ranking curve (SUCRA) values, as well as modified approaches used to present summary findings from meta-analyses.* | 4-5 |
| Planned methods of analysis | 14 | Describe the methods of handling data and combining results of studies for each network meta-analysis. This should include, but not be limited to:   - *Handling of multi-arm trials;* - *Selection of variance structure;* - *Selection of prior distributions in Bayesian analyses; and* - *Assessment of model fit.* | 4-5 |
| **Assessment of Inconsistency** | **S2** | Describe the statistical methods used to evaluate the agreement of direct and indirect evidence in the treatment network(s) studied. Describe efforts taken to address its presence when found. | 5 |
| Risk of bias across studies | 15 | Specify any assessment of risk of bias that may affect the cumulative evidence (e.g., publication bias, selective reporting within studies). | **4** |
| Additional analyses | 16 | Describe methods of additional analyses if done, indicating which were pre-specified. This may include, but not be limited to, the following:   - Sensitivity or subgroup analyses; - Meta-regression analyses; - *Alternative formulations of the treatment network; and* - *Use of alternative prior distributions for Bayesian analyses (if applicable).* | ***4-5*** |
|  |  |  |  |
| **RESULTS†** |  |  |  |
| Study selection | 17 | Give numbers of studies screened, assessed for eligibility, and included in the review, with reasons for exclusions at each stage, ideally with a flow diagram. | 5 |
| **Presentation of network structure** | **S3** | Provide a network graph of the included studies to enable visualization of the geometry of the treatment network. | ***5*** |
| **Summary of network geometry** | **S4** | Provide a brief overview of characteristics of the treatment network. This may include commentary on the abundance of trials and randomized patients for the different interventions and pairwise comparisons in the network, gaps of evidence in the treatment network, and potential biases reflected by the network structure. | ***5*** |
| Study characteristics | 18 | For each study, present characteristics for which data were extracted (e.g., study size, PICOS, follow-up period) and provide the citations. | 5 |
| Risk of bias within studies | 19 | Present data on risk of bias of each study and, if available, any outcome level assessment. | 5 |
| Results of individual studies | 20 | For all outcomes considered (benefits or harms), present, for each study: 1) simple summary data for each intervention group, and 2) effect estimates and confidence intervals. *Modified approaches may be needed to deal with information from larger networks.* | ***5-7*** |
| Synthesis of results | 21 | Present results of each meta-analysis done, including confidence/credible intervals. *In larger networks, authors may focus on comparisons versus a particular comparator (e.g. placebo or standard care), with full findings presented in an appendix. League tables and forest plots may be considered to summarize pairwise comparisons.* If additional summary measures were explored (such as treatment rankings), these should also be presented. | ***5-7*** |
| **Exploration for inconsistency** | **S5** | Describe results from investigations of inconsistency. This may include such information as measures of model fit to compare consistency and inconsistency models, *P* values from statistical tests, or summary of inconsistency estimates from different parts of the treatment network. | ***7*** |
| Risk of bias across studies | 22 | Present results of any assessment of risk of bias across studies for the evidence base being studied. | 5 |
| Results of additional analyses | 23 | Give results of additional analyses, if done (e.g., sensitivity or subgroup analyses, meta-regression analyses*, alternative network geometries studied, alternative choice of prior distributions for Bayesian analyses,* and so forth). | ***7*** |
|  |  |  |  |
| **DISCUSSION** |  |  |  |
| Summary of evidence | 24 | Summarize the main findings, including the strength of evidence for each main outcome; consider their relevance to key groups (e.g., healthcare providers, users, and policy-makers). | 8 |
| Limitations | 25 | Discuss limitations at study and outcome level (e.g., risk of bias), and at review level (e.g., incomplete retrieval of identified research, reporting bias). *Comment on the validity of the assumptions, such as transitivity and consistency. Comment on any concerns regarding network geometry (e.g., avoidance of certain comparisons).* | 9-10 |
| Conclusions | 26 | Provide a general interpretation of the results in the context of other evidence, and implications for future research. | 10 |
|  |  |  |  |
| **FUNDING** |  |  |  |
| Funding | 27 | Describe sources of funding for the systematic review and other support (e.g., supply of data); role of funders for the systematic review. This should also include information regarding whether funding has been received from manufacturers of treatments in the network and/or whether some of the authors are content experts with professional conflicts of interest that could affect use of treatments in the network. | ***1*** |

PICOS = population, intervention, comparators, outcomes, study design.

* Text in italics indicateS wording specific to reporting of network meta-analyses that has been added to guidance from the PRISMA statement.

† Authors may wish to plan for use of appendices to present all relevant information in full detail for items in this section.

*The Checklist of the PRISMA extension is formulated for RICE-META1st with the structure of PRISMA NMA Checklist of

Items to Include When Reporting a Systematic Review Involving a Network Meta-analysis in https://prismastatement.org/Extensions/NetworkMetaAnalysis

# **Supplementary File 2. Search Terms for Meta-analysis (Pubmed)**

1. ((((colorectal cancer[Title]) AND (English[Language])) AND (metastatic[Title/Abstract] OR advanced[Title/Abstract])) ) AND (quality of life[Title/Abstract] OR patient report outcome[Title/Abstract] OR Quality-of-life[Title/Abstract] OR patient-reported outcome[Title/Abstract]);
2. ("colorectal cancer" [Title] OR "colorectal adenocarcinoma"[Title] OR "colorectal carcinoma"[Title] OR "colorectal liver metastases"[Title]) AND ("metastatic"[Title/Abstract] OR "advanced"[Title/Abstract]) AND "English"[Language] AND ("compare"[Title/Abstract] OR "comparison"[Title/Abstract] OR "comparative"[Title/Abstract] OR "comparing"[Title/Abstract] OR "versus"[Title/Abstract] OR "vs"[Title/Abstract]) AND ("randomized controlled trial"[Publication Type] OR "controlled clinical trial"[Publication Type] OR "Randomized"[Title/Abstract] OR "randomised"[Title/Abstract] OR "randomly"[Title/Abstract] OR "Trial"[Title/Abstract] OR "phase"[Title/Abstract]) AND ("quality-of-life"[Text Word] OR "quality of life"[Text Word]).

# **Supplementary File 3. Definitions of Standard Chemotherapy and Regimen Information**

Standard chemotherapy: mFOLFOX6, FOLFOX4, FOLFOX, FLOX, FOLFIRI, CAPEOX, XELOX or OXXEL

| Treatment | Medication details |
| --- | --- |
| 8 cycles irinotecan | Irinotecan was paused after eight 3-week cycles |
| Anlotinib | 12 mg/day |
| Bevacizumab | 5 mg/kg |
| Bevacizumab 4d before SC | bevacizumab administered 4 days before chemotherapy |
| Binimetinib | 45 mg twice a day |
| capecitabine | 750 mg/m^2^, twice daily |
| Cetuximab | 500 mg/m^2^ |
| Encorafenib | 300 mg/day |
| Famitinib | 25 mg/day |
| FOLFIRI | irinotecan 180 mg/m^2^ on day 1, leucovorin 200 mg/m^2^ on days 1 and 2, followed by 5-fluorouracil 400 mg/m^2^ bolus and then 5-FU 600 mg/m^2^ both on days 1 and 2, every two weeks |
| FOLFOX/FLOX | every 2 weeks with oxaliplatin 85 mg/m^2^ on day 1, and 5-FU 500 mg/m^2^ bolus injection followed 30 min later by FA 60 mg/m^2^ bolus on days 1 and 2 |
| FOLFOX-4 | oxaliplatin, 85 mg/m^2^ at day 1, and leucovorin calcium, 200 mg/m^2^, and fluorouracil, 400-mg/m^2^ bolus followed by 600-mg/m^2^ continuous 24-hour infusion at days 1 and 2, every 2 weeks |
| FU | 2,600 mg/m^2^ as a 24-hour infusion alone weekly |
| FU+LV | Fluorouracil 425 mg/m^2^ i.v. weekly; leucovorin 20 mg/m^2^ on days 1 to 5 and repeated on day 28 |
| FU24h+LV | FU24h in combination with 500 mg/m^2^ leucovorin weekly |
| Interferon | a dose of 5 MU subcutaneously three times weekly |
| Intermittent SC | Nordic FLOX was paused after 8 cycles, q3w |
| Irinotecan | 300 or 350 mg/m2 every 3 weeks |
| modified CAPOX/XELOX | intravenous oxaliplatin, 85 mg/m^2^, on day 1 plus oral capecitabine, 1000 mg/m^2^, twice daily on days 1 to 10) |
| modified FOLFOX-6 | intravenous oxaliplatin, 85 mg/m^2^, on day 1, followed by intravenous levo–folinic acid, 200 mg/m^2^,plus bolus fluorouracil, 400 mg/m^2^, and a 46-hour intravenous administration of fluorouracil, 2400mg/m^2^ |
| Nintedanib | 200 mg twice daily |
| OXA(OXAFAFU) | Oxaliplatin 85 mg/m^2^ i.v. on day 1; 6S-leucovo rin 250 mg/m^2^ i.v. (2 h), followed by Xuorouracil 850 mg/m^2^ i.v. bolus on day 2, every 2 weeks |
| oxaliplatin | 85 mg/m^2^, per two weeks |
| OXXEL | oxaliplatin 100 mg/m^2^ i.v on day 1; capecitabine 1,000 mg/m^2^ orally twice daily from day 1 to day 11, every 2 weeks |
| Panitumumab | 6 mg/kg [1-hour infusion for the first administration; 30-minute infusion thereafter] |
| Pembrolizumab | 200 mg every 3 weeks |
| Regorafenib | 160 mg/day |
| S1 | full-dose: 30 mg/m^2^ orally twice daily on days 1~14,q3w;reduced-dose S1: 20 mg/m^2^ orally twice daily on days 1~14, q3w* |
| SIRT | Selective internal radiotherapy (SIRT) is an involving injection of yttrium-90 microspheres |
| SOx | S1 20 mg/m^2^ orally twice daily on days 1~14+oxaliplatin 100 mg/m^2^ intravenously on day 1, q3w (once every 3 weeks) |
| temozolomide | 75 mg/m^2^, twice daily |
| Traditional Chinese medicine | Huangci Granule,12 g, twice a day |

*q3w: once every three weeks

# **Supplementary File 4 Other Characteristics of the Included Studies**

| Trial | ECOG performance status≥1(%) | | Year | Region | Target mutation or gene expression level | Networks |
| --- | --- | --- | --- | --- | --- | --- |
|  | I | C |  |  |  |  |
| Antonio 2021[1] | 22 | 21 | 2021 | Europe | No limited | Overall analysis for QLQ-C30 GHS (short-term and long-term) |
| ALTER0703[2] | 77 | 70 | 2021 | Asia | No limited | Overall and subsequent line analyses for QLQ-C30 GHS (short-term and long-term) |
| Liu 2020[3] | 49 | 42 | 2020 | Asia | No limited | Overall analysis for QLQ-C30 GHS (long-term) |
| CONCUR[4, 5] | 72 | 78 | 2020 | Global | No limited | Overall and subsequent line analyses for QLQ-C30 GHS (short-term and long-term) and EQ-5D |
| Xu 2017[6] | 82 | 84 | 2017 | Asia | No limited | Overall and subsequent line analyses for QLQ-C30 GHS (short-term and long-term) |
| BEACON CRC[7] | 48/49/51 | | 2022 | Global | BRAF V600E-mutant | Overall and subsequent line analyses for QLQ-C30 GHS (short-term and long-term) |
| PRODIGE 18[8] | 45 | 43 | 2022 | Europe | No limited | Overall and subsequent line analyses for QLQ-C30 GHS (short-term and long-term) |
| NORDIC9[9] | 69 | 60 | 2021 | Europe | No limited | Overall analysis for QLQ-C30 GHS (short-term and long-term) |
| KEYNOTE-177[10] | 51 | 46 | 2021 | Global | MSI-H/dMMR | Overall and first-line analyses for QLQ-C30 GHS (short-term and long-term) and EQ-5D |
| Valentino [11] | 26 | 26 | 2020 | Europe | RAS wild-type | Overall and first-line analyses for QLQ-C30 GHS (short-term and long-term) and EQ-5D |
| Jane 2020[12] | NA | NA | 2020 | Global | No limited | Overall and first-line analyses for QLQ-C30 GHS (short-term and long-term) and EQ-5D |
| Filippo 2020[13] | 44 | 49 | 2020 | Europe | RAS mutated | Overall and subsequent line analyses for QLQ-C30 GHS (short-term and long-term) and EQ-5D |
| REVERCE  [14] | 33 | 22 | 2019 | Asia | KRAS wild-type | Overall analysis for EQ-5D |
| NORDIC-VII[15] | 33/32/33 | | 2017 | Europe | No limited | Overall and first-line analyses for QLQ-C30 GHS (short-term and long-term) and EQ-5D |
| CRYSTAL Subgroup  [16] | 44 | 40 | 2017 | Global | RAS Wild-Type Metastatic | Overall and first-line analyses for QLQ-C30 GHS (short-term and long-term) and EQ-5D |
| AIO KRK 0207[17] | 52/54/62 | | 2016 | Global | No limited | Overall and subsequent line analyses for QLQ-C30 GHS (short-term and long-term) and EQ-5D |
| CRYSTAL Subgroup  [18] | 41 | 43 | 2012 | Global | KRAS Wild-Type Metastatic | Overall and first-line analyses for QLQ-C30 GHS (short-term and long-term) and EQ-5D |
| Yong 2013[19] | 70 | 78 | 2013 | Asia | No limited | Overall and first-line analyses for QLQ-C30 GHS (short-term and long-term) and EQ-5D |
| PRIME[20] | NA | NA | 2011 | Global | wild-type KRAS | Overall analysis for EQ-5D |
| PRIME[20] | NA | NA | 2011 | Global | wild-type KRAS | Overall analysis for EQ-5D |
| DaVINCI  [21] | NA | NA | 2011 | Oceania | No limited | Overall and subsequent line analyses for QLQ-C30 GHS (long-term) |
| Dawn 2010[22] | 54 | 63 | 2011 | Global | KRAS wild-type | Overall analysis for EQ-5D |
| CO.17 Trial[23] | NA | NA | 2009 | Global | No limited | Overall and subsequent line analyses for QLQ-C30 GHS (short-term and long-term) |
| Jolien 2009[24] | 41 | 34 | 2009 | Europe | No limited | Overall and first-line analyses for QLQ-C30 GHS (long-term) and EQ-5D |
| Southern Italy Cooperative Oncology study 0401[25] | 40 | 39 | 2009 | Europe | No limited | Overall and first-line analyses for QLQ-C30 GHS (short-term and long-term) |
| EPIC[26] | NA | NA | 2008 | Global | No limited | Overall and subsequent line analyses for QLQ-C30 GHS (short-term and long-term) |
| Rohit 2004[27] | 64 | 56 | 2004 | Europe | No limited | Overall and subsequent line analyses for QLQ-C30 GHS (short-term and long-term) |
| EORTCG Group Study 40952[28] | 46/48/48 | | 2003 | Europe | No limited | Overall and first-line analyses for QLQ-C30 GHS (short-term and long-term) |
| Charles 2003[29] | 51 | 56 | 2003 | America | No limited | Overall and subsequent line analyses for QLQ-C30 GHS (short-term and long-term) |
| Saltz 2000[30] | 61 | 58 | 2000 | Global | No limited | Overall and first-line analyses for QLQ-C30 GHS (short-term and long-term) |
| Hill 1995[31] | 83 | 79 | 1995 | Europe | No limited | Overall and first-line analyses for QLQ-C30 GHS (short-term and long-term) |
| ASPECCT  [32] | 69 | 67 | 2014 | Global | No limited | Overall analysis for EQ-5D |
| Peeters 2014[33] | NA | NA | 2014 | Global | No limited | Overall analysis for EQ-5D |
| PICCOLO  [34] | NA | NA | 2013 | Europe | KRAS wild-type | Overall and subsequent line analyses for QLQ-C30 GHS (long-term) |
| LUME-Colon 1[35] | 58 | 63 | 2018 | Global | No limited | Overall and subsequent line analyses for QLQ-C30 GHS (long-term) |
| CORRECT  [36] | 48 | 43 | 2013 | Global | No limited | Overall and subsequent line analyses for QLQ-C30 GHS (long-term) and EQ-5D HU |
| Yong 2012[37] | NA | NA | 2012 | Asia | No limited | Overall and first-line analyses for QLQ-C30 GHS (long-term) |
| PanaMa[38] | 43 | 38 | 2023 | Global | No limited | Overall and first-line analyses for QLQ-C30 GHS (short-term and long-term) |

# **Supplementary File 5. Cochrane Risk of the Bias Assessment Tool**

**Fig.S1 Risk of the Bias Assessment for Each Study**


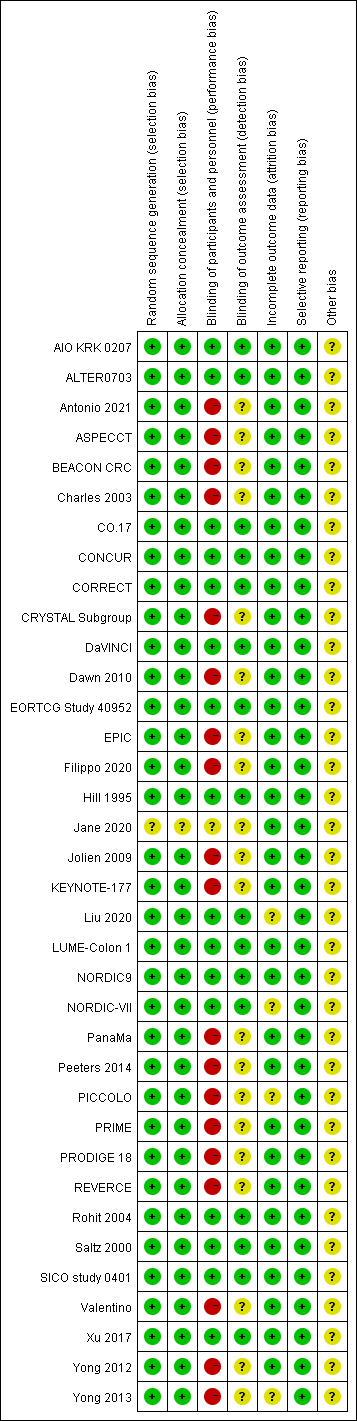


**Fig.S2 Risk of the Bias Assessment for Overall Network**


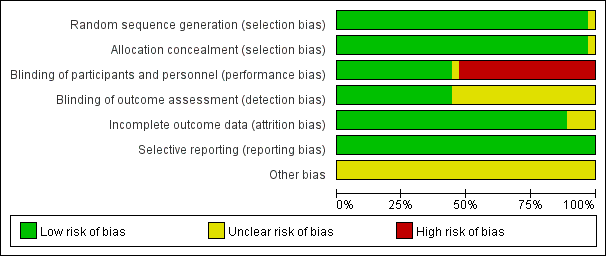


# **Supplementary File 6. Funnel Plots to Show Publication Bias**

**Fig.S1 Long-term, overall patients**


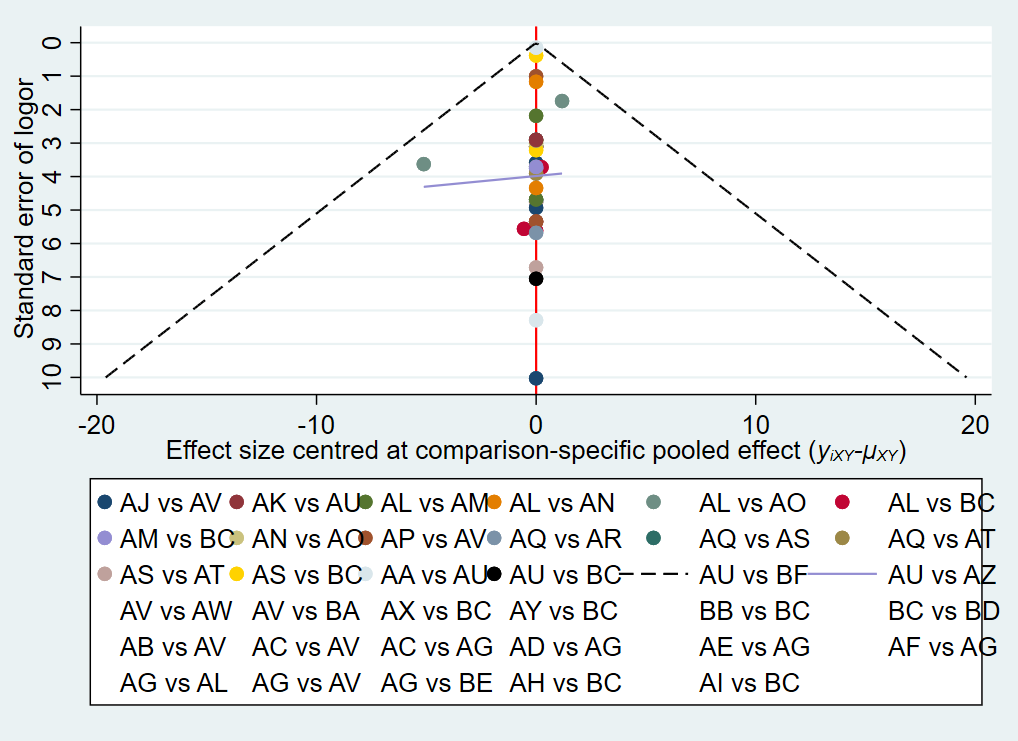


**Fig.S2 Long-term, first-line patients**


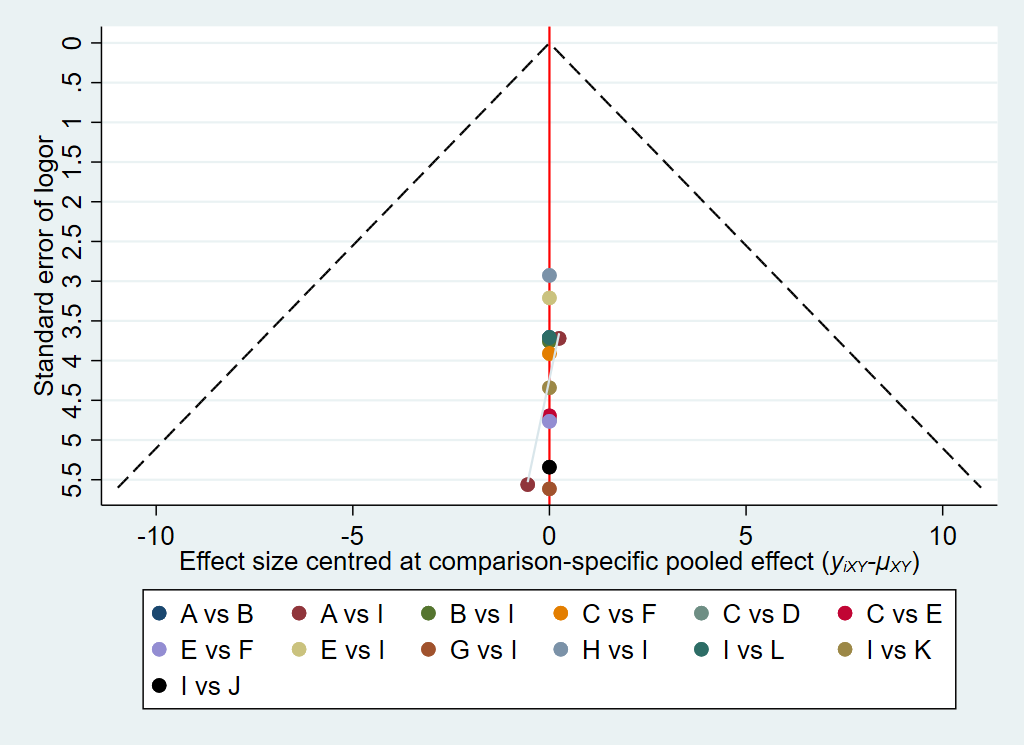


**Fig.S3 Long-term, subsequent-line patients**


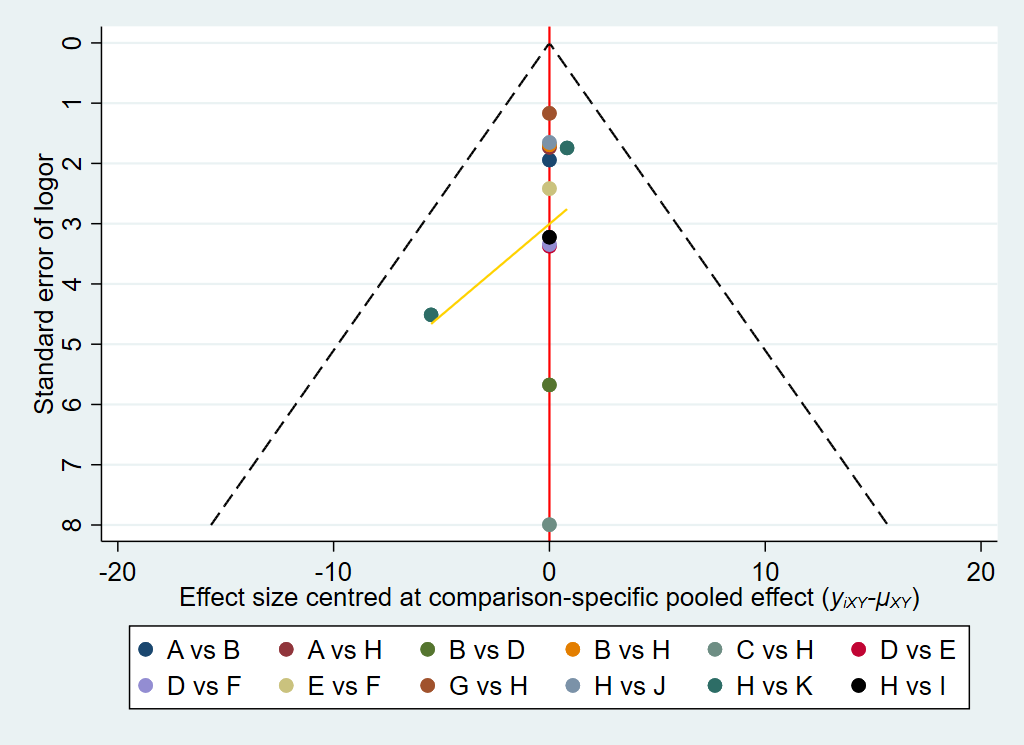


**Fig.S4 Short-term, overall patients**


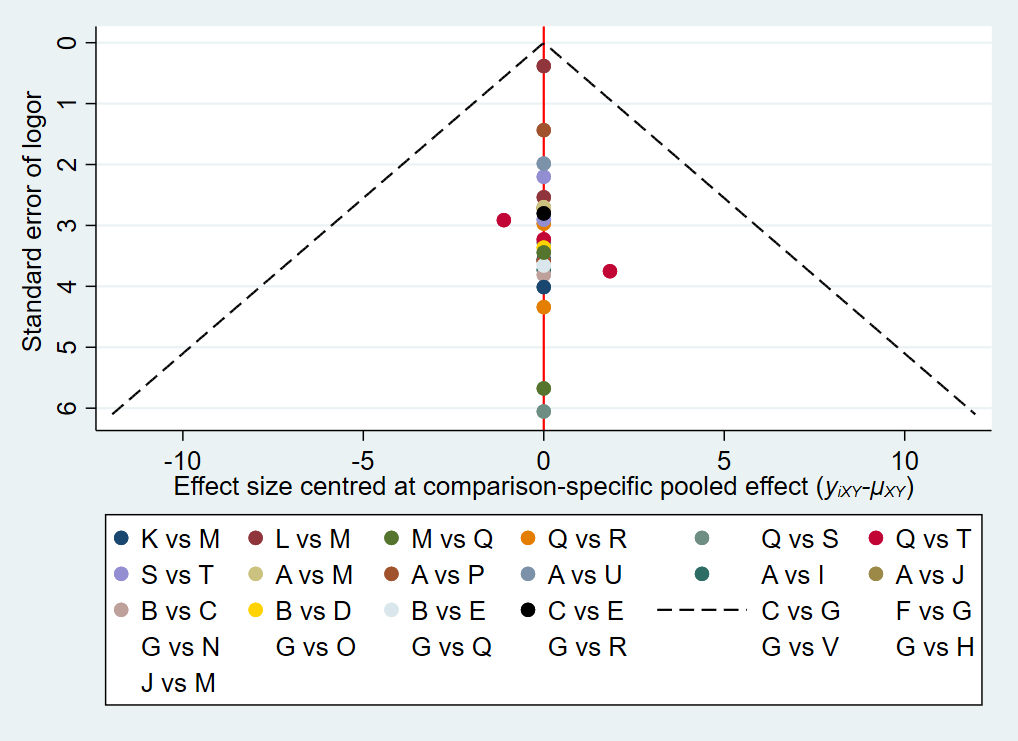


**Fig.S5 Short-term, first-line patients**


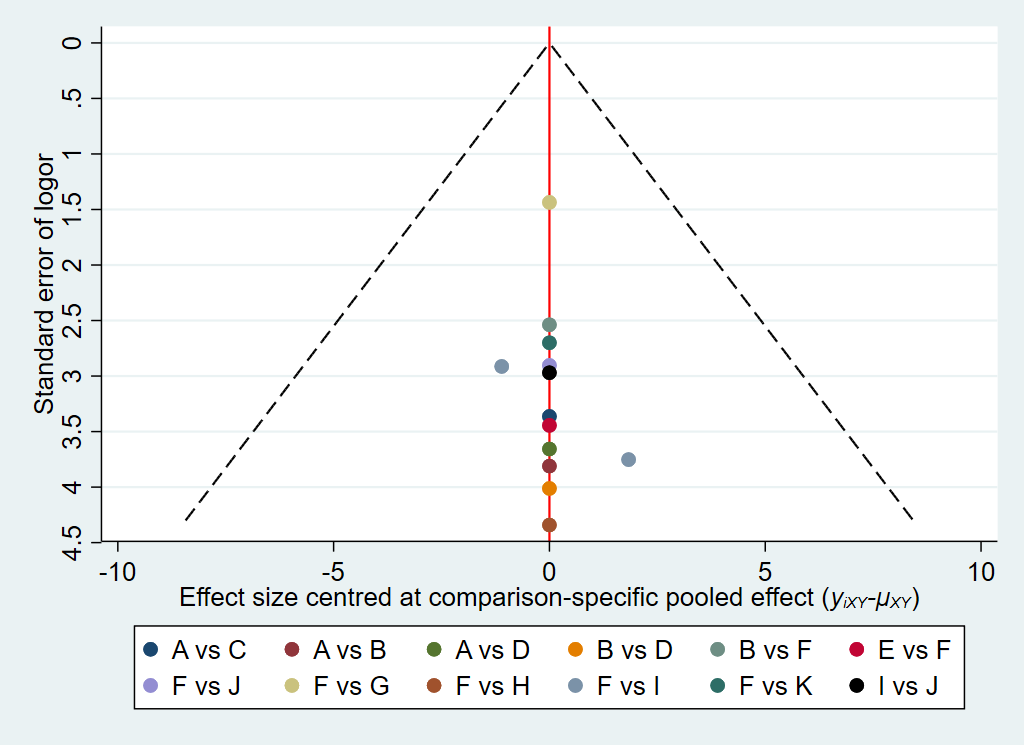


**Fig.S6 Short-term, subsequent-line patients**


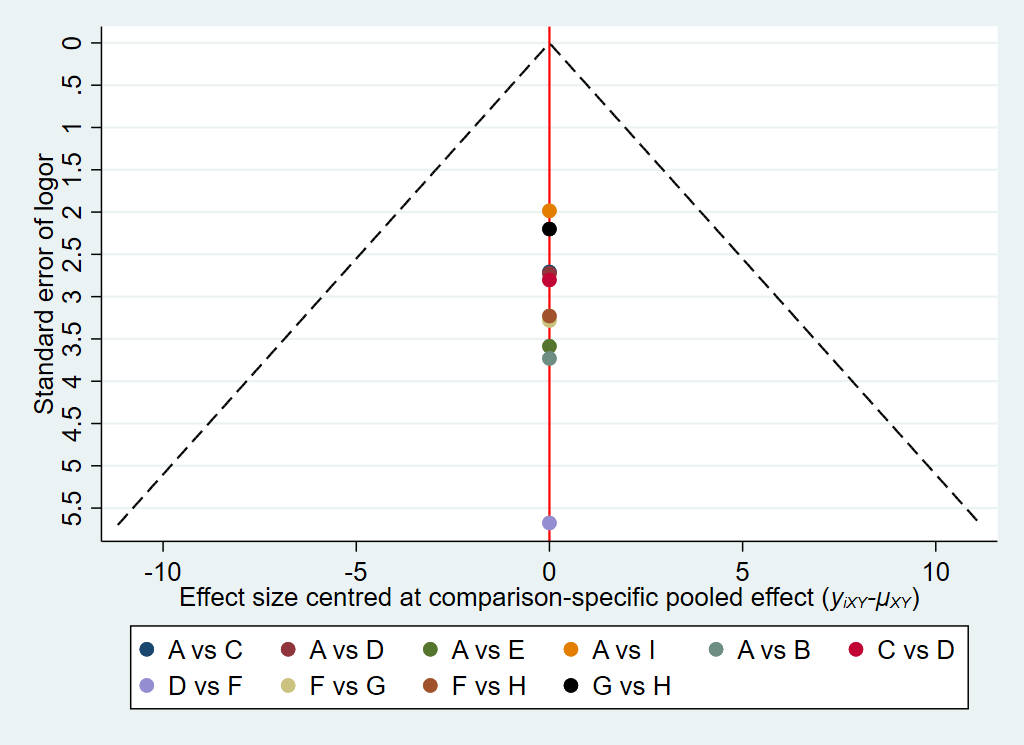


**Fig.S7 EQ-5D**


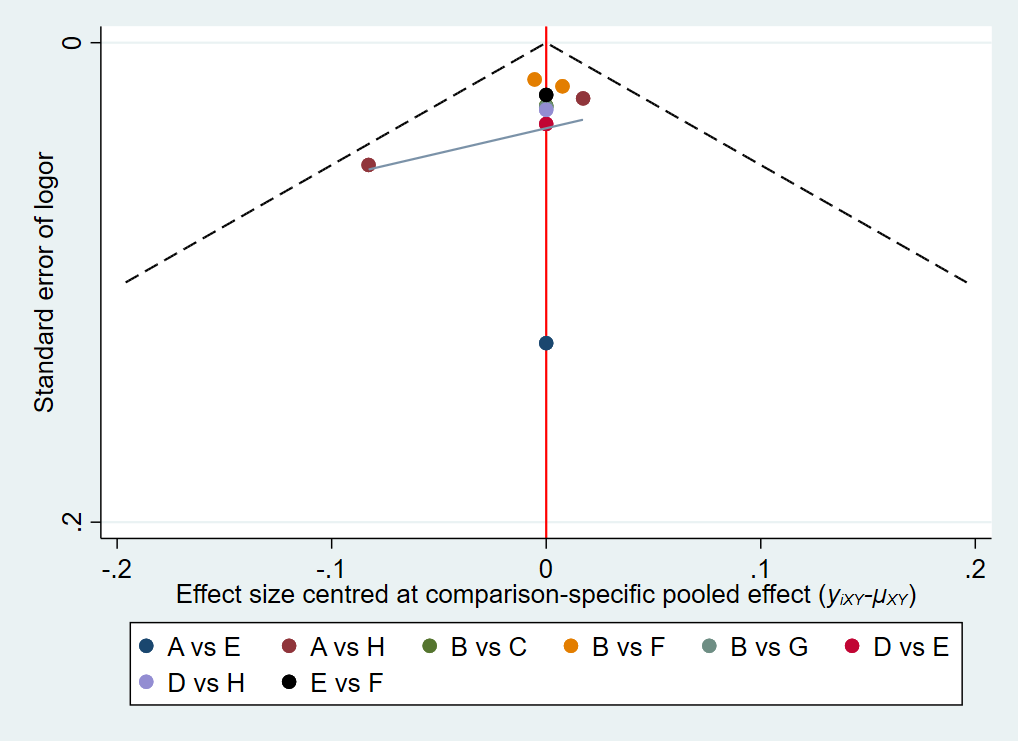


# **Supplementary File 7. League Tables and Network Plots for Subgroup Analysis**

Abbreviation ANL: anlotinib; BEV: bevacizumab; CAP, capecitabine; CET: cetuximab; inter Che: intermittent chemotherapy; Che: chemotherapy; INT: interferon; ENC: encorafenib; BIN: binimetinib; FAM: famitinib; FU, fluorouracil; ICI: Immunotherapy; IRI: Irinotecan; LV, leucovorin; NAT: No active treatment; NIN: nintedanib; Target: Target therapy; TCM: traditional Chinese medicine; PAN: panitumumab; PEM: pembrolizumab; REG: regorafenib; SIRT: Selective Internal Radiation Therapy

*:The asterisk (*) symbol used in the file denotes statistical significance. Specifically, we have used it to highlight results where the differences were found to be statistically significant. Conventionally, this indicates that the p-value for the associated test was below the predetermined threshold for significance, set at 0.05.

Note: Red coloration signifies that the treatment under consideration shows superiority over the control. Conversely, green indicates inferiority of the treatment relative to the control. White is used to denote that the effects between the treatment and control groups are relatively similar. Additionally, the intensity of the color correlates with the magnitude of the effect difference or the level of statistical significance; the deeper the color, the more pronounced the treatment effect or the higher the statistical significance

QLQ-C30: Regarding the range of the GHS/QoL subscale from the EORTC QLQ-C30 questionnaire, it typically ranges from 0 to 100. A high value on the GHS/QoL scale is considered indicative of good global health status and higher quality of life, whereas a low score would suggest poorer health and quality of life issues. As for the minimal clinically important difference for the GHS/QoL, a difference of 5 to 10 points on the GHS/QoL scale is often considered as a minimal clinically significant change

SUCRA：SUCRA values are interpreted as indicating the relative ranking of treatments. These values, which range from 0 to 100%, reflect the likelihood of each treatment being the most effective option within the network, with higher values suggesting better relative performance

**Fig.S1 Long-term Overall Analysis for First-line and Subsequent-line Patients (Left, First-line; Right, Subsequent-line. QLQ-C30, Without Differentiating Chemotherapy)**


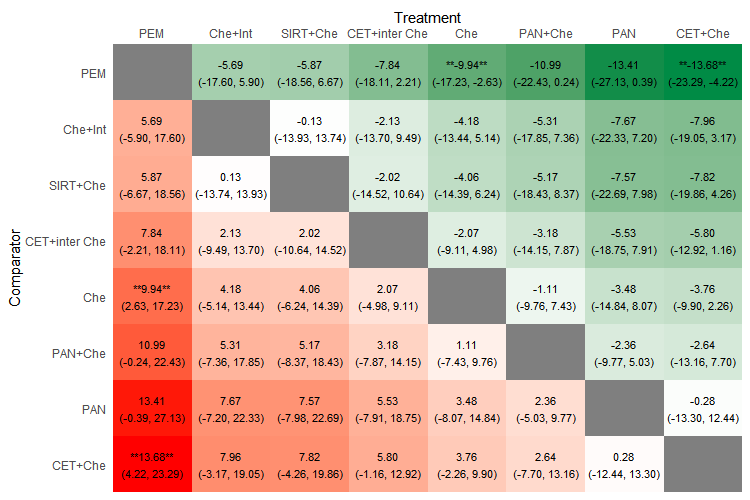

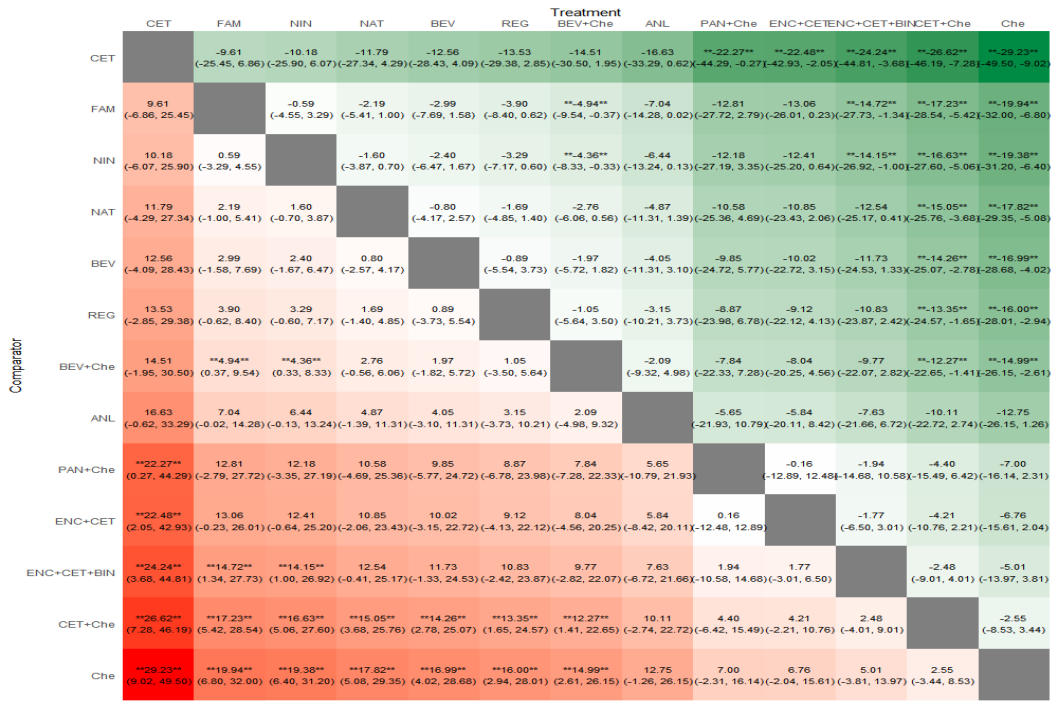


**Fig.S2 Long-term Overall Analysis for All Patients (QLQ-C30, Without Differentiating Chemotherapy)**


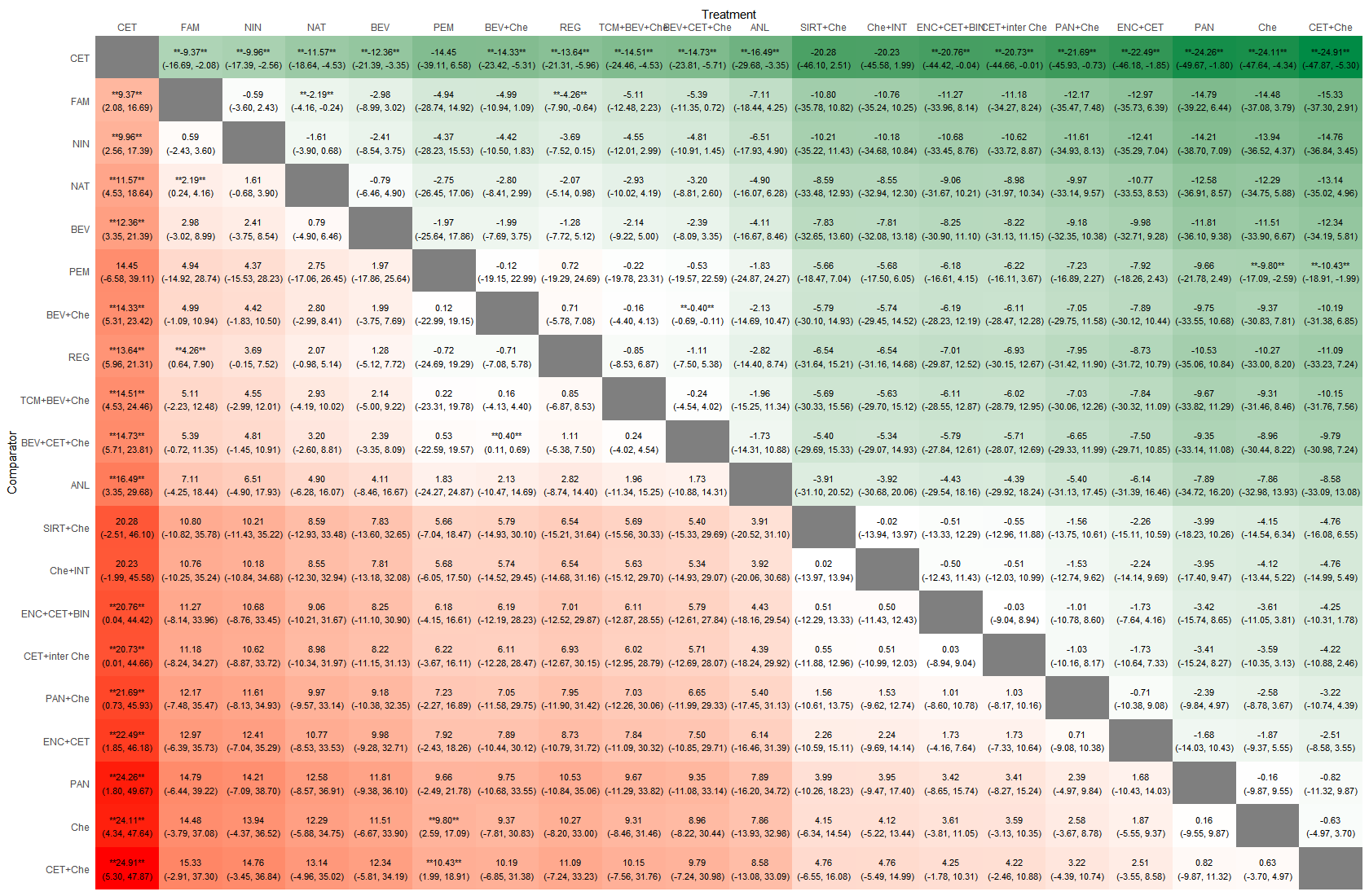


**Fig.S3 Short-term Overall Analysis For First-line and Subsequent-line Patients (A, First-line; B, Subsequent-line. QLQ-C30, Without Differentiating Chemotherapy)**


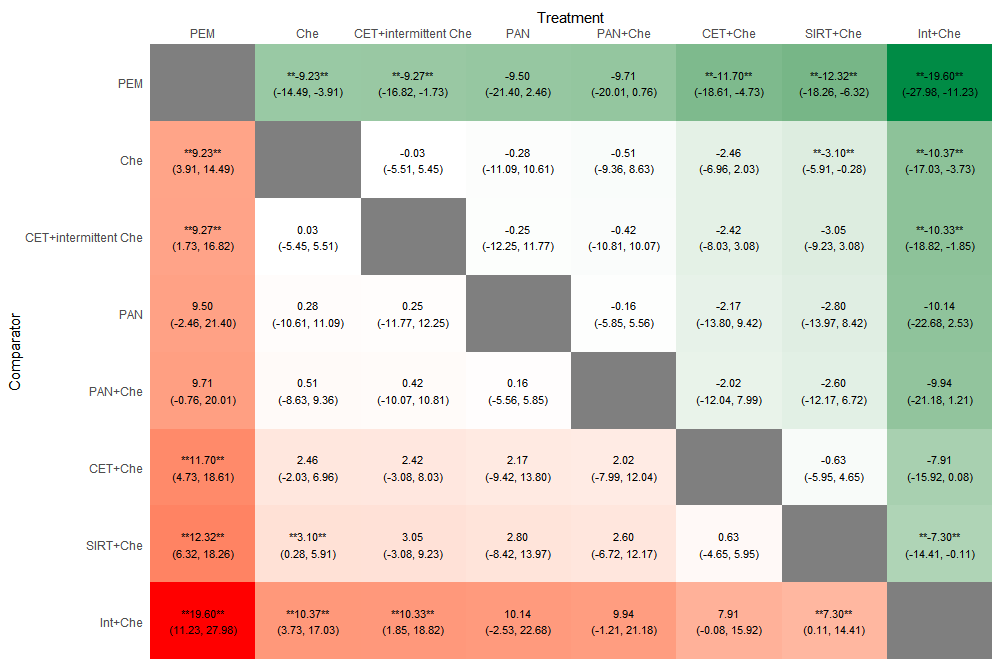

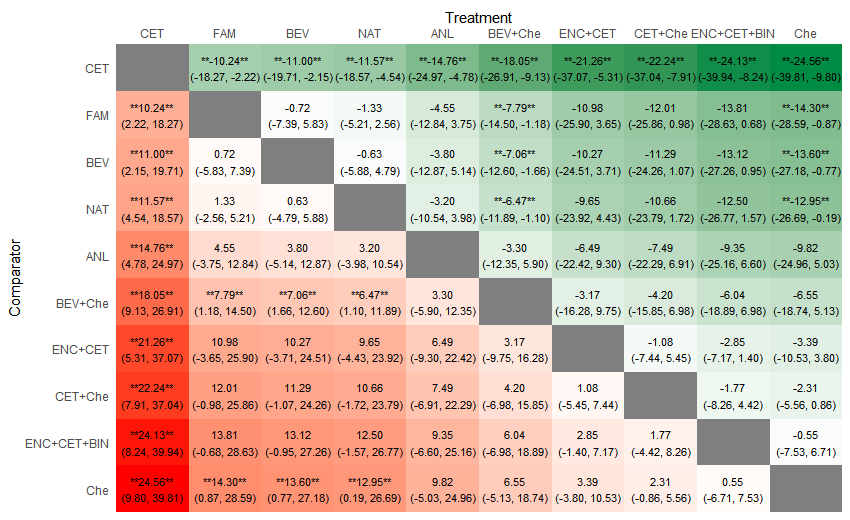


**Fig.S4 Short-term Overall Analysis for All Patients (QLQ-C30, Without Differentiating Chemotherapy)**


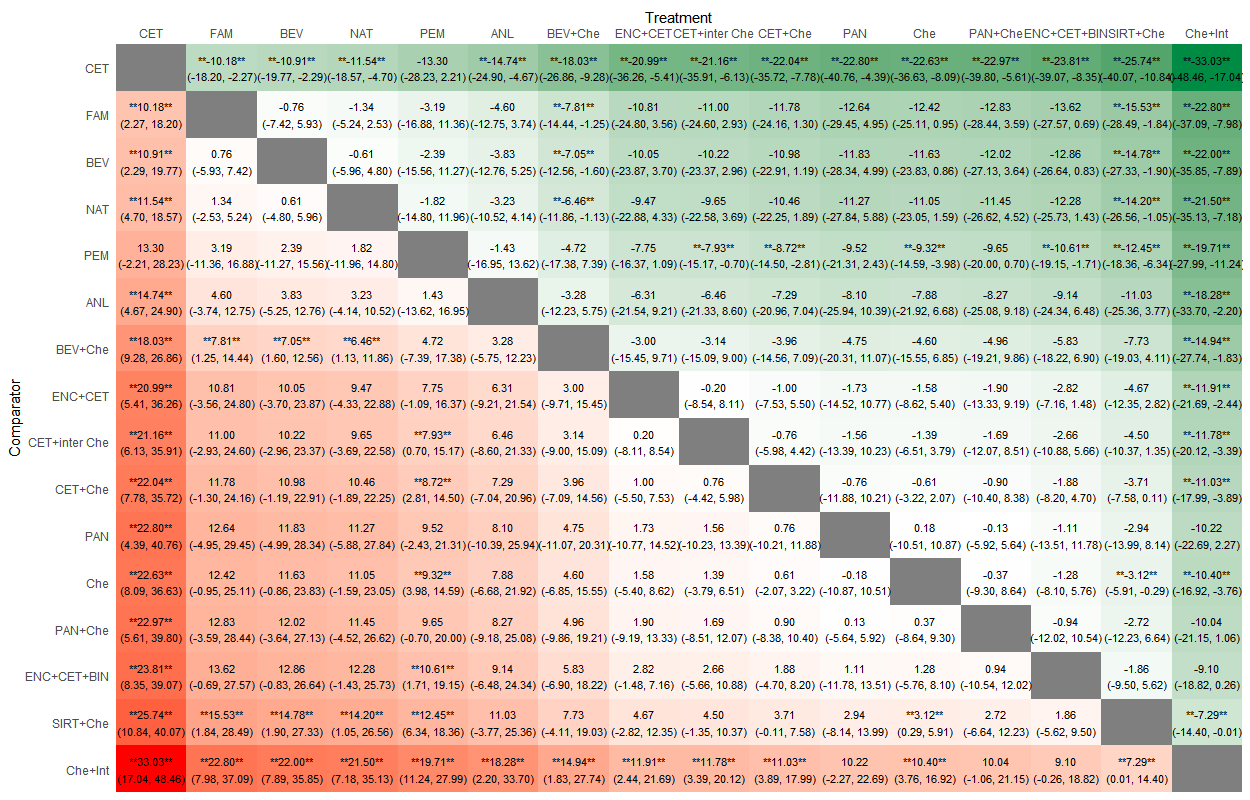


**Fig.S5 Comparison of Different Therapeutic Modalitiess (QLQ-C30)**


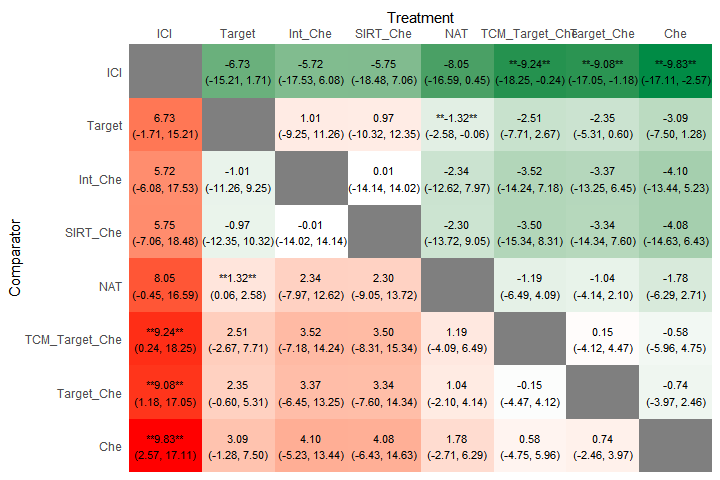


Abbreviation ANL: anlotinib; BEV: bevacizumab; CAP, capecitabine; CET: cetuximab; inter Che: intermittent chemotherapy; Che: chemotherapy; INT: interferon; ENC: encorafenib; BIN: binimetinib; FAM: famitinib; FU, fluorouracil; ICI: Immunotherapy; IRI: Irinotecan; LV, leucovorin; NAT: No active treatment; NIN: nintedanib; Target: Target therapy; TCM: traditional Chinese medicine; PAN: panitumumab; PEM: pembrolizumab; REG: regorafenib; SIRT: Selective Internal Radiation Therapy

QLQ-C30: Regarding the range of the GHS/QoL subscale from the EORTC QLQ-C30 questionnaire, it typically ranges from 0 to 100. A high value on the GHS/QoL scale is considered indicative of good global health status and higher quality of life, whereas a low score would suggest poorer health and quality of life issues. As for the minimal clinically important difference for the GHS/QoL, a difference of 5 to 10 points on the GHS/QoL scale is often considered as a minimal clinically significant change

Note: The bottom right corner includes any evidence, while the top left corner contains only direct evidence.

**Table. S1 Long-term Overall Analysis for First-line Patients (QLQ-C30, Without Differentiating Chemotherapy)**


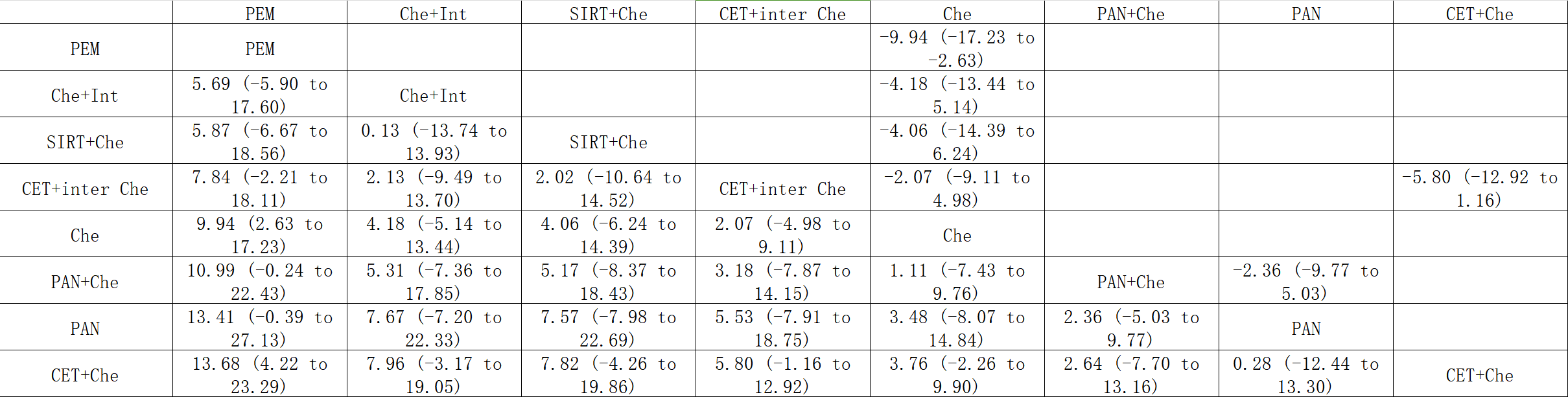


**Table. S2 Long-term Overall Analysis for Subsequent-line Patients (QLQ-C30, Without Differentiating Chemotherapy)**


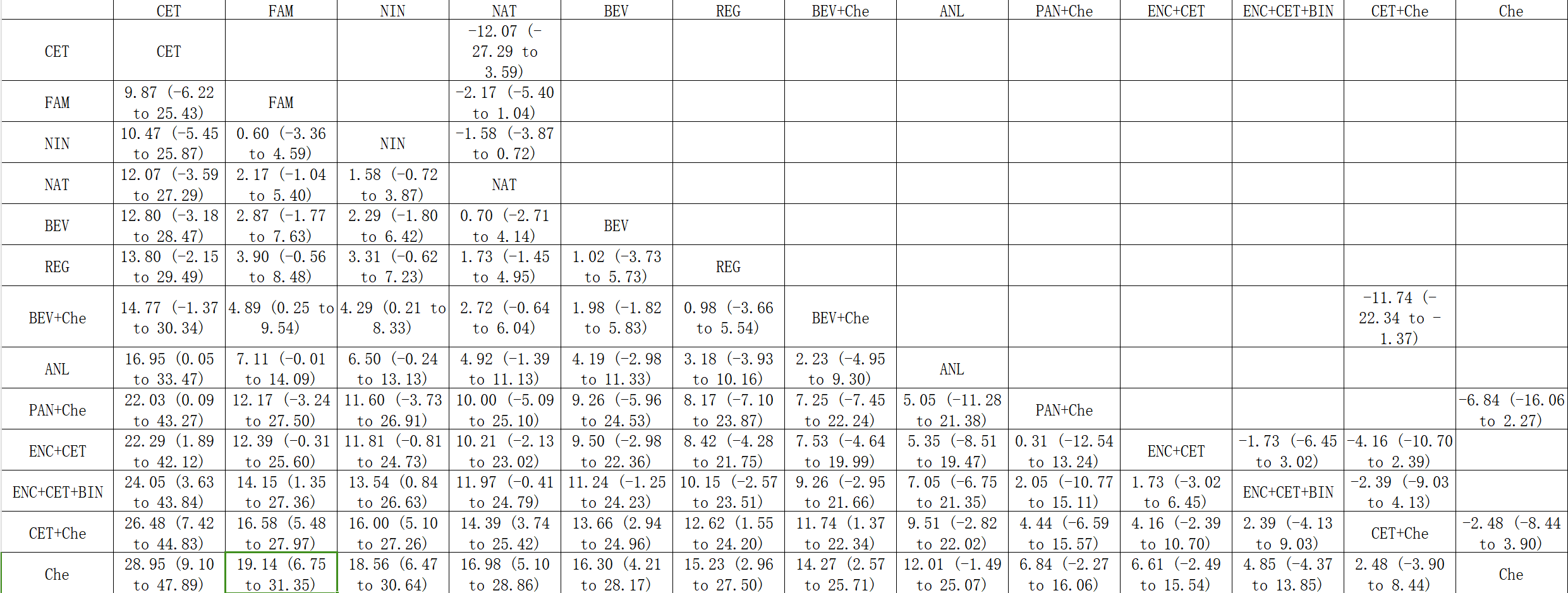


**Table. S3 Long-term Overall Analysis for Overall Patients (QLQ-C30, Without Differentiating Chemotherapy)**


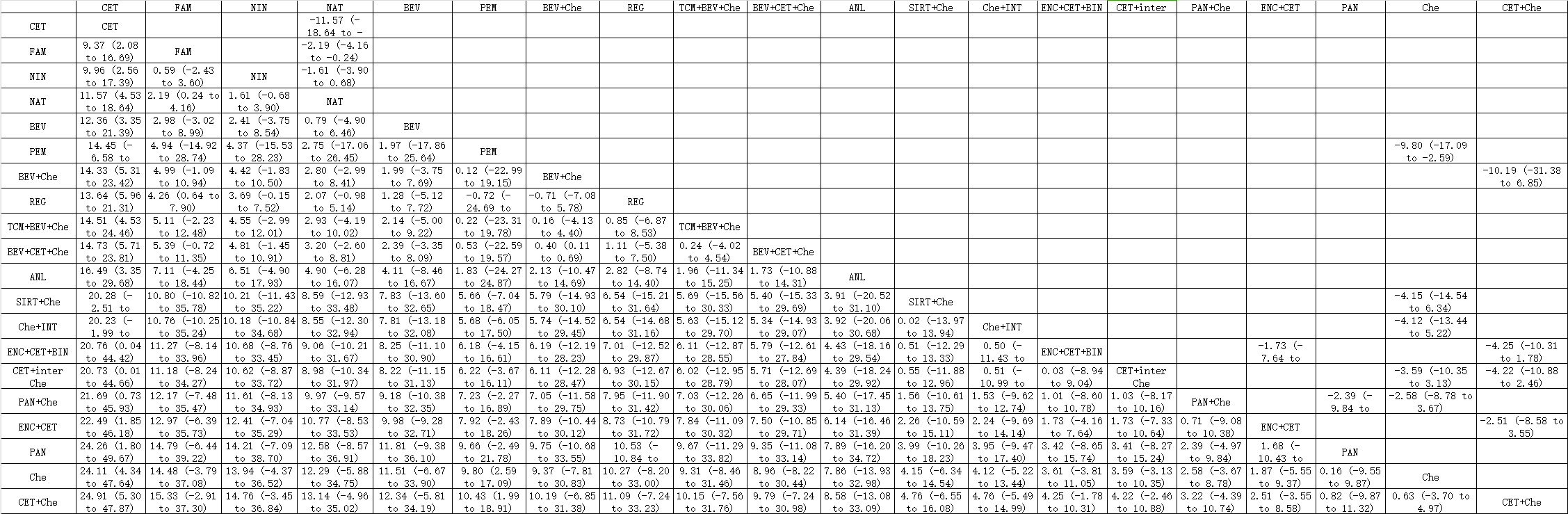


**Table. S4 Short-term Overall Analysis for First-line and Subsequent-line Patients (Above, First-line; Below, Subsequent-line. QLQ-C30, Without Differentiating Chemotherapy)**


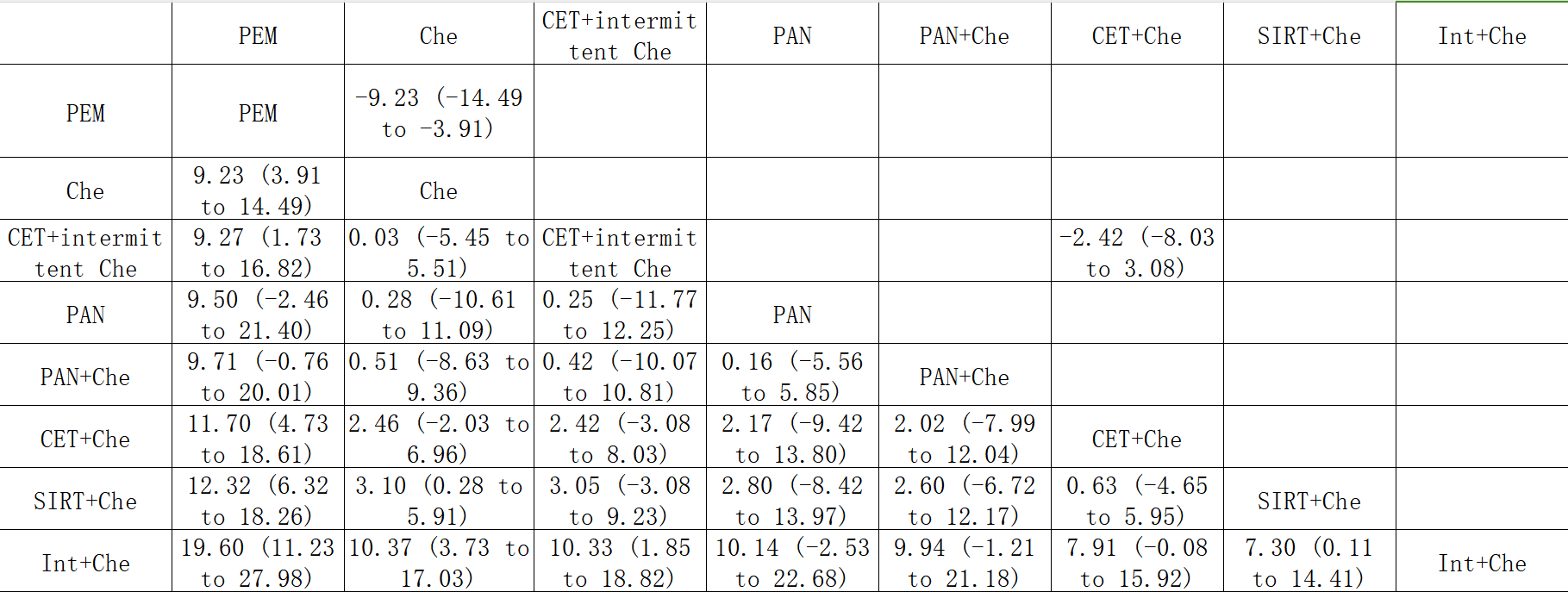


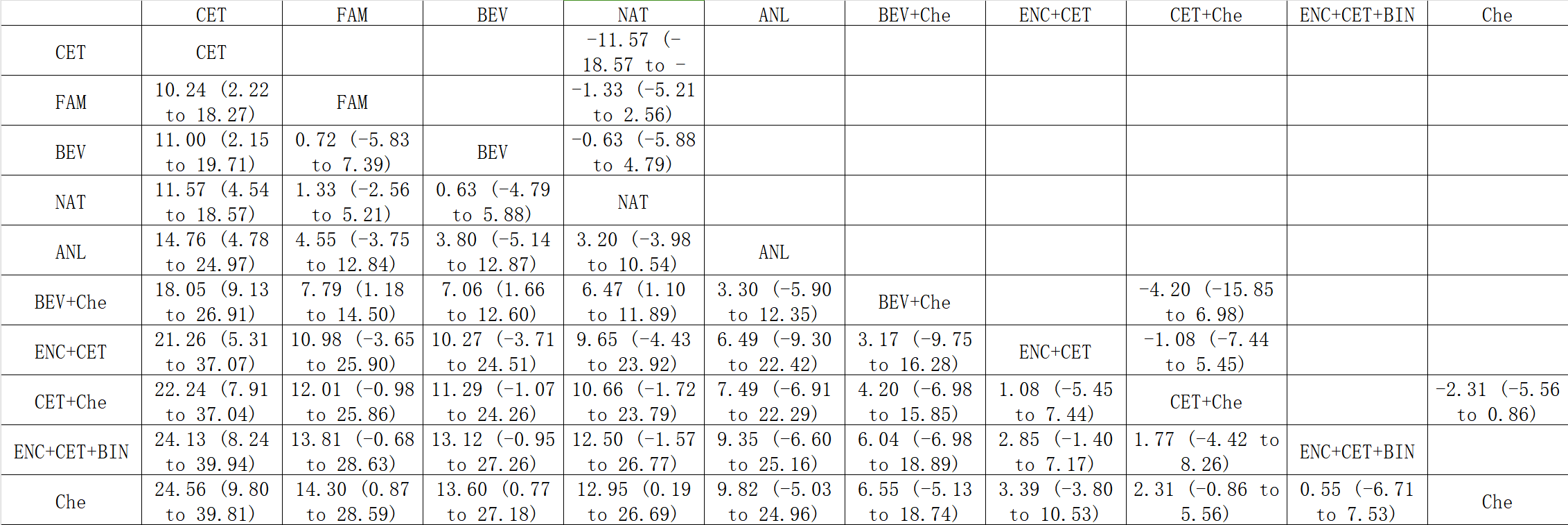


**Table. S5 Short-term Overall Analysis for Overall Patients (QLQ-C30, without Differentiating Chemotherapy)**


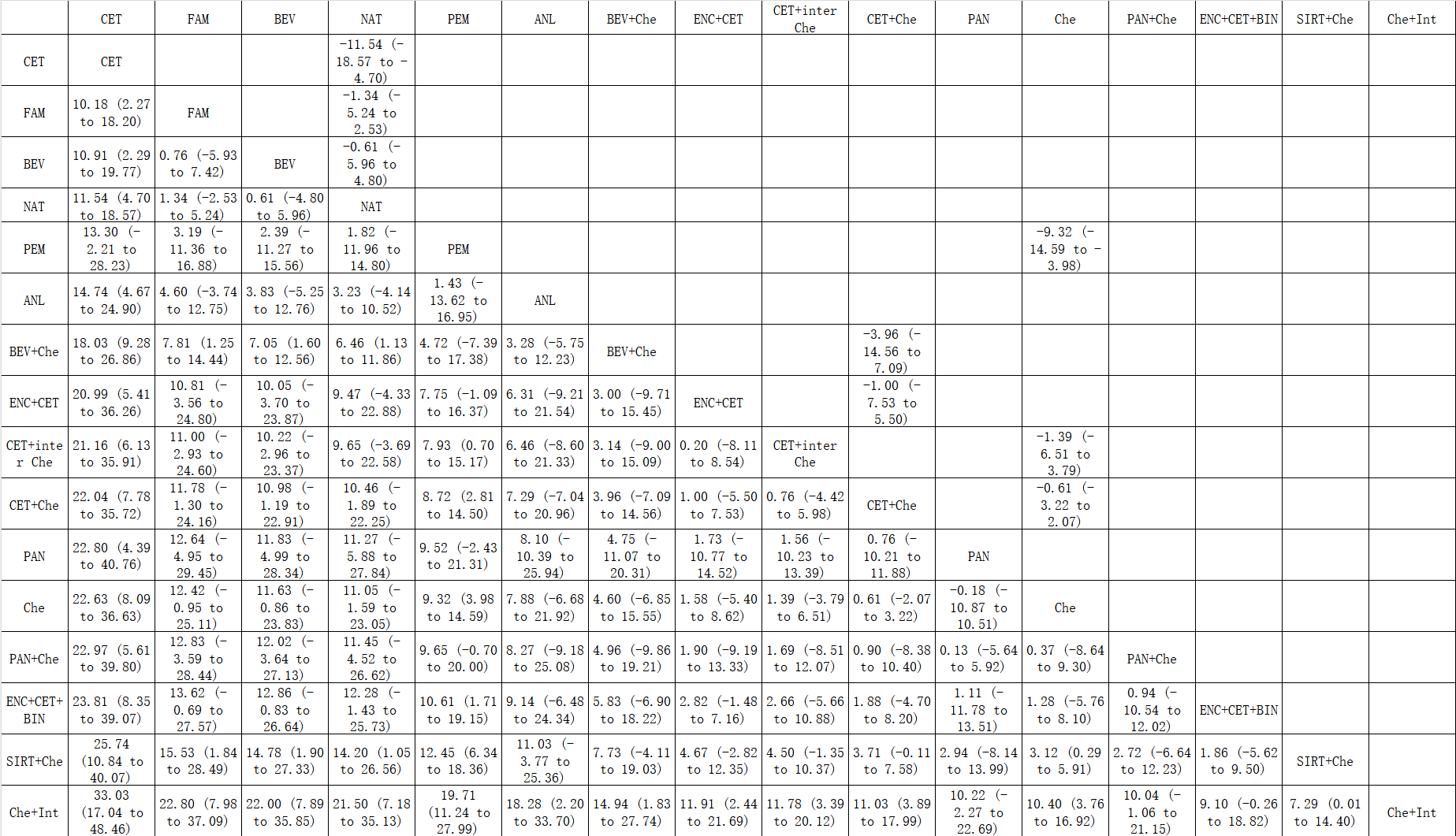


**Fig.S6 Network Plots for Subgroups in Overall Analysis (Without Differentiating Chemotherapy. A. Long-term First-line; B. Long-term Subsequent-line; C. Short-term First-line; D. Short-term Subsequent-line).**

Abbreviation ANL: anlotinib; BEV: bevacizumab; CAP, capecitabine; CET: cetuximab; inter Che: intermittent chemotherapy; Che: chemotherapy; INT: interferon; ENC: encorafenib; BIN: binimetinib; FAM: famitinib; FU, fluorouracil; ICI: Immunotherapy; IRI: Irinotecan; LV, leucovorin; NAT: No active treatment; NIN: nintedanib; Target: Target therapy; TCM: traditional Chinese medicine; PAN: panitumumab; PEM: pembrolizumab; REG: regorafenib; SIRT: Selective Internal Radiation Therapy

| **A** | 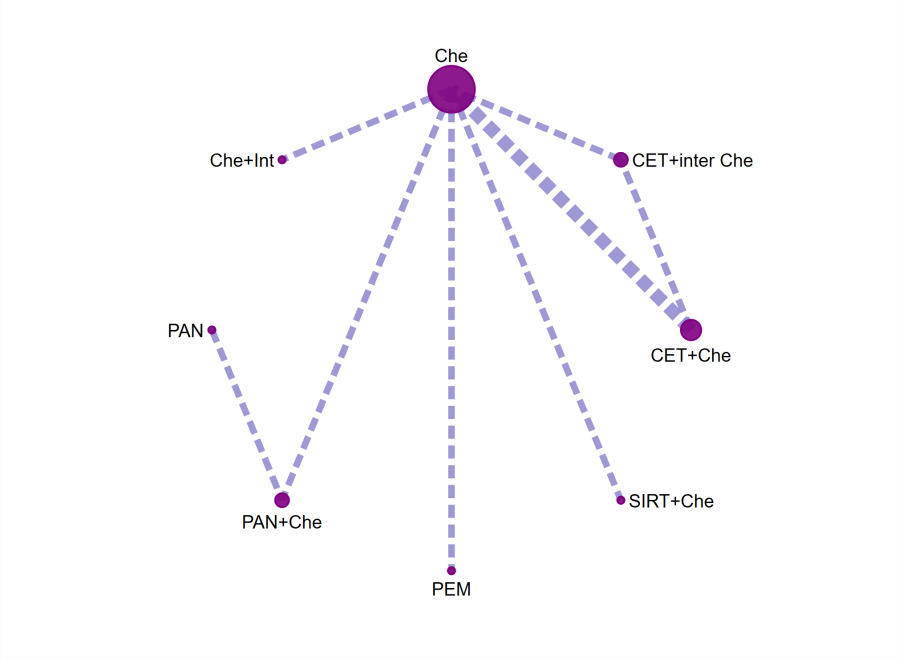 | **B** | 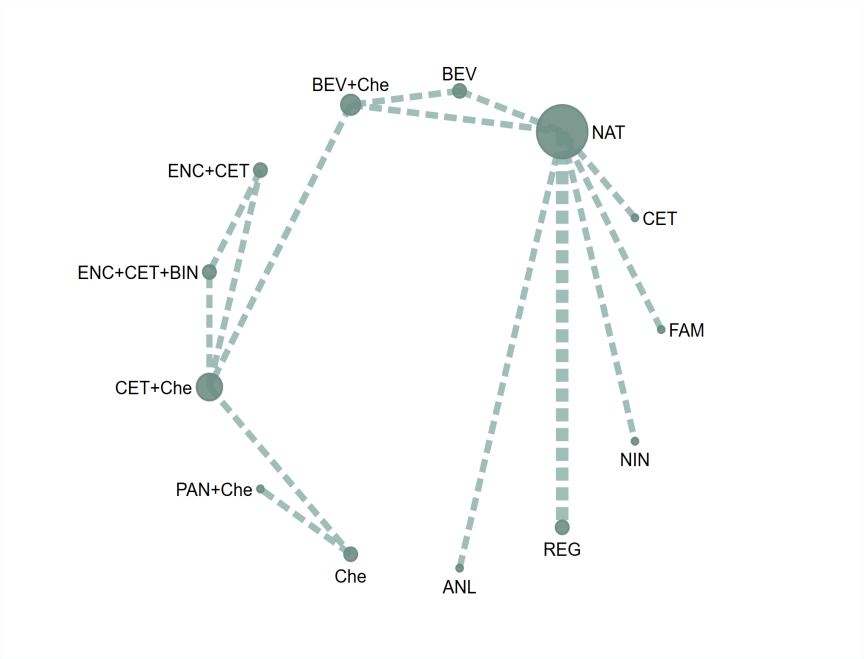 |
| --- | --- | --- | --- |
| **C** | 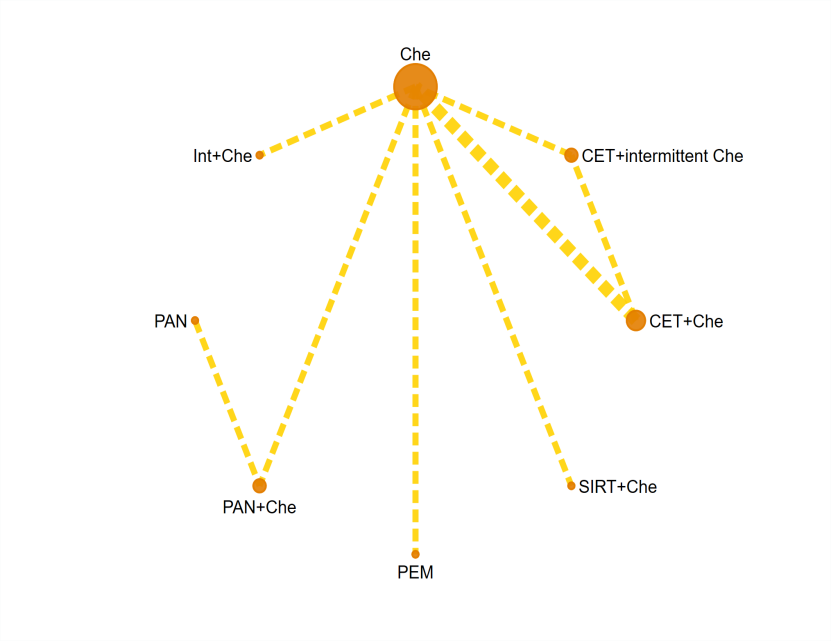 | **D** | 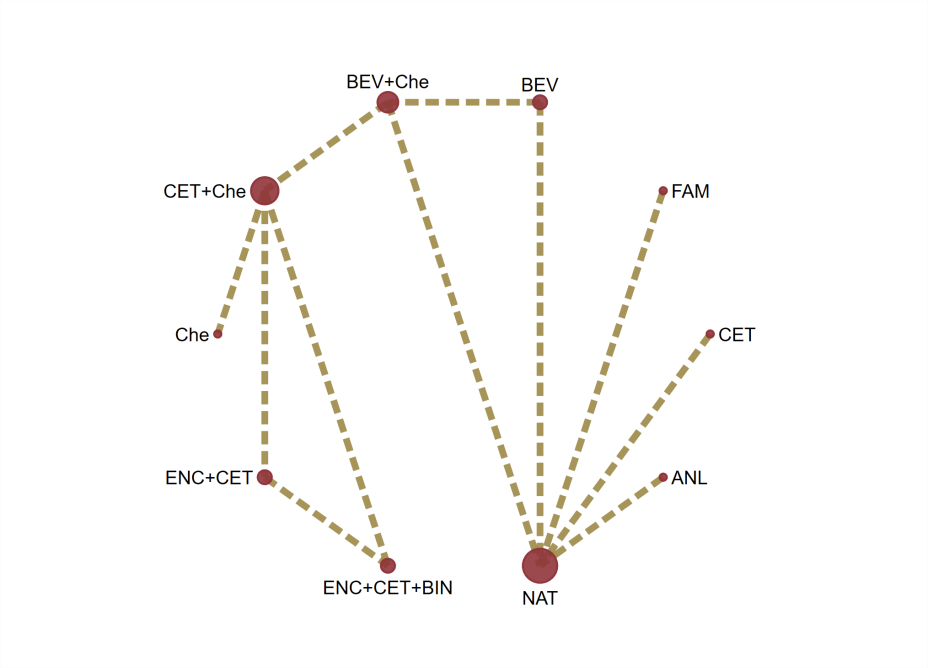 |

# **Supplementary File 8 Summarized Result of Comparison of Monotherapy, Combination Therapy and No Active Treatment**

**Table S1 Pooled Mean Differences (95% Credible Intervals) and SUCRA**

| SUCRA (%) | Mean Difference (95% CI) | 96.36 | 16.43 | 37.21 |
| --- | --- | --- | --- | --- |
|  |  | Long-term effect | | |
| 99.38 | Short-term effect | monotherapy | -2.17 (-4.78 to 0.44) | **-1.31 (-2.59 to -0.05)** |
| 12.27 |  | **5.71 (0.78 to 10.63)** | combination therapy | 0.85 (-1.97 to 3.68) |
| 38.25 |  | **3.70 (1.41 to 6.01)** | -2.02 (-7.43 to 3.44) | no active treatment |

**Fig S1 Network Plots (A Long-term; B Short-term)**


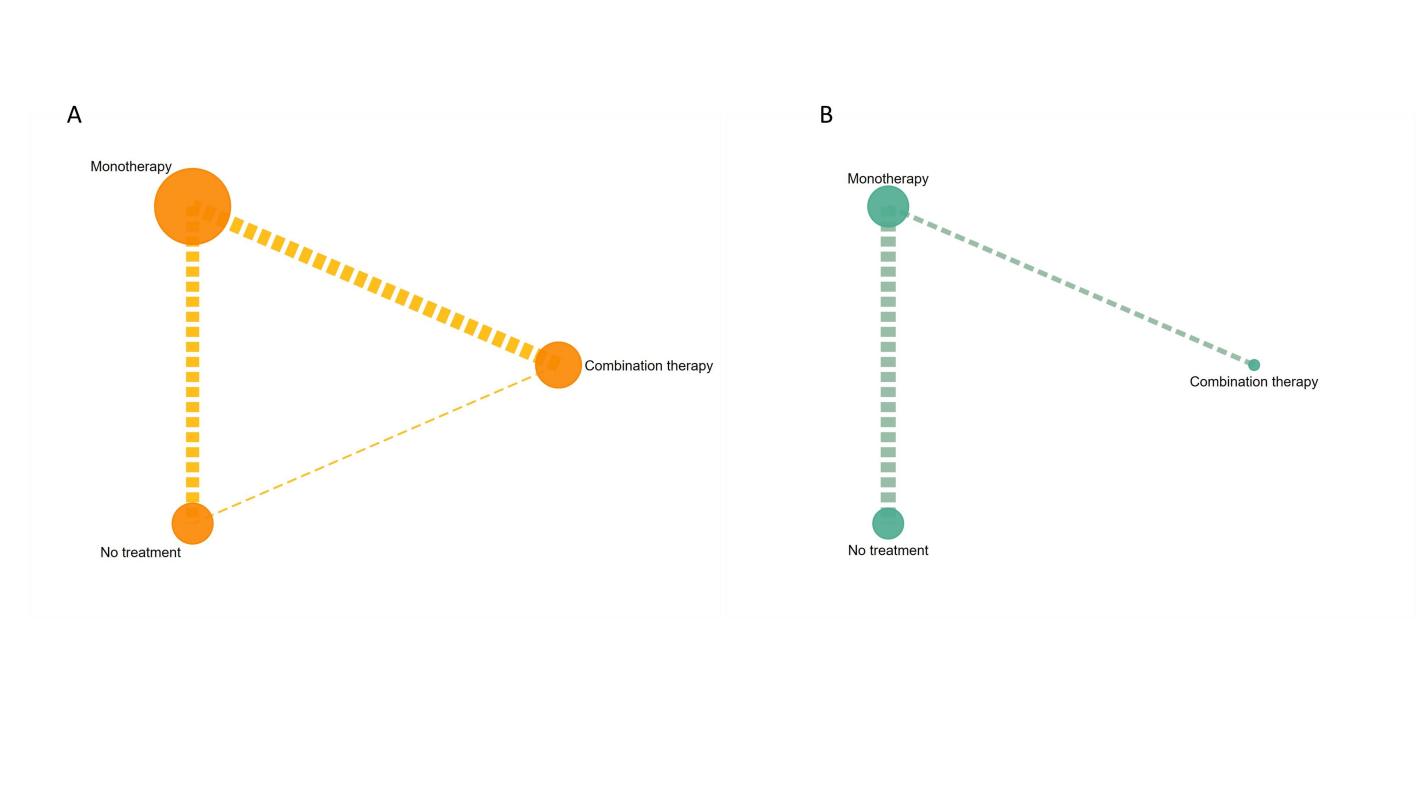


# **Supplementary File 9: Summarized Result of EQ-5D**

**Fig S1 Network Plots (A Long-term; B Short-term)**

| A | 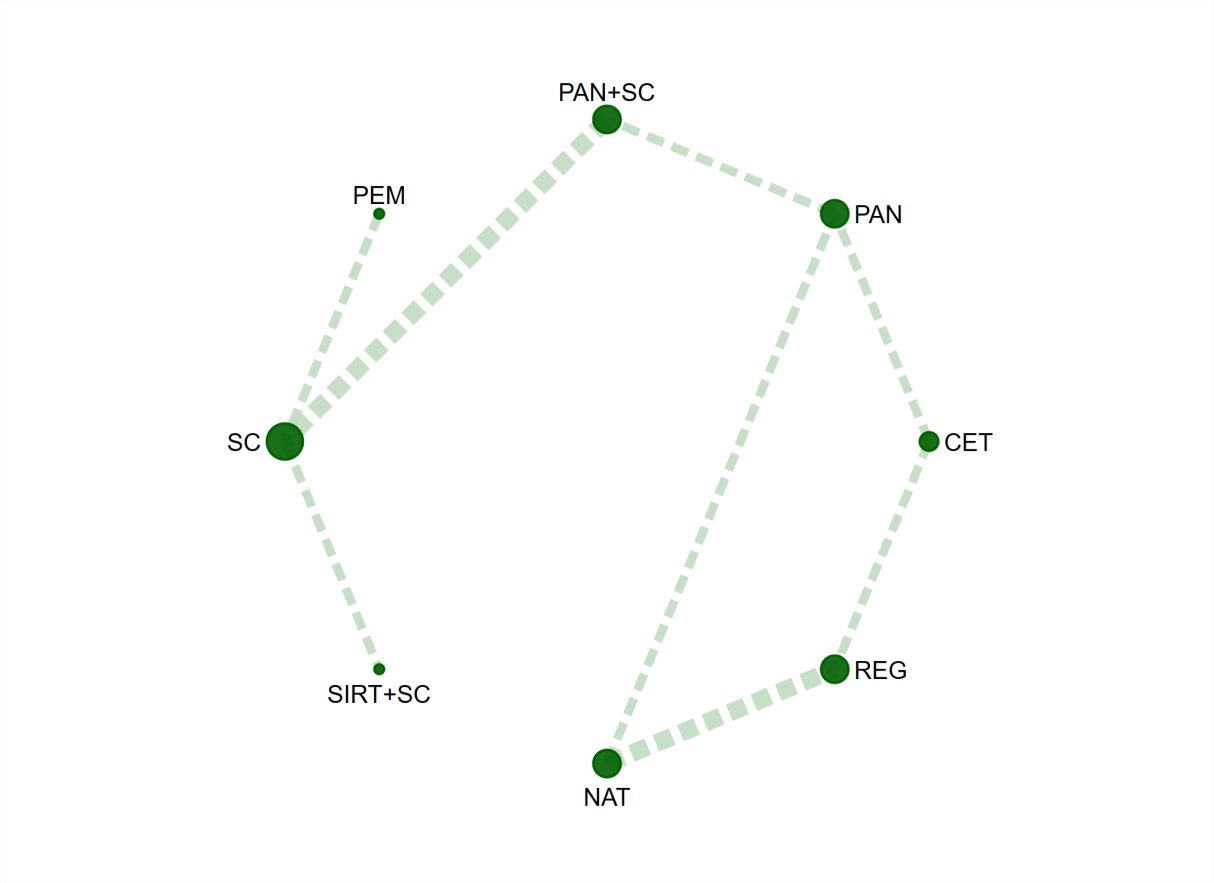 |
| --- | --- |
| B | 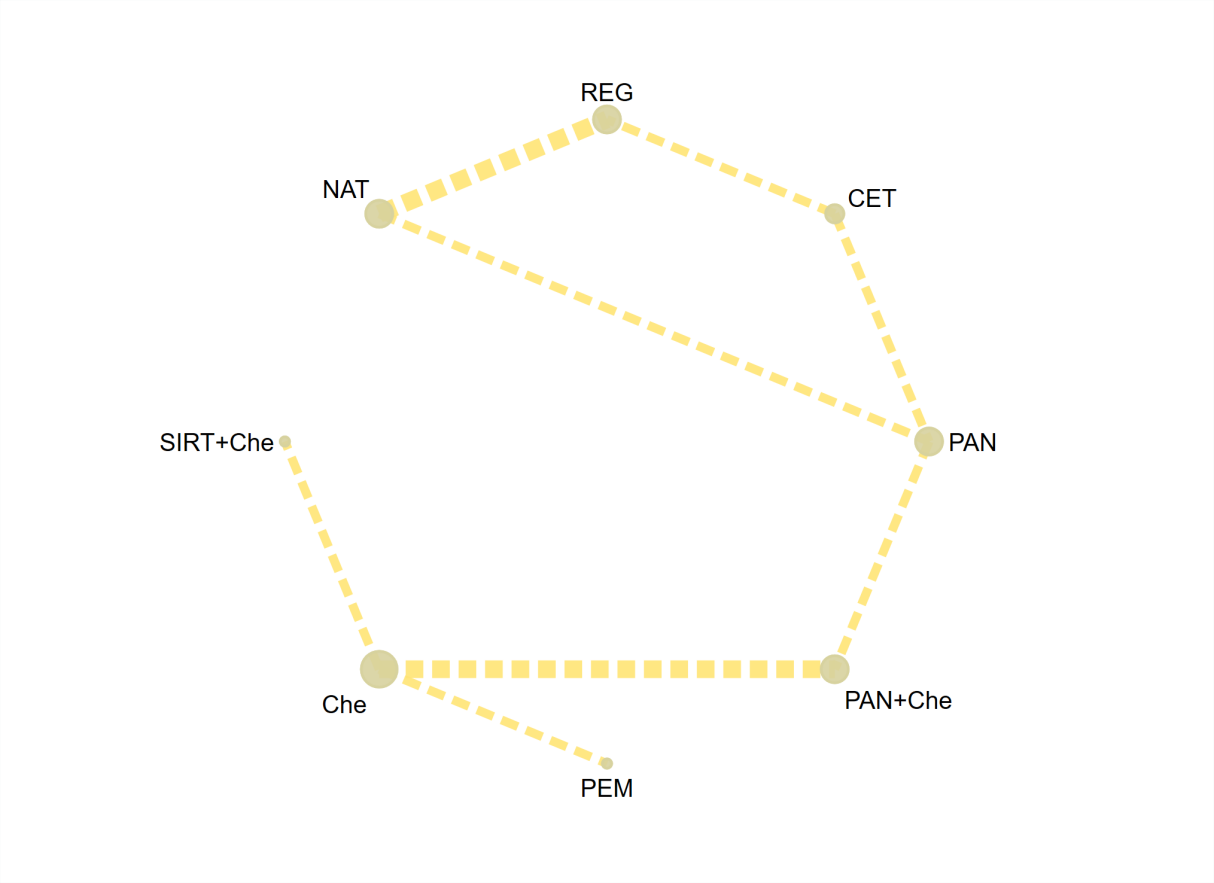 |

**Fig S2 League Tables (A Without Differentiating Chemotherapy; B Differentiate Chemotherapy)**

| A | 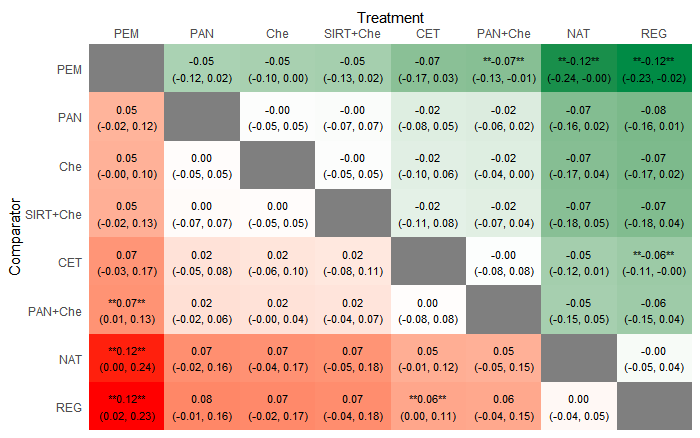 |
| --- | --- |
| B | 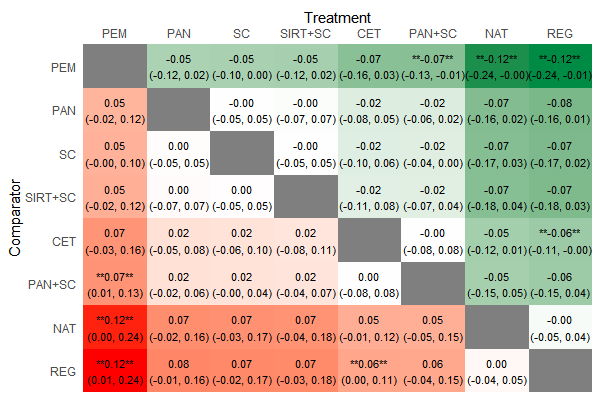 |

Abbreviation ANL: anlotinib; BEV: bevacizumab; BEV+Che:bevacizumab plus chemotherapy;CET: cetuximab; CET+Che: cetuximab plus chemotherapy; CET+inter Che: cetuximab plus intermittent chemotherapy; CET+BEV+Che: cetuximab combined with bevacizumab plus chemotherapy; Che: chemotherapy; Che+INT: interferon plus chemotherapy; ENC+CET: encorafenib plus cetuximab; ENC+CET+BIN: encorafenib combined with cetuximab plus binimetinib; FAM: famitinib; ICI: Immunotherapy; NAT: No active treatment; NIN: nintedanib; Target: Target therapy; Target+Che:Target therapy plus chemotherapy; TCM+BEV+Che: traditional Chinese medicine combined with bevacizumab plus chemotherapy; PAN: panitumumab; PAN+Che: panitumumab plus chemotherapy; PEM: pembrolizumab; REG: regorafenib; SIRT+Che: SIRT plus chemotherapy.

*:The asterisk (*) symbol used in the file denotes statistical significance. Specifically, we have used it to highlight results where the differences were found to be statistically significant. Conventionally, this indicates that the p-value for the associated test was below the predetermined threshold for significance, set at 0.05.

Note: Red coloration signifies that the treatment under consideration shows superiority over the control. Conversely, green indicates inferiority of the treatment relative to the control. White is used to denote that the effects between the treatment and control groups are relatively similar. Additionally, the intensity of the color correlates with the magnitude of the effect difference or the level of statistical significance; the deeper the color, the more pronounced the treatment effect or the higher the statistical significance

The EQ-5D scores health across five dimensions and provides a single summary index value. Each dimension has three response levels indicating no, some, or extreme problems, resulting in a health profile. The index value is derived from these profiles using a formula that assigns weights to each level; the possible values range from less than 0 (representing states worse than death) to 1 (full health), with some country-specific value sets allowing for values above 1.

# **Supplementary File 10 Heterogeneity Assessment Results**

| Network | Data points | Ratios^*^ | I^2^ |
| --- | --- | --- | --- |
| Long-term QLQ-C30 GHS/QOL for overall patients (No distinction for chemotherapy) | 43 | 1.055 | 7% |
| Long-term QLQ-C30 GHS/QOL for patients received first-line treatments (No distinction for chemotherapy) | 15 | 0.965 | 3% |
| Long-term QLQ-C30 GHS/QOL for patients received subsequent-line treatments (No distinction for chemotherapy) | 27 | 1.032 | 7% |
| Short-term QLQ-C30 GHS/QOL for overall patients (No distinction for chemotherapy) | 31 | 1.039 | 7% |
| Short-term QLQ-C30 GHS/QOL for patients received first-line treatments (No distinction for chemotherapy) | 15 | 0.969 | 4% |
| Short-term QLQ-C30 GHS/QOL for patients received subsequent-line treatments (No distinction for chemotherapy) | 16 | 1.003 | 3% |
| EQ-5D (No distinction for chemotherapy) | 20 | 1.059 | 10% |
| Long-term QLQ-C30 GHS/QOL for overall patients (Monotherapy VS Combination therapy VS No active treatment) | 29 | 1.089 | 11% |
| Short-term QLQ-C30 GHS/QOL for overall patients (Monotherapy VS Combination therapy VS No active treatment) | 16 | 1.874 | 50% |
| Long-term QLQ-C30 GHS/QOL for overall patients (therapeutic modalities comparison) | 41 | 1.261 | 23% |
| Short-term QLQ-C30 GHS/QOL for overall patients (therapeutic modalities comparison) | 29 | 1.301 | 26% |
| Long-term QLQ-C30 GHS/QOL for overall patients (Distinction for chemotherapy) | 64 | 1.008 | 2% |
| Long-term QLQ-C30 GHS/QOL for patients received first-line treatments (Distinction for chemotherapy) | 22 | 0.969 | 2% |
| Long-term QLQ-C30 GHS/QOL for patients received subsequent-line treatments (Distinction for chemotherapy) | 20/10 | 1.031/1.004 | 8%/10% |
| Short-term QLQ-C30 GHS/QOL for overall patients (Distinction for chemotherapy) | 40 | 0.988 | 1% |
| Short-term QLQ-C30 GHS/QOL for patients received first-line treatments (Distinction for chemotherapy) | 20 | 0.979 | 3% |
| Short-term QLQ-C30 GHS/QOL for patients received subsequent-line treatments (Distinction for chemotherapy) | 14 | 0.997 | 7% |
| EQ-5D (Distinction for chemotherapy) | 20 | 1.017 | 7% |
| Long-term QLQ-C30 GHS/QOL for overall patients (No distinction for chemotherapy, Sensitive analysis) | 38 | 1.062 | 8% |
| Long-term QLQ-C30 GHS/QOL for first-line patients (No distinction for chemotherapy, Sensitive analysis) | 13 | 0.941 | 2% |
| Long-term QLQ-C30 GHS/QOL for subsequent-line patients (No distinction for chemotherapy, Sensitive analysis) | 21 | 1.035 | 8% |

* 'Ratios' in this file denote the H statistic values, a measure of heterogeneity in meta-analysis. The H statistic compares observed variance to expected variance under homogeneity. Interpretation of H values is as follows: H < 1: Negligible heterogeneity; H = 1 to 1.2: Low heterogeneity; H = 1.2 to 1.5: Moderate heterogeneity; H > 1.5 to 2: Substantial heterogeneity; H > 2: Considerable heterogeneity.

# **Supplementary File 11 Evidence Level Assessment (A), Transitivity (B), Direct Evidence Contribution (C), Predictive Interval (D)**

**A . Evidence Level Assessment, Using CINeMA Approach**

Abbreviation ANL: anlotinib; BEV: bevacizumab; BEV+Che:bevacizumab plus chemotherapy;CET: cetuximab; CET+Che: cetuximab plus chemotherapy; CET+inter Che: cetuximab plus intermittent chemotherapy; CET+BEV+Che: cetuximab combined with bevacizumab plus chemotherapy; Che: chemotherapy; Che+INT: interferon plus chemotherapy; ENC+CET: encorafenib plus cetuximab; ENC+CET+BIN: encorafenib combined with cetuximab plus binimetinib; FAM: famitinib; ICI: Immunotherapy; NAT: No active treatment; NIN: nintedanib; Target: Target therapy; Target+Che:Target therapy plus chemotherapy; TCM+BEV+Che: traditional Chinese medicine combined with bevacizumab plus chemotherapy; PAN: panitumumab; PAN+Che: panitumumab plus chemotherapy; PEM: pembrolizumab; REG: regorafenib; SIRT+Che: SIRT plus chemotherapy.

1. **Comparison of Therapeutic Modalities (Long-term)**

| Comparison | Number of studies | Within-study bias | Reporting bias | Indirectness | Imprecision | Heterogeneity | Incoherence | Confidence rating |
| --- | --- | --- | --- | --- | --- | --- | --- | --- |
| Che:ICI | 1 | Some concerns | Some concerns | Some concerns | No concerns | Major concerns | No concerns | Low |
| Che:Int_Che | 1 | No concerns | Low risk | No concerns | Some concerns | No concerns | No concerns | Moderate |
| Che:SIRT_Che | 1 | Some concerns | Some concerns | No concerns | Some concerns | No concerns | No concerns | Low |
| Che:Target_Che | 6 | Some concerns | Some concerns | No concerns | Some concerns | No concerns | No concerns | Low |
| NAT:Target | 7 | No concerns | Low risk | No concerns | No concerns | Major concerns | No concerns | Moderate |
| NAT:Target_Che | 1 | No concerns | Low risk | No concerns | Some concerns | No concerns | No concerns | Moderate |
| Target_Che:TCM_Target_Che | 1 | No concerns | Low risk | No concerns | Some concerns | No concerns | No concerns | Moderate |
| Target:Target_Che | 4 | Some concerns | Some concerns | Some concerns | Some concerns | No concerns | No concerns | Low |
| Che:NAT | 0 | No concerns | Low risk | No concerns | Some concerns | No concerns | No concerns | Moderate |
| Che:TCM_Target_Che | 0 | No concerns | Low risk | No concerns | Some concerns | No concerns | No concerns | Moderate |
| Che:Target | 0 | Some concerns | Some concerns | No concerns | Some concerns | No concerns | No concerns | Low |
| ICI:Int_Che | 0 | No concerns | Low risk | No concerns | Major concerns | No concerns | No concerns | Moderate |
| ICI:NAT | 0 | Some concerns | Some concerns | No concerns | Some concerns | No concerns | No concerns | Low |
| ICI:SIRT_Che | 0 | Some concerns | Some concerns | No concerns | Some concerns | No concerns | No concerns | Low |
| ICI:TCM_Target_Che | 0 | Some concerns | Some concerns | No concerns | No concerns | Major concerns | No concerns | Low |
| ICI:Target | 0 | Some concerns | Some concerns | Some concerns | Major concerns | No concerns | No concerns | Low |
| ICI:Target_Che | 0 | Some concerns | Some concerns | Some concerns | No concerns | Major concerns | No concerns | Very Low |
| Int_Che:NAT | 0 | No concerns | Low risk | No concerns | Major concerns | No concerns | No concerns | Moderate |
| Int_Che:SIRT_Che | 0 | No concerns | Low risk | No concerns | Some concerns | No concerns | No concerns | Moderate |
| Int_Che:TCM_Target_Che | 0 | No concerns | Low risk | No concerns | Some concerns | No concerns | No concerns | Moderate |
| Int_Che:Target | 0 | No concerns | Low risk | No concerns | Some concerns | No concerns | No concerns | Moderate |
| Int_Che:Target_Che | 0 | No concerns | Low risk | No concerns | Some concerns | No concerns | No concerns | Moderate |
| NAT:SIRT_Che | 0 | Some concerns | Some concerns | No concerns | Some concerns | No concerns | No concerns | Low |
| NAT:TCM_Target_Che | 0 | No concerns | Low risk | No concerns | Some concerns | No concerns | No concerns | Moderate |
| SIRT_Che:TCM_Target_Che | 0 | Some concerns | Some concerns | No concerns | Some concerns | No concerns | No concerns | Low |
| SIRT_Che:Target | 0 | Some concerns | Some concerns | No concerns | Some concerns | No concerns | No concerns | Low |
| SIRT_Che:Target_Che | 0 | Some concerns | Some concerns | No concerns | Some concerns | No concerns | No concerns | Low |
| Target:TCM_Target_Che | 0 | No concerns | Low risk | No concerns | Some concerns | No concerns | No concerns | Moderate |

1. **Comparison of Therapeutic Modalities (Short-term)**

| Comparison | Number of studies | Within-study bias | Reporting bias | Indirectness | Imprecision | Heterogeneity | Incoherence | Confidence rating |
| --- | --- | --- | --- | --- | --- | --- | --- | --- |
| Che:ICI | 1 | Some concerns | Some concerns | Some concerns | No concerns | Some concerns | No concerns | Moderate |
| Che:Int_Che | 1 | No concerns | Low risk | No concerns | No concerns | Some concerns | No concerns | Moderate |
| Che:SIRT_Che | 1 | Some concerns | Some concerns | No concerns | No concerns | Major concerns | No concerns | Low |
| Che:Target_Che | 5 | Some concerns | Some concerns | No concerns | Some concerns | No concerns | No concerns | Moderate |
| NAT:Target | 4 | No concerns | Low risk | No concerns | Some concerns | No concerns | No concerns | Moderate |
| NAT:Target_Che | 1 | No concerns | Low risk | No concerns | Major concerns | No concerns | Major concerns | Low |
| Target:Target_Che | 3 | Some concerns | Some concerns | Some concerns | No concerns | Some concerns | No concerns | Moderate |
| Che:NAT | 0 | No concerns | Low risk | No concerns | Major concerns | No concerns | No concerns | Moderate |
| Che:Target | 0 | Some concerns | Some concerns | No concerns | No concerns | Some concerns | No concerns | Moderate |
| ICI:Int_Che | 0 | No concerns | Low risk | No concerns | No concerns | No concerns | No concerns | High |
| ICI:NAT | 0 | Some concerns | Some concerns | No concerns | Major concerns | No concerns | No concerns | Low |
| ICI:SIRT_Che | 0 | Some concerns | Some concerns | No concerns | No concerns | Major concerns | No concerns | Moderate |
| ICI:Target | 0 | Some concerns | Some concerns | Some concerns | Major concerns | No concerns | No concerns | Moderate |
| ICI:Target_Che | 0 | Some concerns | Some concerns | Some concerns | No concerns | Major concerns | No concerns | Low |
| Int_Che:NAT | 0 | No concerns | Low risk | No concerns | No concerns | Some concerns | No concerns | Moderate |
| Int_Che:SIRT_Che | 0 | No concerns | Low risk | No concerns | No concerns | Some concerns | No concerns | Moderate |
| Int_Che:Target | 0 | No concerns | Low risk | No concerns | No concerns | Some concerns | No concerns | Moderate |
| Int_Che:Target_Che | 0 | No concerns | Low risk | No concerns | No concerns | Some concerns | No concerns | Moderate |
| NAT:SIRT_Che | 0 | Some concerns | Some concerns | No concerns | No concerns | Major concerns | No concerns | Low |
| SIRT_Che:Target | 0 | Some concerns | Some concerns | No concerns | No concerns | Some concerns | No concerns | Moderate |
| SIRT_Che:Target_Che | 0 | Some concerns | Some concerns | No concerns | Major concerns | No concerns | No concerns | Low |

1. **Comparison of Treatment Quantities (Long-term)**

| Comparison | Number of studies | Within-study bias | Reporting bias | Indirectness | Imprecision | Heterogeneity | Incoherence | Confidence rating |
| --- | --- | --- | --- | --- | --- | --- | --- | --- |
| Monotherapy:No_treatment | 7 | No concerns | Low risk | No concerns | Some concerns | Major concerns | No concerns | Moderate |
| combination_therapy:Monotherapy | 8 | Some concerns | Low risk | No concerns | Major concerns | No concerns | No concerns | Low |
| combination_therapy:No_treatment | 1 | No concerns | Low risk | No concerns | Some concerns | No concerns | No concerns | Moderate |

1. **Comparison of Treatment Quantities (Short-term)**

| Comparison | Number of studies | Within-study bias | Reporting bias | Indirectness | Imprecision | Heterogeneity | Incoherence | Confidence rating |
| --- | --- | --- | --- | --- | --- | --- | --- | --- |
| Monotherapy:No_treatment | 6 | No concerns | Low risk | No concerns | No concerns | Major concerns | Some concerns | Moderate |
| combination_therapy:Monotherapy | 2 | No concerns | Low risk | No concerns | No concerns | Major concerns | Some concerns | Moderate |
| combination_therapy:No_treatment | 0 | No concerns | Low risk | No concerns | Major concerns | No concerns | Major concerns | Low |

1. **Comparison of Treatments (Long-term, First-line)**

**Note: Due to data limitations, evaluation of other dimensions was not supported**

| Comparison | Number of studies | Within-study bias | Reporting bias | Indirectness |
| --- | --- | --- | --- | --- |
| PAN:SC_PAN | 1 | Some concerns | Some concerns | Some concerns |
| SC:SC_Interferon | 1 | No concerns | Low risk | No concerns |
| SC:SC_PAN | 1 | Some concerns | Some concerns | No concerns |
| SC:SIRT_SC | 1 | Some concerns | Some concerns | No concerns |
| cet_SC:SC | 2 | No concerns | Low risk | No concerns |
| cet_intermittentSC:SC | 1 | No concerns | Low risk | No concerns |
| pembrolizumab:SC | 1 | Some concerns | Some concerns | Some concerns |
| cet_intermittentSC:cet_SC | 1 | No concerns | Low risk | No concerns |
| PAN:SC | 0 | Some concerns | Some concerns | No concerns |
| PAN:SC_Interferon | 0 | Some concerns | Some concerns | No concerns |
| PAN:SIRT_SC | 0 | Some concerns | Some concerns | No concerns |
| cet_SC:PAN | 0 | Some concerns | Some concerns | No concerns |
| cet_intermittentSC:PAN | 0 | Some concerns | Some concerns | No concerns |
| PAN:pembrolizumab | 0 | Some concerns | Some concerns | Some concerns |
| SC_Interferon:SC_PAN | 0 | No concerns | Low risk | No concerns |
| SC_Interferon:SIRT_SC | 0 | No concerns | Low risk | No concerns |
| cet_SC:SC_Interferon | 0 | No concerns | Low risk | No concerns |
| cet_intermittentSC:SC_Interferon | 0 | No concerns | Low risk | No concerns |
| pembrolizumab:SC_Interferon | 0 | No concerns | Low risk | No concerns |
| SC_PAN:SIRT_SC | 0 | Some concerns | Some concerns | No concerns |
| cet_SC:SC_PAN | 0 | Some concerns | Some concerns | No concerns |
| cet_intermittentSC:SC_PAN | 0 | No concerns | Low risk | No concerns |
| pembrolizumab:SC_PAN | 0 | Some concerns | Some concerns | No concerns |
| cet_SC:SIRT_SC | 0 | Some concerns | Some concerns | No concerns |
| cet_intermittentSC:SIRT_SC | 0 | No concerns | Low risk | No concerns |
| pembrolizumab:SIRT_SC | 0 | Some concerns | Some concerns | No concerns |
| cet_SC:pembrolizumab | 0 | Some concerns | Some concerns | Some concerns |
| cet_intermittentSC:pembrolizumab | 0 | No concerns | Low risk | No concerns |

1. **Comparison of Treatments (Long-term, Subsequent-line)**

**Note: Due to data limitations, evaluation of other dimensions was not supported**

| Comparison | Number of studies | Within-study bias | Reporting bias | Indirectness |
| --- | --- | --- | --- | --- |
| BEV:BEV_plus_Che | 1 | No concerns | Low risk | No concerns |
| BEV:No_active_treatment | 1 | No concerns | Low risk | No concerns |
| BEV_plus_Che:CET_plus_Che | 1 | Some concerns | Some concerns | No concerns |
| BEV_plus_Che:No_active_treatment | 1 | No concerns | Low risk | No concerns |
| CET:No_active_treatment | 1 | No concerns | Low risk | No concerns |
| CET_plus_Che:Che | 1 | Some concerns | Some concerns | No concerns |
| CET_plus_Che:enc_plus_CET | 1 | Some concerns | Some concerns | Some concerns |
| CET_plus_Che:enc_plus_CET_plus_bin | 1 | Some concerns | Some concerns | Some concerns |
| Che:Pan_Che | 1 | Some concerns | Some concerns | Some concerns |
| anlotinib:No_active_treatment | 1 | No concerns | Low risk | No concerns |
| famitinib:No_active_treatment | 1 | No concerns | Low risk | No concerns |
| nintedanib:No_active_treatment | 1 | No concerns | Low risk | No concerns |
| No_active_treatment:regorafenib | 2 | No concerns | Low risk | No concerns |
| enc_plus_CET:enc_plus_CET_plus_bin | 1 | Some concerns | Some concerns | Some concerns |
| BEV:CET | 0 | No concerns | Low risk | No concerns |
| BEV:CET_plus_Che | 0 | No concerns | Low risk | No concerns |
| BEV:Che | 0 | Some concerns | Some concerns | No concerns |
| BEV:Pan_Che | 0 | Some concerns | Some concerns | No concerns |
| anlotinib:BEV | 0 | No concerns | Low risk | No concerns |
| BEV:enc_plus_CET | 0 | Some concerns | Some concerns | No concerns |
| BEV:enc_plus_CET_plus_bin | 0 | Some concerns | Some concerns | No concerns |
| BEV:famitinib | 0 | No concerns | Low risk | No concerns |
| BEV:nintedanib | 0 | No concerns | Low risk | No concerns |
| BEV:regorafenib | 0 | No concerns | Low risk | No concerns |
| BEV_plus_Che:CET | 0 | No concerns | Low risk | No concerns |
| BEV_plus_Che:Che | 0 | Some concerns | Some concerns | No concerns |
| BEV_plus_Che:Pan_Che | 0 | Some concerns | Some concerns | No concerns |
| anlotinib:BEV_plus_Che | 0 | No concerns | Low risk | No concerns |
| BEV_plus_Che:enc_plus_CET | 0 | Some concerns | Some concerns | Some concerns |
| BEV_plus_Che:enc_plus_CET_plus_bin | 0 | Some concerns | Some concerns | Some concerns |
| BEV_plus_Che:famitinib | 0 | No concerns | Low risk | No concerns |
| BEV_plus_Che:nintedanib | 0 | No concerns | Low risk | No concerns |
| BEV_plus_Che:regorafenib | 0 | No concerns | Low risk | No concerns |
| CET:CET_plus_Che | 0 | No concerns | Low risk | No concerns |
| CET:Che | 0 | No concerns | Low risk | No concerns |
| CET:Pan_Che | 0 | Some concerns | Some concerns | No concerns |
| anlotinib:CET | 0 | No concerns | Low risk | No concerns |
| CET:enc_plus_CET | 0 | Some concerns | Some concerns | No concerns |
| CET:enc_plus_CET_plus_bin | 0 | Some concerns | Some concerns | No concerns |
| CET:famitinib | 0 | No concerns | Low risk | No concerns |
| CET:nintedanib | 0 | No concerns | Low risk | No concerns |
| CET:regorafenib | 0 | No concerns | Low risk | No concerns |
| CET_plus_Che:No_active_treatment | 0 | No concerns | Low risk | No concerns |
| CET_plus_Che:Pan_Che | 0 | Some concerns | Some concerns | No concerns |
| anlotinib:CET_plus_Che | 0 | No concerns | Low risk | No concerns |
| CET_plus_Che:famitinib | 0 | No concerns | Low risk | No concerns |
| CET_plus_Che:nintedanib | 0 | No concerns | Low risk | No concerns |
| CET_plus_Che:regorafenib | 0 | No concerns | Low risk | No concerns |
| Che:No_active_treatment | 0 | Some concerns | Some concerns | No concerns |
| anlotinib:Che | 0 | No concerns | Low risk | No concerns |
| Che:enc_plus_CET | 0 | Some concerns | Some concerns | Some concerns |
| Che:enc_plus_CET_plus_bin | 0 | Some concerns | Some concerns | Some concerns |
| Che:famitinib | 0 | No concerns | Low risk | No concerns |
| Che:nintedanib | 0 | No concerns | Low risk | No concerns |
| Che:regorafenib | 0 | No concerns | Low risk | No concerns |
| No_active_treatment:Pan_Che | 0 | Some concerns | Some concerns | No concerns |
| enc_plus_CET:No_active_treatment | 0 | Some concerns | Some concerns | No concerns |
| enc_plus_CET_plus_bin:No_active_treatment | 0 | Some concerns | Some concerns | No concerns |
| anlotinib:Pan_Che | 0 | Some concerns | Some concerns | No concerns |
| enc_plus_CET:Pan_Che | 0 | Some concerns | Some concerns | Some concerns |
| enc_plus_CET_plus_bin:Pan_Che | 0 | Some concerns | Some concerns | Some concerns |
| famitinib:Pan_Che | 0 | Some concerns | Some concerns | No concerns |
| nintedanib:Pan_Che | 0 | Some concerns | Some concerns | No concerns |
| Pan_Che:regorafenib | 0 | Some concerns | Some concerns | No concerns |
| anlotinib:enc_plus_CET | 0 | Some concerns | Some concerns | No concerns |
| anlotinib:enc_plus_CET_plus_bin | 0 | Some concerns | Some concerns | No concerns |
| anlotinib:famitinib | 0 | No concerns | Low risk | No concerns |
| anlotinib:nintedanib | 0 | No concerns | Low risk | No concerns |
| anlotinib:regorafenib | 0 | No concerns | Low risk | No concerns |
| enc_plus_CET:famitinib | 0 | Some concerns | Some concerns | No concerns |
| enc_plus_CET:nintedanib | 0 | Some concerns | Some concerns | No concerns |
| enc_plus_CET:regorafenib | 0 | Some concerns | Some concerns | No concerns |
| enc_plus_CET_plus_bin:famitinib | 0 | Some concerns | Some concerns | No concerns |
| enc_plus_CET_plus_bin:nintedanib | 0 | Some concerns | Some concerns | No concerns |
| enc_plus_CET_plus_bin:regorafenib | 0 | Some concerns | Some concerns | No concerns |
| famitinib:nintedanib | 0 | No concerns | Low risk | No concerns |
| famitinib:regorafenib | 0 | No concerns | Low risk | No concerns |
| nintedanib:regorafenib | 0 | No concerns | Low risk | No concerns |

1. **Comparison of Treatments (Long-term, Overall)**

| Comparison | Number of studies | Within-study bias | Reporting bias | Indirectness | Imprecision | Heterogeneity | Incoherence | Confidence rating |
| --- | --- | --- | --- | --- | --- | --- | --- | --- |
| Che:Che_plus_Interferon | 1 | No concerns | Low risk | No concerns | Major concerns | No concerns | No concerns | Moderate |
| Che:Pan_Che | 2 | Some concerns | Some concerns | No concerns | Major concerns | No concerns | No concerns | Moderate |
| Che:SIRT_plus_Che | 1 | Some concerns | Some concerns | No concerns | Major concerns | No concerns | No concerns | Moderate |
| cet_plus_Che:Che | 3 | Some concerns | Some concerns | No concerns | Major concerns | Major concerns | No concerns | Low |
| cet_plus_intermittentChe:Che | 1 | No concerns | Low risk | No concerns | Major concerns | No concerns | No concerns | Moderate |
| Che:pembrolizumab | 1 | Some concerns | Some concerns | Some concerns | Major concerns | Major concerns | No concerns | Low |
| Nintedanib:No_active_treatment | 1 | No concerns | Low risk | No concerns | Major concerns | No concerns | No concerns | Moderate |
| anlotinib:No_active_treatment | 1 | No concerns | Low risk | No concerns | Major concerns | No concerns | No concerns | Moderate |
| bev:No_active_treatment | 1 | No concerns | Low risk | No concerns | Major concerns | No concerns | No concerns | Moderate |
| bev_plus_Che:No_active_treatment | 1 | No concerns | Low risk | No concerns | Major concerns | No concerns | No concerns | Moderate |
| cet:No_active_treatment | 1 | No concerns | Low risk | No concerns | No concerns | Major concerns | No concerns | Moderate |
| famitinib:No_active_treatment | 1 | No concerns | Low risk | No concerns | No concerns | Major concerns | No concerns | Moderate |
| No_active_treatment:regorafenib | 2 | No concerns | Low risk | No concerns | Major concerns | No concerns | No concerns | Moderate |
| Pan:Pan_Che | 1 | Some concerns | Some concerns | Some concerns | Major concerns | Major concerns | No concerns | Low |
| bev_plus_Che:TCM_plus_bev_plus_Che | 1 | No concerns | Low risk | No concerns | Major concerns | No concerns | No concerns | Moderate |
| bev:bev_plus_Che | 1 | No concerns | Low risk | No concerns | Major concerns | No concerns | No concerns | Moderate |
| bev_plus_cet_plus_Che:bev_plus_Che | 1 | Some concerns | Some concerns | No concerns | No concerns | Major concerns | No concerns | Moderate |
| bev_plus_Che:cet_plus_Che | 1 | Some concerns | Some concerns | No concerns | Major concerns | No concerns | No concerns | Moderate |
| cet_plus_Che:cet_plus_intermittentChe | 1 | No concerns | Low risk | No concerns | Major concerns | No concerns | No concerns | Moderate |
| cet_plus_Che:encorafenib_plus_cet | 1 | Some concerns | Some concerns | Some concerns | Major concerns | No concerns | No concerns | Moderate |
| cet_plus_Che:encorafenib_plus_cet_plus_binimetinib | 1 | Some concerns | Some concerns | Some concerns | Major concerns | No concerns | No concerns | Moderate |
| encorafenib_plus_cet:encorafenib_plus_cet_plus_binimetinib | 1 | Some concerns | Some concerns | Some concerns | Major concerns | No concerns | No concerns | Moderate |
| Che:Nintedanib | 0 | No concerns | Low risk | No concerns | Major concerns | No concerns | No concerns | Moderate |
| Che:No_active_treatment | 0 | Some concerns | Some concerns | No concerns | Major concerns | No concerns | No concerns | Moderate |
| Che:Pan | 0 | Some concerns | Some concerns | Some concerns | Major concerns | No concerns | No concerns | Moderate |
| Che:TCM_plus_bev_plus_Che | 0 | Some concerns | Some concerns | No concerns | Major concerns | No concerns | No concerns | Moderate |
| anlotinib:Che | 0 | No concerns | Low risk | No concerns | Major concerns | No concerns | No concerns | Moderate |
| bev:Che | 0 | No concerns | Low risk | No concerns | Major concerns | No concerns | No concerns | Moderate |
| bev_plus_Che:Che | 0 | Some concerns | Some concerns | No concerns | Major concerns | No concerns | No concerns | Moderate |
| bev_plus_cet_plus_Che:Che | 0 | Some concerns | Some concerns | No concerns | Major concerns | Major concerns | No concerns | Low |
| cet:Che | 0 | No concerns | Low risk | No concerns | No concerns | Major concerns | No concerns | Moderate |
| Che:encorafenib_plus_cet | 0 | Some concerns | Some concerns | Some concerns | Major concerns | No concerns | No concerns | Moderate |
| Che:encorafenib_plus_cet_plus_binimetinib | 0 | Some concerns | Some concerns | Some concerns | Major concerns | No concerns | No concerns | Moderate |
| Che:famitinib | 0 | No concerns | Low risk | No concerns | Major concerns | No concerns | No concerns | Moderate |
| Che:regorafenib | 0 | No concerns | Low risk | No concerns | Major concerns | No concerns | No concerns | Moderate |
| Che_plus_Interferon:Nintedanib | 0 | No concerns | Low risk | No concerns | Major concerns | No concerns | No concerns | Moderate |
| Che_plus_Interferon:No_active_treatment | 0 | No concerns | Low risk | No concerns | Major concerns | No concerns | No concerns | Moderate |
| Che_plus_Interferon:Pan | 0 | Some concerns | Some concerns | No concerns | Major concerns | No concerns | No concerns | Moderate |
| Che_plus_Interferon:Pan_Che | 0 | No concerns | Low risk | No concerns | Major concerns | No concerns | No concerns | Moderate |
| Che_plus_Interferon:SIRT_plus_Che | 0 | No concerns | Low risk | No concerns | Major concerns | No concerns | No concerns | Moderate |
| Che_plus_Interferon:TCM_plus_bev_plus_Che | 0 | No concerns | Low risk | No concerns | Major concerns | No concerns | No concerns | Moderate |
| anlotinib:Che_plus_Interferon | 0 | No concerns | Low risk | No concerns | Major concerns | No concerns | No concerns | Moderate |
| bev:Che_plus_Interferon | 0 | No concerns | Low risk | No concerns | Major concerns | No concerns | No concerns | Moderate |
| bev_plus_Che:Che_plus_Interferon | 0 | Some concerns | Some concerns | No concerns | Major concerns | No concerns | No concerns | Moderate |
| bev_plus_cet_plus_Che:Che_plus_Interferon | 0 | Some concerns | Some concerns | No concerns | Major concerns | Major concerns | No concerns | Low |
| cet:Che_plus_Interferon | 0 | No concerns | Low risk | No concerns | Major concerns | No concerns | No concerns | Moderate |
| cet_plus_Che:Che_plus_Interferon | 0 | No concerns | Low risk | No concerns | Major concerns | No concerns | No concerns | Moderate |
| cet_plus_intermittentChe:Che_plus_Interferon | 0 | No concerns | Low risk | No concerns | Major concerns | No concerns | No concerns | Moderate |
| Che_plus_Interferon:encorafenib_plus_cet | 0 | Some concerns | Some concerns | No concerns | Major concerns | No concerns | No concerns | Moderate |
| Che_plus_Interferon:encorafenib_plus_cet_plus_binimetinib | 0 | Some concerns | Some concerns | No concerns | Major concerns | No concerns | No concerns | Moderate |
| Che_plus_Interferon:famitinib | 0 | No concerns | Low risk | No concerns | Major concerns | No concerns | No concerns | Moderate |
| Che_plus_Interferon:pembrolizumab | 0 | No concerns | Low risk | No concerns | Major concerns | No concerns | No concerns | Moderate |
| Che_plus_Interferon:regorafenib | 0 | No concerns | Low risk | No concerns | Major concerns | No concerns | No concerns | Moderate |
| Nintedanib:Pan | 0 | Some concerns | Some concerns | No concerns | Major concerns | Major concerns | No concerns | Low |
| Nintedanib:Pan_Che | 0 | No concerns | Low risk | No concerns | Major concerns | No concerns | No concerns | Moderate |
| Nintedanib:SIRT_plus_Che | 0 | No concerns | Low risk | No concerns | Major concerns | No concerns | No concerns | Moderate |
| Nintedanib:TCM_plus_bev_plus_Che | 0 | No concerns | Low risk | No concerns | Major concerns | No concerns | No concerns | Moderate |
| anlotinib:Nintedanib | 0 | No concerns | Low risk | No concerns | Major concerns | No concerns | No concerns | Moderate |
| bev:Nintedanib | 0 | No concerns | Low risk | No concerns | Major concerns | No concerns | No concerns | Moderate |
| bev_plus_Che:Nintedanib | 0 | No concerns | Low risk | No concerns | Major concerns | No concerns | No concerns | Moderate |
| bev_plus_cet_plus_Che:Nintedanib | 0 | No concerns | Low risk | No concerns | Major concerns | No concerns | No concerns | Moderate |
| cet:Nintedanib | 0 | No concerns | Low risk | No concerns | No concerns | Major concerns | No concerns | Moderate |
| cet_plus_Che:Nintedanib | 0 | No concerns | Low risk | No concerns | Major concerns | No concerns | No concerns | Moderate |
| cet_plus_intermittentChe:Nintedanib | 0 | No concerns | Low risk | No concerns | Major concerns | No concerns | No concerns | Moderate |
| encorafenib_plus_cet:Nintedanib | 0 | Some concerns | Some concerns | No concerns | Major concerns | No concerns | No concerns | Moderate |
| encorafenib_plus_cet_plus_binimetinib:Nintedanib | 0 | Some concerns | Some concerns | No concerns | Major concerns | No concerns | No concerns | Moderate |
| famitinib:Nintedanib | 0 | No concerns | Low risk | No concerns | Major concerns | No concerns | No concerns | Moderate |
| Nintedanib:pembrolizumab | 0 | No concerns | Low risk | No concerns | Major concerns | No concerns | No concerns | Moderate |
| Nintedanib:regorafenib | 0 | No concerns | Low risk | No concerns | Major concerns | No concerns | No concerns | Moderate |
| No_active_treatment:Pan | 0 | Some concerns | Some concerns | No concerns | Major concerns | Major concerns | No concerns | Low |
| No_active_treatment:Pan_Che | 0 | Some concerns | Some concerns | No concerns | Major concerns | No concerns | No concerns | Moderate |
| No_active_treatment:SIRT_plus_Che | 0 | Some concerns | Some concerns | No concerns | Major concerns | No concerns | No concerns | Moderate |
| No_active_treatment:TCM_plus_bev_plus_Che | 0 | No concerns | Low risk | No concerns | Major concerns | No concerns | No concerns | Moderate |
| bev_plus_cet_plus_Che:No_active_treatment | 0 | No concerns | Low risk | No concerns | Major concerns | No concerns | No concerns | Moderate |
| cet_plus_Che:No_active_treatment | 0 | No concerns | Low risk | No concerns | Major concerns | No concerns | No concerns | Moderate |
| cet_plus_intermittentChe:No_active_treatment | 0 | No concerns | Low risk | No concerns | Major concerns | No concerns | No concerns | Moderate |
| encorafenib_plus_cet:No_active_treatment | 0 | Some concerns | Some concerns | No concerns | Major concerns | No concerns | No concerns | Moderate |
| encorafenib_plus_cet_plus_binimetinib:No_active_treatment | 0 | Some concerns | Some concerns | No concerns | Major concerns | No concerns | No concerns | Moderate |
| No_active_treatment:pembrolizumab | 0 | Some concerns | Some concerns | No concerns | Major concerns | No concerns | No concerns | Moderate |
| Pan:SIRT_plus_Che | 0 | Some concerns | Some concerns | No concerns | Major concerns | No concerns | No concerns | Moderate |
| Pan:TCM_plus_bev_plus_Che | 0 | Some concerns | Some concerns | No concerns | Major concerns | No concerns | No concerns | Moderate |
| anlotinib:Pan | 0 | Some concerns | Some concerns | No concerns | Major concerns | No concerns | No concerns | Moderate |
| bev:Pan | 0 | Some concerns | Some concerns | No concerns | Major concerns | No concerns | No concerns | Moderate |
| bev_plus_Che:Pan | 0 | Some concerns | Some concerns | No concerns | Major concerns | No concerns | No concerns | Moderate |
| bev_plus_cet_plus_Che:Pan | 0 | Some concerns | Some concerns | No concerns | Major concerns | No concerns | No concerns | Moderate |
| cet:Pan | 0 | Some concerns | Some concerns | No concerns | No concerns | Major concerns | No concerns | Moderate |
| cet_plus_Che:Pan | 0 | Some concerns | Some concerns | Some concerns | Major concerns | Major concerns | No concerns | Low |
| cet_plus_intermittentChe:Pan | 0 | Some concerns | Some concerns | No concerns | Major concerns | No concerns | No concerns | Moderate |
| encorafenib_plus_cet:Pan | 0 | Some concerns | Some concerns | Some concerns | Major concerns | No concerns | No concerns | Moderate |
| encorafenib_plus_cet_plus_binimetinib:Pan | 0 | Some concerns | Some concerns | Some concerns | Major concerns | No concerns | No concerns | Moderate |
| famitinib:Pan | 0 | Some concerns | Some concerns | No concerns | Major concerns | No concerns | No concerns | Moderate |
| Pan:pembrolizumab | 0 | Some concerns | Some concerns | Some concerns | Major concerns | No concerns | No concerns | Moderate |
| Pan:regorafenib | 0 | Some concerns | Some concerns | No concerns | Major concerns | No concerns | No concerns | Moderate |
| Pan_Che:SIRT_plus_Che | 0 | Some concerns | Some concerns | No concerns | Major concerns | No concerns | No concerns | Moderate |
| Pan_Che:TCM_plus_bev_plus_Che | 0 | Some concerns | Some concerns | No concerns | Major concerns | No concerns | No concerns | Moderate |
| anlotinib:Pan_Che | 0 | No concerns | Low risk | No concerns | Major concerns | No concerns | No concerns | Moderate |
| bev:Pan_Che | 0 | Some concerns | Some concerns | No concerns | Major concerns | Major concerns | No concerns | Low |
| bev_plus_Che:Pan_Che | 0 | Some concerns | Some concerns | No concerns | Major concerns | No concerns | No concerns | Moderate |
| bev_plus_cet_plus_Che:Pan_Che | 0 | Some concerns | Some concerns | No concerns | Major concerns | No concerns | No concerns | Moderate |
| cet:Pan_Che | 0 | No concerns | Low risk | No concerns | No concerns | Major concerns | No concerns | Moderate |
| cet_plus_Che:Pan_Che | 0 | Some concerns | Some concerns | No concerns | Major concerns | No concerns | No concerns | Moderate |
| cet_plus_intermittentChe:Pan_Che | 0 | Some concerns | Some concerns | No concerns | Major concerns | No concerns | No concerns | Moderate |
| encorafenib_plus_cet:Pan_Che | 0 | Some concerns | Some concerns | Some concerns | Major concerns | No concerns | No concerns | Moderate |
| encorafenib_plus_cet_plus_binimetinib:Pan_Che | 0 | Some concerns | Some concerns | Some concerns | Major concerns | No concerns | No concerns | Moderate |
| famitinib:Pan_Che | 0 | No concerns | Low risk | No concerns | Major concerns | No concerns | No concerns | Moderate |
| Pan_Che:pembrolizumab | 0 | Some concerns | Some concerns | Some concerns | Major concerns | No concerns | No concerns | Moderate |
| Pan_Che:regorafenib | 0 | No concerns | Low risk | No concerns | Major concerns | No concerns | No concerns | Moderate |
| SIRT_plus_Che:TCM_plus_bev_plus_Che | 0 | Some concerns | Some concerns | No concerns | Major concerns | No concerns | No concerns | Moderate |
| anlotinib:SIRT_plus_Che | 0 | No concerns | Low risk | No concerns | Major concerns | No concerns | No concerns | Moderate |
| bev:SIRT_plus_Che | 0 | Some concerns | Some concerns | No concerns | Major concerns | Major concerns | No concerns | Low |
| bev_plus_Che:SIRT_plus_Che | 0 | Some concerns | Some concerns | No concerns | Major concerns | No concerns | No concerns | Moderate |
| bev_plus_cet_plus_Che:SIRT_plus_Che | 0 | Some concerns | Some concerns | No concerns | Major concerns | No concerns | No concerns | Moderate |
| cet:SIRT_plus_Che | 0 | No concerns | Low risk | No concerns | Major concerns | No concerns | No concerns | Moderate |
| cet_plus_Che:SIRT_plus_Che | 0 | Some concerns | Some concerns | No concerns | Major concerns | No concerns | No concerns | Moderate |
| cet_plus_intermittentChe:SIRT_plus_Che | 0 | Some concerns | Some concerns | No concerns | Major concerns | No concerns | No concerns | Moderate |
| encorafenib_plus_cet:SIRT_plus_Che | 0 | Some concerns | Some concerns | No concerns | Major concerns | No concerns | No concerns | Moderate |
| encorafenib_plus_cet_plus_binimetinib:SIRT_plus_Che | 0 | Some concerns | Some concerns | No concerns | Major concerns | No concerns | No concerns | Moderate |
| famitinib:SIRT_plus_Che | 0 | No concerns | Some concerns | No concerns | Major concerns | No concerns | No concerns | Moderate |
| pembrolizumab:SIRT_plus_Che | 0 | Some concerns | Low risk | No concerns | Major concerns | No concerns | No concerns | Moderate |
| regorafenib:SIRT_plus_Che | 0 | No concerns | Low risk | No concerns | Major concerns | No concerns | No concerns | Moderate |
| anlotinib:TCM_plus_bev_plus_Che | 0 | No concerns | Low risk | No concerns | Major concerns | No concerns | No concerns | Moderate |
| bev:TCM_plus_bev_plus_Che | 0 | No concerns | Low risk | No concerns | Major concerns | No concerns | No concerns | Moderate |
| bev_plus_cet_plus_Che:TCM_plus_bev_plus_Che | 0 | No concerns | Low risk | No concerns | Major concerns | No concerns | No concerns | Moderate |
| cet:TCM_plus_bev_plus_Che | 0 | No concerns | Low risk | No concerns | No concerns | Major concerns | No concerns | Moderate |
| cet_plus_Che:TCM_plus_bev_plus_Che | 0 | No concerns | Low risk | No concerns | Major concerns | No concerns | No concerns | Moderate |
| cet_plus_intermittentChe:TCM_plus_bev_plus_Che | 0 | No concerns | Low risk | No concerns | Major concerns | No concerns | No concerns | Moderate |
| encorafenib_plus_cet:TCM_plus_bev_plus_Che | 0 | Some concerns | Some concerns | No concerns | Major concerns | No concerns | No concerns | Moderate |
| encorafenib_plus_cet_plus_binimetinib:TCM_plus_bev_plus_Che | 0 | Some concerns | Some concerns | No concerns | Major concerns | Major concerns | No concerns | Low |
| famitinib:TCM_plus_bev_plus_Che | 0 | No concerns | Low risk | No concerns | Major concerns | No concerns | No concerns | Moderate |
| pembrolizumab:TCM_plus_bev_plus_Che | 0 | Some concerns | Some concerns | No concerns | Major concerns | No concerns | No concerns | Moderate |
| regorafenib:TCM_plus_bev_plus_Che | 0 | No concerns | Low risk | No concerns | Major concerns | No concerns | No concerns | Moderate |
| anlotinib:bev | 0 | No concerns | Low risk | No concerns | Major concerns | No concerns | No concerns | Moderate |
| anlotinib:bev_plus_Che | 0 | No concerns | Low risk | No concerns | Major concerns | No concerns | No concerns | Moderate |
| anlotinib:bev_plus_cet_plus_Che | 0 | No concerns | Low risk | No concerns | Major concerns | No concerns | No concerns | Moderate |
| anlotinib:cet | 0 | No concerns | Low risk | No concerns | No concerns | Major concerns | No concerns | Moderate |
| anlotinib:cet_plus_Che | 0 | No concerns | Low risk | No concerns | Major concerns | No concerns | No concerns | Moderate |
| anlotinib:cet_plus_intermittentChe | 0 | No concerns | Low risk | No concerns | Major concerns | No concerns | No concerns | Moderate |
| anlotinib:encorafenib_plus_cet | 0 | Some concerns | Some concerns | No concerns | Major concerns | Major concerns | No concerns | Low |
| anlotinib:encorafenib_plus_cet_plus_binimetinib | 0 | Some concerns | Some concerns | No concerns | Major concerns | No concerns | No concerns | Moderate |
| anlotinib:famitinib | 0 | No concerns | Low risk | No concerns | Major concerns | No concerns | No concerns | Moderate |
| anlotinib:pembrolizumab | 0 | No concerns | Low risk | No concerns | Major concerns | No concerns | No concerns | Moderate |
| anlotinib:regorafenib | 0 | No concerns | Low risk | No concerns | Major concerns | No concerns | No concerns | Moderate |
| bev:bev_plus_cet_plus_Che | 0 | No concerns | Low risk | No concerns | Major concerns | No concerns | No concerns | Moderate |
| bev:cet | 0 | No concerns | Low risk | No concerns | No concerns | Major concerns | No concerns | Moderate |
| bev:cet_plus_Che | 0 | No concerns | Low risk | No concerns | Major concerns | No concerns | No concerns | Moderate |
| bev:cet_plus_intermittentChe | 0 | No concerns | Low risk | No concerns | Major concerns | No concerns | No concerns | Moderate |
| bev:encorafenib_plus_cet | 0 | Some concerns | Some concerns | No concerns | Major concerns | No concerns | No concerns | Moderate |
| bev:encorafenib_plus_cet_plus_binimetinib | 0 | Some concerns | Some concerns | No concerns | Major concerns | No concerns | No concerns | Moderate |
| bev:famitinib | 0 | No concerns | Low risk | No concerns | Major concerns | No concerns | No concerns | Moderate |
| bev:pembrolizumab | 0 | Some concerns | Some concerns | No concerns | Major concerns | No concerns | No concerns | Moderate |
| bev:regorafenib | 0 | No concerns | Low risk | No concerns | Major concerns | No concerns | No concerns | Moderate |
| bev_plus_Che:cet | 0 | No concerns | Low risk | No concerns | No concerns | Major concerns | No concerns | Moderate |
| bev_plus_Che:cet_plus_intermittentChe | 0 | Some concerns | Some concerns | No concerns | Major concerns | No concerns | No concerns | Moderate |
| bev_plus_Che:encorafenib_plus_cet | 0 | Some concerns | Some concerns | Some concerns | Major concerns | Major concerns | No concerns | Low |
| bev_plus_Che:encorafenib_plus_cet_plus_binimetinib | 0 | Some concerns | Some concerns | Some concerns | Major concerns | No concerns | No concerns | Moderate |
| bev_plus_Che:famitinib | 0 | No concerns | Low risk | No concerns | Major concerns | No concerns | No concerns | Moderate |
| bev_plus_Che:pembrolizumab | 0 | Some concerns | Some concerns | No concerns | Major concerns | No concerns | No concerns | Moderate |
| bev_plus_Che:regorafenib | 0 | No concerns | Low risk | No concerns | Major concerns | No concerns | No concerns | Moderate |
| bev_plus_cet_plus_Che:cet | 0 | No concerns | Low risk | No concerns | No concerns | Major concerns | No concerns | Moderate |
| bev_plus_cet_plus_Che:cet_plus_Che | 0 | Some concerns | Some concerns | No concerns | Major concerns | No concerns | No concerns | Moderate |
| bev_plus_cet_plus_Che:cet_plus_intermittentChe | 0 | Some concerns | Some concerns | No concerns | Major concerns | No concerns | No concerns | Moderate |
| bev_plus_cet_plus_Che:encorafenib_plus_cet | 0 | Some concerns | Some concerns | No concerns | Major concerns | No concerns | No concerns | Moderate |
| bev_plus_cet_plus_Che:encorafenib_plus_cet_plus_binimetinib | 0 | Some concerns | Some concerns | No concerns | Major concerns | No concerns | No concerns | Moderate |
| bev_plus_cet_plus_Che:famitinib | 0 | No concerns | Low risk | No concerns | Major concerns | No concerns | No concerns | Moderate |
| bev_plus_cet_plus_Che:pembrolizumab | 0 | Some concerns | Some concerns | No concerns | Major concerns | Major concerns | No concerns | Low |
| bev_plus_cet_plus_Che:regorafenib | 0 | No concerns | Low risk | No concerns | Major concerns | No concerns | No concerns | Moderate |
| cet:cet_plus_Che | 0 | No concerns | Low risk | No concerns | No concerns | Major concerns | No concerns | Moderate |
| cet:cet_plus_intermittentChe | 0 | No concerns | Low risk | No concerns | No concerns | Major concerns | No concerns | Moderate |
| cet:encorafenib_plus_cet | 0 | Some concerns | Some concerns | No concerns | No concerns | Major concerns | No concerns | Moderate |
| cet:encorafenib_plus_cet_plus_binimetinib | 0 | Some concerns | Some concerns | No concerns | No concerns | Major concerns | No concerns | Moderate |
| cet:famitinib | 0 | No concerns | Low risk | No concerns | No concerns | Major concerns | No concerns | Moderate |
| cet:pembrolizumab | 0 | No concerns | Low risk | No concerns | Major concerns | No concerns | No concerns | Moderate |
| cet:regorafenib | 0 | No concerns | Low risk | No concerns | No concerns | Major concerns | No concerns | Moderate |
| cet_plus_Che:famitinib | 0 | No concerns | Low risk | No concerns | Major concerns | No concerns | No concerns | Moderate |
| cet_plus_Che:pembrolizumab | 0 | Some concerns | Some concerns | Some concerns | Major concerns | Major concerns | No concerns | Low |
| cet_plus_Che:regorafenib | 0 | No concerns | Low risk | No concerns | Major concerns | No concerns | No concerns | Moderate |
| cet_plus_intermittentChe:encorafenib_plus_cet | 0 | Some concerns | Some concerns | Some concerns | Major concerns | No concerns | No concerns | Moderate |
| cet_plus_intermittentChe:encorafenib_plus_cet_plus_binimetinib | 0 | Some concerns | Some concerns | Some concerns | Major concerns | No concerns | No concerns | Moderate |
| cet_plus_intermittentChe:famitinib | 0 | No concerns | Low risk | No concerns | Major concerns | No concerns | No concerns | Moderate |
| cet_plus_intermittentChe:pembrolizumab | 0 | Some concerns | Some concerns | No concerns | Major concerns | No concerns | No concerns | Moderate |
| cet_plus_intermittentChe:regorafenib | 0 | No concerns | Low risk | No concerns | Major concerns | No concerns | No concerns | Moderate |
| encorafenib_plus_cet:famitinib | 0 | Some concerns | Some concerns | No concerns | Major concerns | No concerns | No concerns | Moderate |
| encorafenib_plus_cet:pembrolizumab | 0 | Some concerns | Some concerns | Some concerns | Major concerns | No concerns | No concerns | Moderate |
| encorafenib_plus_cet:regorafenib | 0 | Some concerns | Some concerns | No concerns | Major concerns | Major concerns | No concerns | Low |
| encorafenib_plus_cet_plus_binimetinib:famitinib | 0 | Some concerns | Some concerns | No concerns | Major concerns | No concerns | No concerns | Moderate |
| encorafenib_plus_cet_plus_binimetinib:pembrolizumab | 0 | Some concerns | Some concerns | Some concerns | Major concerns | No concerns | No concerns | Moderate |
| encorafenib_plus_cet_plus_binimetinib:regorafenib | 0 | Some concerns | Some concerns | No concerns | Major concerns | No concerns | No concerns | Moderate |
| famitinib:pembrolizumab | 0 | No concerns | Low risk | No concerns | Major concerns | No concerns | No concerns | Moderate |
| famitinib:regorafenib | 0 | No concerns | Low risk | No concerns | No concerns | Major concerns | No concerns | Moderate |
| pembrolizumab:regorafenib | 0 | No concerns | Low risk | No concerns | Major concerns | No concerns | No concerns | Moderate |

1. **Comparison of Treatments (Short-term, First-line)**

**Note: Due to data limitations, evaluation of other dimensions was not supported**

| Comparison | Number of studies | Within-study bias | Reporting bias | Indirectness |
| --- | --- | --- | --- | --- |
| Che:Interferon_plus_Che | 1 | No concerns | Low risk | No concerns |
| Che:SIRT_plus_Che | 1 | Some concerns | Some concerns | No concerns |
| cet_plus_Che:Che | 2 | No concerns | Low risk | No concerns |
| cet_plus_intermittentChe:Che | 1 | No concerns | Low risk | No concerns |
| Che:panitumumab_plus_Che | 1 | Some concerns | Some concerns | No concerns |
| Che:pembrolizumab | 1 | Some concerns | Some concerns | Some concerns |
| cet_plus_Che:cet_plus_intermittentChe | 1 | No concerns | Low risk | No concerns |
| panitumumab:panitumumab_plus_Che | 1 | No concerns | Low risk | Some concerns |
| Che:panitumumab | 0 | No concerns | Low risk | No concerns |
| Interferon_plus_Che:SIRT_plus_Che | 0 | No concerns | Low risk | No concerns |
| cet_plus_Che:Interferon_plus_Che | 0 | No concerns | Low risk | No concerns |
| cet_plus_intermittentChe:Interferon_plus_Che | 0 | No concerns | Low risk | No concerns |
| Interferon_plus_Che:panitumumab | 0 | No concerns | Low risk | No concerns |
| Interferon_plus_Che:panitumumab_plus_Che | 0 | No concerns | Low risk | No concerns |
| Interferon_plus_Che:pembrolizumab | 0 | No concerns | Low risk | No concerns |
| cet_plus_Che:SIRT_plus_Che | 0 | Some concerns | Some concerns | No concerns |
| cet_plus_intermittentChe:SIRT_plus_Che | 0 | No concerns | Low risk | No concerns |
| panitumumab:SIRT_plus_Che | 0 | Some concerns | Some concerns | No concerns |
| panitumumab_plus_Che:SIRT_plus_Che | 0 | Some concerns | Some concerns | No concerns |
| pembrolizumab:SIRT_plus_Che | 0 | Some concerns | Some concerns | No concerns |
| cet_plus_Che:panitumumab | 0 | No concerns | Low risk | No concerns |
| cet_plus_Che:panitumumab_plus_Che | 0 | Some concerns | Some concerns | No concerns |
| cet_plus_Che:pembrolizumab | 0 | Some concerns | Some concerns | Some concerns |
| cet_plus_intermittentChe:panitumumab | 0 | No concerns | Low risk | No concerns |
| cet_plus_intermittentChe:panitumumab_plus_Che | 0 | No concerns | Low risk | No concerns |
| cet_plus_intermittentChe:pembrolizumab | 0 | No concerns | Low risk | No concerns |
| panitumumab:pembrolizumab | 0 | Some concerns | Some concerns | Some concerns |
| panitumumab_plus_Che:pembrolizumab | 0 | Some concerns | Some concerns | No concerns |

1. **Comparison of Treatments (Short-term, Subsequent-line)**

**Note: Due to data limitations, evaluation of other dimensions was not supported**

| Comparison | Number of studies | Within-study bias | Reporting bias | Indirectness |
| --- | --- | --- | --- | --- |
| cet_plus_Che:Che | 1 | Some concerns | Some concerns | No concerns |
| anlotinib:NAT | 1 | No concerns | Low risk | No concerns |
| bev:NAT | 1 | No concerns | Low risk | No concerns |
| bev_plus_Che:NAT | 1 | No concerns | Low risk | No concerns |
| cet:NAT | 1 | No concerns | Low risk | No concerns |
| famitinib:NAT | 1 | No concerns | Low risk | No concerns |
| bev:bev_plus_Che | 1 | No concerns | Low risk | No concerns |
| bev_plus_Che:cet_plus_Che | 1 | Some concerns | Some concerns | No concerns |
| cet_plus_Che:encorafenib_plus_cet | 1 | Some concerns | Some concerns | Some concerns |
| cet_plus_Che:encorafenib_plus_cet_plus_binimetinib | 1 | Some concerns | Some concerns | No concerns |
| encorafenib_plus_cet:encorafenib_plus_cet_plus_binimetinib | 1 | Some concerns | Some concerns | Some concerns |
| Che:NAT | 0 | Some concerns | Some concerns | No concerns |
| anlotinib:Che | 0 | No concerns | Low risk | No concerns |
| bev:Che | 0 | Some concerns | Some concerns | No concerns |
| bev_plus_Che:Che | 0 | Some concerns | Some concerns | No concerns |
| cet:Che | 0 | No concerns | Low risk | No concerns |
| Che:encorafenib_plus_cet | 0 | Some concerns | Some concerns | Some concerns |
| Che:encorafenib_plus_cet_plus_binimetinib | 0 | Some concerns | Some concerns | Some concerns |
| Che:famitinib | 0 | No concerns | Low risk | No concerns |
| cet_plus_Che:NAT | 0 | No concerns | Low risk | No concerns |
| encorafenib_plus_cet:NAT | 0 | Some concerns | Some concerns | No concerns |
| encorafenib_plus_cet_plus_binimetinib:NAT | 0 | Some concerns | Some concerns | No concerns |
| anlotinib:bev | 0 | No concerns | Low risk | No concerns |
| anlotinib:bev_plus_Che | 0 | No concerns | Low risk | No concerns |
| anlotinib:cet | 0 | No concerns | Low risk | No concerns |
| anlotinib:cet_plus_Che | 0 | No concerns | Low risk | No concerns |
| anlotinib:encorafenib_plus_cet | 0 | Some concerns | Some concerns | No concerns |
| anlotinib:encorafenib_plus_cet_plus_binimetinib | 0 | Some concerns | Some concerns | No concerns |
| anlotinib:famitinib | 0 | No concerns | Low risk | No concerns |
| bev:cet | 0 | No concerns | Low risk | No concerns |
| bev:cet_plus_Che | 0 | No concerns | Low risk | No concerns |
| bev:encorafenib_plus_cet | 0 | Some concerns | Some concerns | No concerns |
| bev:encorafenib_plus_cet_plus_binimetinib | 0 | Some concerns | Some concerns | No concerns |
| bev:famitinib | 0 | No concerns | Low risk | No concerns |
| bev_plus_Che:cet | 0 | No concerns | Low risk | No concerns |
| bev_plus_Che:encorafenib_plus_cet | 0 | Some concerns | Some concerns | Some concerns |
| bev_plus_Che:encorafenib_plus_cet_plus_binimetinib | 0 | Some concerns | Some concerns | Some concerns |
| bev_plus_Che:famitinib | 0 | No concerns | Low risk | No concerns |
| cet:cet_plus_Che | 0 | No concerns | Low risk | No concerns |
| cet:encorafenib_plus_cet | 0 | Some concerns | Some concerns | No concerns |
| cet:encorafenib_plus_cet_plus_binimetinib | 0 | Some concerns | Some concerns | No concerns |
| cet:famitinib | 0 | No concerns | Low risk | No concerns |
| cet_plus_Che:famitinib | 0 | No concerns | Low risk | No concerns |
| encorafenib_plus_cet:famitinib | 0 | Some concerns | Some concerns | No concerns |
| encorafenib_plus_cet_plus_binimetinib:famitinib | 0 | Some concerns | Some concerns | No concerns |

1. **Comparison of Treatments (Short-term, Overall)**

| Comparison | Number of studies | Within-study bias | Reporting bias | Indirectness | Imprecision | Heterogeneity | Incoherence | Confidence rating |
| --- | --- | --- | --- | --- | --- | --- | --- | --- |
| Che:Che_plus_Interferon | 1 | No concerns | Low risk | No concerns | No concerns | Major concerns | Some concerns | Moderate |
| Che:PAN_Che | 1 | Some concerns | Some concerns | No concerns | Major concerns | No concerns | Some concerns | Moderate |
| Che:SIRT_plus_Che | 1 | Some concerns | Some concerns | No concerns | No concerns | Major concerns | Some concerns | Moderate |
| cet_plus_Che:Che | 3 | Some concerns | Some concerns | No concerns | Major concerns | No concerns | Some concerns | Moderate |
| cet_plus_intermittentChe:Che | 1 | No concerns | Low risk | No concerns | Major concerns | No concerns | No concerns | Moderate |
| Che:pembrolizumab | 1 | Some concerns | Some concerns | Some concerns | No concerns | Major concerns | Some concerns | Low |
| anlotinib:NAT | 1 | No concerns | Low risk | No concerns | Major concerns | No concerns | Some concerns | Moderate |
| bev:NAT | 1 | No concerns | Low risk | No concerns | Major concerns | No concerns | Some concerns | Moderate |
| bev_plus_Che:NAT | 1 | No concerns | Low risk | No concerns | No concerns | Major concerns | Some concerns | Moderate |
| cet:NAT | 1 | No concerns | Low risk | No concerns | No concerns | Major concerns | Some concerns | Moderate |
| famitinib:NAT | 1 | No concerns | Low risk | No concerns | Major concerns | No concerns | Some concerns | Moderate |
| PAN:PAN_Che | 1 | Some concerns | Some concerns | Some concerns | Major concerns | No concerns | Some concerns | Low |
| bev:bev_plus_Che | 1 | No concerns | Low risk | No concerns | No concerns | Major concerns | Some concerns | Moderate |
| bev_plus_Che:cet_plus_Che | 1 | Some concerns | Some concerns | No concerns | Major concerns | No concerns | Some concerns | Moderate |
| cet_plus_Che:cet_plus_intermittentChe | 1 | No concerns | Low risk | No concerns | Major concerns | No concerns | No concerns | Moderate |
| cet_plus_Che:encorafenib_plus_cet | 1 | Some concerns | Some concerns | Some concerns | Major concerns | No concerns | Some concerns | Low |
| cet_plus_Che:encorafenib_plus_cet_plus_binimetinib | 1 | Some concerns | Some concerns | Some concerns | Major concerns | No concerns | Some concerns | Low |
| encorafenib_plus_cet:encorafenib_plus_cet_plus_binimetinib | 1 | Some concerns | Some concerns | Some concerns | Major concerns | No concerns | Some concerns | Low |
| Che:NAT | 0 | Some concerns | Some concerns | No concerns | Major concerns | No concerns | Some concerns | Moderate |
| Che:PAN | 0 | Some concerns | Some concerns | No concerns | Major concerns | No concerns | Some concerns | Moderate |
| anlotinib:Che | 0 | No concerns | Low risk | No concerns | Major concerns | No concerns | Some concerns | Moderate |
| bev:Che | 0 | Some concerns | Some concerns | No concerns | Major concerns | No concerns | Some concerns | Moderate |
| bev_plus_Che:Che | 0 | Some concerns | Some concerns | No concerns | Major concerns | No concerns | Some concerns | Moderate |
| cet:Che | 0 | No concerns | Low risk | No concerns | No concerns | Major concerns | Some concerns | Moderate |
| Che:encorafenib_plus_cet | 0 | Some concerns | Some concerns | Some concerns | Major concerns | No concerns | Some concerns | Low |
| Che:encorafenib_plus_cet_plus_binimetinib | 0 | Some concerns | Some concerns | Some concerns | Major concerns | No concerns | Some concerns | Low |
| Che:famitinib | 0 | No concerns | Low risk | No concerns | Major concerns | No concerns | Some concerns | Moderate |
| Che_plus_Interferon:NAT | 0 | No concerns | Low risk | No concerns | No concerns | Major concerns | Some concerns | Moderate |
| Che_plus_Interferon:PAN | 0 | Some concerns | Some concerns | No concerns | Major concerns | No concerns | Some concerns | Moderate |
| Che_plus_Interferon:PAN_Che | 0 | No concerns | Low risk | No concerns | Major concerns | No concerns | Some concerns | Moderate |
| Che_plus_Interferon:SIRT_plus_Che | 0 | No concerns | Low risk | No concerns | No concerns | Major concerns | Some concerns | Moderate |
| anlotinib:Che_plus_Interferon | 0 | No concerns | Low risk | No concerns | No concerns | Major concerns | Some concerns | Moderate |
| bev:Che_plus_Interferon | 0 | No concerns | Low risk | No concerns | No concerns | Major concerns | Some concerns | Moderate |
| bev_plus_Che:Che_plus_Interferon | 0 | Some concerns | Some concerns | No concerns | No concerns | Major concerns | Some concerns | Moderate |
| cet:Che_plus_Interferon | 0 | No concerns | Low risk | No concerns | No concerns | Major concerns | Some concerns | Moderate |
| cet_plus_Che:Che_plus_Interferon | 0 | No concerns | Low risk | No concerns | No concerns | Major concerns | Some concerns | Moderate |
| cet_plus_intermittentChe:Che_plus_Interferon | 0 | No concerns | Low risk | No concerns | No concerns | Major concerns | Some concerns | Moderate |
| Che_plus_Interferon:encorafenib_plus_cet | 0 | Some concerns | Some concerns | No concerns | No concerns | Major concerns | Some concerns | Moderate |
| Che_plus_Interferon:encorafenib_plus_cet_plus_binimetinib | 0 | Some concerns | Some concerns | No concerns | Major concerns | No concerns | Some concerns | Moderate |
| Che_plus_Interferon:famitinib | 0 | No concerns | Low risk | No concerns | No concerns | Major concerns | Some concerns | Moderate |
| Che_plus_Interferon:pembrolizumab | 0 | No concerns | Low risk | No concerns | No concerns | Major concerns | Some concerns | Moderate |
| NAT:PAN | 0 | Some concerns | Some concerns | No concerns | Major concerns | No concerns | Some concerns | Moderate |
| NAT:PAN_Che | 0 | Some concerns | Some concerns | No concerns | Major concerns | No concerns | Some concerns | Moderate |
| NAT:SIRT_plus_Che | 0 | Some concerns | Some concerns | No concerns | No concerns | Major concerns | Some concerns | Moderate |
| cet_plus_Che:NAT | 0 | No concerns | Low risk | No concerns | Major concerns | No concerns | Some concerns | Moderate |
| cet_plus_intermittentChe:NAT | 0 | No concerns | Low risk | No concerns | Major concerns | No concerns | Some concerns | Moderate |
| encorafenib_plus_cet:NAT | 0 | Some concerns | Some concerns | No concerns | Major concerns | No concerns | Some concerns | Moderate |
| encorafenib_plus_cet_plus_binimetinib:NAT | 0 | Some concerns | Some concerns | No concerns | Major concerns | No concerns | Some concerns | Moderate |
| NAT:pembrolizumab | 0 | Some concerns | Some concerns | No concerns | Major concerns | No concerns | Some concerns | Moderate |
| PAN:SIRT_plus_Che | 0 | Some concerns | Some concerns | No concerns | Major concerns | No concerns | Some concerns | Moderate |
| anlotinib:PAN | 0 | Some concerns | Some concerns | No concerns | Major concerns | No concerns | Some concerns | Moderate |
| bev:PAN | 0 | Some concerns | Some concerns | No concerns | Major concerns | No concerns | Some concerns | Moderate |
| bev_plus_Che:PAN | 0 | Some concerns | Some concerns | No concerns | Major concerns | No concerns | Some concerns | Moderate |
| cet:PAN | 0 | Some concerns | Some concerns | No concerns | No concerns | Major concerns | Some concerns | Moderate |
| cet_plus_Che:PAN | 0 | Some concerns | Some concerns | No concerns | Major concerns | No concerns | Some concerns | Moderate |
| cet_plus_intermittentChe:PAN | 0 | Some concerns | Some concerns | No concerns | Major concerns | No concerns | Some concerns | Moderate |
| encorafenib_plus_cet:PAN | 0 | Some concerns | Some concerns | Some concerns | Major concerns | No concerns | Some concerns | Low |
| encorafenib_plus_cet_plus_binimetinib:PAN | 0 | Some concerns | Some concerns | Some concerns | Major concerns | No concerns | Some concerns | Low |
| famitinib:PAN | 0 | Some concerns | Some concerns | No concerns | Major concerns | No concerns | Some concerns | Moderate |
| PAN:pembrolizumab | 0 | Some concerns | Some concerns | Some concerns | Major concerns | No concerns | Some concerns | Low |
| PAN_Che:SIRT_plus_Che | 0 | Some concerns | Some concerns | No concerns | Major concerns | No concerns | Some concerns | Moderate |
| anlotinib:PAN_Che | 0 | Some concerns | Some concerns | No concerns | Major concerns | No concerns | Some concerns | Moderate |
| bev:PAN_Che | 0 | Some concerns | Some concerns | No concerns | Major concerns | No concerns | Some concerns | Moderate |
| bev_plus_Che:PAN_Che | 0 | Some concerns | Some concerns | No concerns | Major concerns | No concerns | Some concerns | Moderate |
| cet:PAN_Che | 0 | Some concerns | Some concerns | No concerns | No concerns | Major concerns | Some concerns | Moderate |
| cet_plus_Che:PAN_Che | 0 | Some concerns | Some concerns | No concerns | Major concerns | No concerns | Some concerns | Moderate |
| cet_plus_intermittentChe:PAN_Che | 0 | Some concerns | Some concerns | No concerns | Major concerns | No concerns | Some concerns | Moderate |
| encorafenib_plus_cet:PAN_Che | 0 | Some concerns | Some concerns | No concerns | Major concerns | No concerns | Some concerns | Moderate |
| encorafenib_plus_cet_plus_binimetinib:PAN_Che | 0 | Some concerns | Some concerns | No concerns | Major concerns | No concerns | Some concerns | Moderate |
| famitinib:PAN_Che | 0 | Some concerns | Some concerns | No concerns | Major concerns | No concerns | Some concerns | Moderate |
| PAN_Che:pembrolizumab | 0 | Some concerns | Some concerns | No concerns | Major concerns | No concerns | Some concerns | Moderate |
| anlotinib:SIRT_plus_Che | 0 | Some concerns | Some concerns | No concerns | Major concerns | No concerns | Some concerns | Moderate |
| bev:SIRT_plus_Che | 0 | Some concerns | Some concerns | No concerns | No concerns | Major concerns | Some concerns | Moderate |
| bev_plus_Che:SIRT_plus_Che | 0 | Some concerns | Some concerns | No concerns | Major concerns | No concerns | Some concerns | Moderate |
| cet:SIRT_plus_Che | 0 | Some concerns | Some concerns | No concerns | No concerns | Major concerns | Some concerns | Moderate |
| cet_plus_Che:SIRT_plus_Che | 0 | Some concerns | Some concerns | No concerns | Major concerns | No concerns | Some concerns | Low |
| cet_plus_intermittentChe:SIRT_plus_Che | 0 | Some concerns | Some concerns | No concerns | Major concerns | No concerns | Some concerns | Moderate |
| encorafenib_plus_cet:SIRT_plus_Che | 0 | Some concerns | Some concerns | No concerns | Major concerns | No concerns | Some concerns | Moderate |
| encorafenib_plus_cet_plus_binimetinib:SIRT_plus_Che | 0 | Some concerns | Some concerns | No concerns | Major concerns | No concerns | Some concerns | Moderate |
| famitinib:SIRT_plus_Che | 0 | Some concerns | Some concerns | No concerns | No concerns | Major concerns | Some concerns | Moderate |
| pembrolizumab:SIRT_plus_Che | 0 | Some concerns | Some concerns | No concerns | No concerns | Major concerns | Some concerns | Moderate |
| anlotinib:bev | 0 | No concerns | Low risk | No concerns | Major concerns | No concerns | Some concerns | Moderate |
| anlotinib:bev_plus_Che | 0 | No concerns | Low risk | No concerns | Major concerns | No concerns | Some concerns | Moderate |
| anlotinib:cet | 0 | No concerns | Low risk | No concerns | No concerns | Major concerns | Some concerns | Moderate |
| anlotinib:cet_plus_Che | 0 | No concerns | Low risk | No concerns | Major concerns | No concerns | Some concerns | Moderate |
| anlotinib:cet_plus_intermittentChe | 0 | No concerns | Low risk | No concerns | Major concerns | No concerns | Some concerns | Moderate |
| anlotinib:encorafenib_plus_cet | 0 | Some concerns | Some concerns | No concerns | Major concerns | No concerns | Some concerns | Moderate |
| anlotinib:encorafenib_plus_cet_plus_binimetinib | 0 | Some concerns | Some concerns | No concerns | Major concerns | No concerns | Some concerns | Moderate |
| anlotinib:famitinib | 0 | No concerns | Low risk | No concerns | Major concerns | No concerns | Some concerns | Moderate |
| anlotinib:pembrolizumab | 0 | Some concerns | Some concerns | No concerns | Major concerns | No concerns | Some concerns | Moderate |
| bev:cet | 0 | No concerns | Low risk | No concerns | No concerns | Major concerns | Some concerns | Moderate |
| bev:cet_plus_Che | 0 | No concerns | Low risk | No concerns | Major concerns | No concerns | Some concerns | Moderate |
| bev:cet_plus_intermittentChe | 0 | No concerns | Low risk | No concerns | Major concerns | No concerns | Some concerns | Moderate |
| bev:encorafenib_plus_cet | 0 | Some concerns | Some concerns | No concerns | Major concerns | No concerns | Some concerns | Moderate |
| bev:encorafenib_plus_cet_plus_binimetinib | 0 | Some concerns | Some concerns | No concerns | Major concerns | No concerns | Some concerns | Moderate |
| bev:famitinib | 0 | No concerns | Low risk | No concerns | Major concerns | No concerns | Some concerns | Moderate |
| bev:pembrolizumab | 0 | Some concerns | Some concerns | No concerns | Major concerns | No concerns | Some concerns | Moderate |
| bev_plus_Che:cet | 0 | No concerns | Low risk | No concerns | No concerns | Major concerns | Some concerns | Moderate |
| bev_plus_Che:cet_plus_intermittentChe | 0 | Some concerns | Some concerns | No concerns | Major concerns | No concerns | Some concerns | Moderate |
| bev_plus_Che:encorafenib_plus_cet | 0 | Some concerns | Some concerns | Some concerns | Major concerns | No concerns | Some concerns | Low |
| bev_plus_Che:encorafenib_plus_cet_plus_binimetinib | 0 | Some concerns | Some concerns | Some concerns | Major concerns | No concerns | Some concerns | Low |
| bev_plus_Che:famitinib | 0 | No concerns | Low risk | No concerns | No concerns | Major concerns | Some concerns | Moderate |
| bev_plus_Che:pembrolizumab | 0 | Some concerns | Some concerns | No concerns | Major concerns | No concerns | Some concerns | Moderate |
| cet:cet_plus_Che | 0 | No concerns | Low risk | No concerns | No concerns | Major concerns | Some concerns | Moderate |
| cet:cet_plus_intermittentChe | 0 | No concerns | Low risk | No concerns | No concerns | Major concerns | Some concerns | Moderate |
| cet:encorafenib_plus_cet | 0 | Some concerns | Some concerns | No concerns | No concerns | Major concerns | Some concerns | Moderate |
| cet:encorafenib_plus_cet_plus_binimetinib | 0 | Some concerns | Some concerns | No concerns | No concerns | Major concerns | Some concerns | Moderate |
| cet:famitinib | 0 | No concerns | Low risk | No concerns | No concerns | Major concerns | Some concerns | Moderate |
| cet:pembrolizumab | 0 | Some concerns | Some concerns | No concerns | Major concerns | No concerns | Some concerns | Moderate |
| cet_plus_Che:famitinib | 0 | No concerns | Low risk | No concerns | Major concerns | No concerns | Some concerns | Moderate |
| cet_plus_Che:pembrolizumab | 0 | Some concerns | Some concerns | Some concerns | No concerns | Major concerns | Some concerns | Low |
| cet_plus_intermittentChe:encorafenib_plus_cet | 0 | Some concerns | Some concerns | Some concerns | Major concerns | No concerns | Some concerns | Low |
| cet_plus_intermittentChe:encorafenib_plus_cet_plus_binimetinib | 0 | Some concerns | Some concerns | Some concerns | Major concerns | No concerns | Some concerns | Low |
| cet_plus_intermittentChe:famitinib | 0 | No concerns | Low risk | No concerns | Major concerns | No concerns | Some concerns | Moderate |
| cet_plus_intermittentChe:pembrolizumab | 0 | Some concerns | Some concerns | No concerns | No concerns | Major concerns | Some concerns | Moderate |
| encorafenib_plus_cet:famitinib | 0 | Some concerns | Some concerns | No concerns | Major concerns | No concerns | Some concerns | Moderate |
| encorafenib_plus_cet:pembrolizumab | 0 | Some concerns | Some concerns | Some concerns | Major concerns | No concerns | Some concerns | Low |
| encorafenib_plus_cet_plus_binimetinib:famitinib | 0 | Some concerns | Some concerns | No concerns | Major concerns | No concerns | Some concerns | Moderate |
| encorafenib_plus_cet_plus_binimetinib:pembrolizumab | 0 | Some concerns | Some concerns | Some concerns | No concerns | Major concerns | Some concerns | Low |
| famitinib:pembrolizumab | 0 | Some concerns | Some concerns | No concerns | Major concerns | No concerns | Some concerns | Moderate |

1. **Transitivity**

**Fig.S1 ECOG**

**Fig.S2 Gender**

**Fig.S3 Age**

1. **Direct Evidence Contribution Matrix**

Abbreviation ANL: anlotinib; BEV: bevacizumab; BEV+Che:bevacizumab plus chemotherapy;CET: cetuximab; CET+Che: cetuximab plus chemotherapy; CET+inter Che: cetuximab plus intermittent chemotherapy; CET+BEV+Che: cetuximab combined with bevacizumab plus chemotherapy; Che: chemotherapy; Che+INT: interferon plus chemotherapy; ENC+CET: encorafenib plus cetuximab; ENC+CET+BIN: encorafenib combined with cetuximab plus binimetinib; FAM: famitinib; ICI: Immunotherapy; NAT: No active treatment; NIN: nintedanib; Target: Target therapy; Target+Che:Target therapy plus chemotherapy; TCM+BEV+Che: traditional Chinese medicine combined with bevacizumab plus chemotherapy; PAN: panitumumab; PAN+Che: panitumumab plus chemotherapy; PEM: pembrolizumab; REG: regorafenib; SIRT+Che: SIRT plus chemotherapy.

**Table S1 Comparison of Therapeutic Modalities (Short-term)**

| fixed MD | Che:ICI | Che:Int_Che | Che:SIRT_Che | Che:Target_Che | NAT:Target | NAT:Target_Che | Target:Target_Che |
| --- | --- | --- | --- | --- | --- | --- | --- |
| Mixed estimates |  |  |  |  |  |  |  |
| Che:ICI | 100 | 0 | 0 | 0 | 0 | 0 | 0 |
| Che:Int_Che | 0 | 100 | 0 | 0 | 0 | 0 | 0 |
| Che:SIRT_Che | 0 | 0 | 100 | 0 | 0 | 0 | 0 |
| Che:Target_Che | 0 | 0 | 0 | 100 | 0 | 0 | 0 |
| NAT:Target | 0 | 0 | 0 | 0 | 84.87 | 7.565 | 7.565 |
| NAT:Target_Che | 0 | 0 | 0 | 0 | 30.335 | 39.33 | 30.335 |
| Target:Target_Che | 0 | 0 | 0 | 0 | 12.1 | 12.1 | 75.8 |
| Indirect estimates |  |  |  |  |  |  |  |
| Che:NAT | 0 | 0 | 0 | 39.8883 | 20.2233 | 19.665 | 20.2233 |
| Che:Target | 0 | 0 | 0 | 45.9667 | 8.0667 | 8.0667 | 37.9 |
| ICI:Int_Che | 50 | 50 | 0 | 0 | 0 | 0 | 0 |
| ICI:NAT | 28.2775 | 0 | 0 | 28.2775 | 15.1675 | 13.11 | 15.1675 |
| ICI:SIRT_Che | 50 | 0 | 50 | 0 | 0 | 0 | 0 |
| ICI:Target | 31.3167 | 0 | 0 | 31.3167 | 6.05 | 6.05 | 25.2667 |
| ICI:Target_Che | 50 | 0 | 0 | 50 | 0 | 0 | 0 |
| Int_Che:NAT | 0 | 28.2775 | 0 | 28.2775 | 15.1675 | 13.11 | 15.1675 |
| Int_Che:SIRT_Che | 0 | 50 | 50 | 0 | 0 | 0 | 0 |
| Int_Che:Target | 0 | 31.3167 | 0 | 31.3167 | 6.05 | 6.05 | 25.2667 |
| Int_Che:Target_Che | 0 | 50 | 0 | 50 | 0 | 0 | 0 |
| NAT:SIRT_Che | 0 | 0 | 28.2775 | 28.2775 | 15.1675 | 13.11 | 15.1675 |
| SIRT_Che:Target | 0 | 0 | 31.3167 | 31.3167 | 6.05 | 6.05 | 25.2667 |
| SIRT_Che:Target_Che | 0 | 0 | 50 | 50 | 0 | 0 | 0 |

**Table S2 Comparison of Therapeutic Modalities (Long-term)**

| fixed MD | Che:ICI | Che:Int_Che | Che:SIRT_Che | Che:Target_Che | NAT:Target | NAT:Target_Che | Target_Che:TCM_Target_Che | Target:Target_Che |
| --- | --- | --- | --- | --- | --- | --- | --- | --- |
| Mixed estimates |  |  |  |  |  |  |  |  |
| Che:ICI | 100 | 0 | 0 | 0 | 0 | 0 | 0 | 0 |
| Che:Int_Che | 0 | 100 | 0 | 0 | 0 | 0 | 0 | 0 |
| Che:SIRT_Che | 0 | 0 | 100 | 0 | 0 | 0 | 0 | 0 |
| Che:Target_Che | 0 | 0 | 0 | 100 | 0 | 0 | 0 | 0 |
| NAT:Target | 0 | 0 | 0 | 0 | 96.27 | 1.865 | 0 | 1.865 |
| NAT:Target_Che | 0 | 0 | 0 | 0 | 37.035 | 25.93 | 0 | 37.035 |
| Target:Target_Che | 0 | 0 | 0 | 0 | 11.1 | 11.1 | 0 | 77.8 |
| Target_Che:TCM_Target_Che | 0 | 0 | 0 | 0 | 0 | 0 | 100 | 0 |
| Indirect estimates |  |  |  |  |  |  |  |  |
| Che:NAT | 0 | 0 | 0 | 37.655 | 24.69 | 12.965 | 0 | 24.69 |
| Che:TCM_Target_Che | 0 | 0 | 0 | 50 | 0 | 0 | 50 | 0 |
| Che:Target | 0 | 0 | 0 | 46.3 | 7.4 | 7.4 | 0 | 38.9 |
| ICI:Int_Che | 50 | 50 | 0 | 0 | 0 | 0 | 0 | 0 |
| ICI:NAT | 27.1608 | 0 | 0 | 27.1608 | 18.5175 | 8.6433 | 0 | 18.5175 |
| ICI:SIRT_Che | 50 | 0 | 50 | 0 | 0 | 0 | 0 | 0 |
| ICI:TCM_Target_Che | 33.3333 | 0 | 0 | 33.3333 | 0 | 0 | 33.3333 | 0 |
| ICI:Target | 31.4833 | 0 | 0 | 31.4833 | 5.55 | 5.55 | 0 | 25.9333 |
| ICI:Target_Che | 50 | 0 | 0 | 50 | 0 | 0 | 0 | 0 |
| Int_Che:NAT | 0 | 27.1608 | 0 | 27.1608 | 18.5175 | 8.6433 | 0 | 18.5175 |
| Int_Che:SIRT_Che | 0 | 50 | 50 | 0 | 0 | 0 | 0 | 0 |
| Int_Che:TCM_Target_Che | 0 | 33.3333 | 0 | 33.3333 | 0 | 0 | 33.3333 | 0 |
| Int_Che:Target | 0 | 31.4833 | 0 | 31.4833 | 5.55 | 5.55 | 0 | 25.9333 |
| Int_Che:Target_Che | 0 | 50 | 0 | 50 | 0 | 0 | 0 | 0 |
| NAT:SIRT_Che | 0 | 0 | 27.1608 | 27.1608 | 18.5175 | 8.6433 | 0 | 18.5175 |
| NAT:TCM_Target_Che | 0 | 0 | 0 | 0 | 24.69 | 12.965 | 37.655 | 24.69 |
| SIRT_Che:TCM_Target_Che | 0 | 0 | 33.3333 | 33.3333 | 0 | 0 | 33.3333 | 0 |
| SIRT_Che:Target | 0 | 0 | 31.4833 | 31.4833 | 5.55 | 5.55 | 0 | 25.9333 |
| SIRT_Che:Target_Che | 0 | 0 | 50 | 50 | 0 | 0 | 0 | 0 |
| Target:TCM_Target_Che | 0 | 0 | 0 | 0 | 7.4 | 7.4 | 46.3 | 38.9 |

**Table S3** **Comparison of Treatment Quantities (Short-term)**

| fixed MD | combination_therapy:Monotherapy | Monotherapy:No_treatment |
| --- | --- | --- |
| Mixed estimates |  |  |
| Monotherapy:No_treatment | 0 | 100 |
| combination_therapy:Monotherapy | 100 | 0 |
| Indirect estimates |  |  |
| combination_therapy:No_treatment | 50 | 50 |

**Table S4 Table S3 Comparison of Treatment Quantities (Long-term)**

| fixed MD | combination_therapy:Monotherapy | combination_therapy:No_treatment | Monotherapy:No_treatment |
| --- | --- | --- | --- |
| Mixed estimates |  |  |  |
| Monotherapy:No_treatment | 1.97 | 1.97 | 96.06 |
| combination_therapy:Monotherapy | 82.21 | 8.895 | 8.895 |
| combination_therapy:No_treatment | 39.135 | 21.73 | 39.135 |

**Table S5 Long-term Overall Analysis for First-line Patients (Without Differentiating Chemotherapy)**

| fixed MD | cet_intermittentSC:cet_SC | cet_intermittentSC:SC | cet_SC:SC | PAN:SC_PAN | pembrolizumab:SC | SC:SC_Interferon | SC:SC_PAN | SC:SIRT_SC |
| --- | --- | --- | --- | --- | --- | --- | --- | --- |
| Mixed estimates |  |  |  |  |  |  |  |  |
| PAN:SC_PAN | 0 | 0 | 0 | 100 | 0 | 0 | 0 | 0 |
| SC:SC_Interferon | 0 | 0 | 0 | 0 | 0 | 100 | 0 | 0 |
| SC:SC_PAN | 0 | 0 | 0 | 0 | 0 | 0 | 100 | 0 |
| SC:SIRT_SC | 0 | 0 | 0 | 0 | 0 | 0 | 0 | 100 |
| cet_SC:SC | 12.125 | 12.125 | 75.75 | 0 | 0 | 0 | 0 | 0 |
| cet_intermittentSC:SC | 19.19 | 61.62 | 19.19 | 0 | 0 | 0 | 0 | 0 |
| cet_intermittentSC:cet_SC | 62.63 | 18.685 | 18.685 | 0 | 0 | 0 | 0 | 0 |
| pembrolizumab:SC | 0 | 0 | 0 | 0 | 100 | 0 | 0 | 0 |
| Indirect estimates |  |  |  |  |  |  |  |  |
| PAN:SC | 0 | 0 | 0 | 50 | 0 | 0 | 50 | 0 |
| PAN:SC_Interferon | 0 | 0 | 0 | 33.3333 | 0 | 33.3333 | 33.3333 | 0 |
| PAN:SIRT_SC | 0 | 0 | 0 | 33.3333 | 0 | 0 | 33.3333 | 33.3333 |
| PAN:pembrolizumab | 0 | 0 | 0 | 33.3333 | 33.3333 | 0 | 33.3333 | 0 |
| SC_Interferon:SC_PAN | 0 | 0 | 0 | 0 | 0 | 50 | 50 | 0 |
| SC_Interferon:SIRT_SC | 0 | 0 | 0 | 0 | 0 | 50 | 0 | 50 |
| SC_PAN:SIRT_SC | 0 | 0 | 0 | 0 | 0 | 0 | 50 | 50 |
| cet_SC:PAN | 6.0625 | 6.0625 | 25.25 | 31.3125 | 0 | 0 | 31.3125 | 0 |
| cet_SC:SC_Interferon | 8.0833 | 8.0833 | 37.875 | 0 | 0 | 45.9583 | 0 | 0 |
| cet_SC:SC_PAN | 8.0833 | 8.0833 | 37.875 | 0 | 0 | 0 | 45.9583 | 0 |
| cet_SC:SIRT_SC | 8.0833 | 8.0833 | 37.875 | 0 | 0 | 0 | 0 | 45.9583 |
| cet_SC:pembrolizumab | 8.0833 | 8.0833 | 37.875 | 0 | 45.9583 | 0 | 0 | 0 |
| cet_intermittentSC:PAN | 9.595 | 20.54 | 9.595 | 30.135 | 0 | 0 | 30.135 | 0 |
| cet_intermittentSC:SC_Interferon | 12.7933 | 30.81 | 12.7933 | 0 | 0 | 43.6033 | 0 | 0 |
| cet_intermittentSC:SC_PAN | 12.7933 | 30.81 | 12.7933 | 0 | 0 | 0 | 43.6033 | 0 |
| cet_intermittentSC:SIRT_SC | 12.7933 | 30.81 | 12.7933 | 0 | 0 | 0 | 0 | 43.6033 |
| cet_intermittentSC:pembrolizumab | 12.7933 | 30.81 | 12.7933 | 0 | 43.6033 | 0 | 0 | 0 |
| pembrolizumab:SC_Interferon | 0 | 0 | 0 | 0 | 50 | 50 | 0 | 0 |
| pembrolizumab:SC_PAN | 0 | 0 | 0 | 0 | 50 | 0 | 50 | 0 |
| pembrolizumab:SIRT_SC | 0 | 0 | 0 | 0 | 50 | 0 | 0 | 50 |

**Table S6 Long-term Overall Analysis for Subsequent-line Patients (Without Differentiating Chemotherapy)**

| fixed MD | anlotinib:No_active_treatment | BEV:BEV_plus_Che | BEV:No_active_treatment | BEV_plus_Che:CET_plus_Che | BEV_plus_Che:No_active_treatment | CET:No_active_treatment | CET_plus_Che:Che | CET_plus_Che:enc_plus_CET | CET_plus_Che:enc_plus_CET_plus_bin | Che:Pan_Che | enc_plus_CET:enc_plus_CET_plus_bin | famitinib:No_active_treatment | nintedanib:No_active_treatment | No_active_treatment:regorafenib |
| --- | --- | --- | --- | --- | --- | --- | --- | --- | --- | --- | --- | --- | --- | --- |
| Mixed estimates |  |  |  |  |  |  |  |  |  |  |  |  |  |  |
| BEV:BEV_plus_Che | 0 | 60.82 | 19.59 | 0 | 19.59 | 0 | 0 | 0 | 0 | 0 | 0 | 0 | 0 | 0 |
| BEV:No_active_treatment | 0 | 15.585 | 68.83 | 0 | 15.585 | 0 | 0 | 0 | 0 | 0 | 0 | 0 | 0 | 0 |
| BEV_plus_Che:CET_plus_Che | 0 | 0 | 0 | 100 | 0 | 0 | 0 | 0 | 0 | 0 | 0 | 0 | 0 | 0 |
| BEV_plus_Che:No_active_treatment | 0 | 14.825 | 14.825 | 0 | 70.35 | 0 | 0 | 0 | 0 | 0 | 0 | 0 | 0 | 0 |
| CET:No_active_treatment | 0 | 0 | 0 | 0 | 0 | 100 | 0 | 0 | 0 | 0 | 0 | 0 | 0 | 0 |
| CET_plus_Che:Che | 0 | 0 | 0 | 0 | 0 | 0 | 100 | 0 | 0 | 0 | 0 | 0 | 0 | 0 |
| CET_plus_Che:enc_plus_CET | 0 | 0 | 0 | 0 | 0 | 0 | 0 | 59.94 | 20.03 | 0 | 20.03 | 0 | 0 | 0 |
| CET_plus_Che:enc_plus_CET_plus_bin | 0 | 0 | 0 | 0 | 0 | 0 | 0 | 19.685 | 60.63 | 0 | 19.685 | 0 | 0 | 0 |
| Che:Pan_Che | 0 | 0 | 0 | 0 | 0 | 0 | 0 | 0 | 0 | 100 | 0 | 0 | 0 | 0 |
| No_active_treatment:regorafenib | 0 | 0 | 0 | 0 | 0 | 0 | 0 | 0 | 0 | 0 | 0 | 0 | 0 | 100 |
| anlotinib:No_active_treatment | 100 | 0 | 0 | 0 | 0 | 0 | 0 | 0 | 0 | 0 | 0 | 0 | 0 | 0 |
| enc_plus_CET:enc_plus_CET_plus_bin | 0 | 0 | 0 | 0 | 0 | 0 | 0 | 10.285 | 10.285 | 0 | 79.43 | 0 | 0 | 0 |
| famitinib:No_active_treatment | 0 | 0 | 0 | 0 | 0 | 0 | 0 | 0 | 0 | 0 | 0 | 100 | 0 | 0 |
| nintedanib:No_active_treatment | 0 | 0 | 0 | 0 | 0 | 0 | 0 | 0 | 0 | 0 | 0 | 0 | 100 | 0 |
| Indirect estimates |  |  |  |  |  |  |  |  |  |  |  |  |  |  |
| BEV:CET | 0 | 10.39 | 34.415 | 0 | 10.39 | 44.805 | 0 | 0 | 0 | 0 | 0 | 0 | 0 | 0 |
| BEV:CET_plus_Che | 0 | 30.41 | 13.06 | 43.47 | 13.06 | 0 | 0 | 0 | 0 | 0 | 0 | 0 | 0 | 0 |
| BEV:Che | 0 | 20.2733 | 9.795 | 30.0683 | 9.795 | 0 | 30.0683 | 0 | 0 | 0 | 0 | 0 | 0 | 0 |
| BEV:Pan_Che | 0 | 15.205 | 7.836 | 23.041 | 7.836 | 0 | 23.041 | 0 | 0 | 23.041 | 0 | 0 | 0 | 0 |
| BEV:enc_plus_CET | 0 | 20.2 | 7.836 | 28.036 | 7.836 | 0 | 0 | 19.98 | 8.056 | 0 | 8.056 | 0 | 0 | 0 |
| BEV:enc_plus_CET_plus_bin | 0 | 20.2575 | 7.836 | 28.0935 | 7.836 | 0 | 0 | 7.8835 | 20.21 | 0 | 7.8835 | 0 | 0 | 0 |
| BEV:famitinib | 0 | 10.39 | 34.415 | 0 | 10.39 | 0 | 0 | 0 | 0 | 0 | 0 | 44.805 | 0 | 0 |
| BEV:nintedanib | 0 | 10.39 | 34.415 | 0 | 10.39 | 0 | 0 | 0 | 0 | 0 | 0 | 0 | 44.805 | 0 |
| BEV:regorafenib | 0 | 10.39 | 34.415 | 0 | 10.39 | 0 | 0 | 0 | 0 | 0 | 0 | 0 | 0 | 44.805 |
| BEV_plus_Che:CET | 0 | 9.8833 | 9.8833 | 0 | 35.175 | 45.0583 | 0 | 0 | 0 | 0 | 0 | 0 | 0 | 0 |
| BEV_plus_Che:Che | 0 | 0 | 0 | 50 | 0 | 0 | 50 | 0 | 0 | 0 | 0 | 0 | 0 | 0 |
| BEV_plus_Che:Pan_Che | 0 | 0 | 0 | 33.3333 | 0 | 0 | 33.3333 | 0 | 0 | 33.3333 | 0 | 0 | 0 | 0 |
| BEV_plus_Che:enc_plus_CET | 0 | 0 | 0 | 43.3233 | 0 | 0 | 0 | 29.97 | 13.3533 | 0 | 13.3533 | 0 | 0 | 0 |
| BEV_plus_Che:enc_plus_CET_plus_bin | 0 | 0 | 0 | 43.4383 | 0 | 0 | 0 | 13.1233 | 30.315 | 0 | 13.1233 | 0 | 0 | 0 |
| BEV_plus_Che:famitinib | 0 | 9.8833 | 9.8833 | 0 | 35.175 | 0 | 0 | 0 | 0 | 0 | 0 | 45.0583 | 0 | 0 |
| BEV_plus_Che:nintedanib | 0 | 9.8833 | 9.8833 | 0 | 35.175 | 0 | 0 | 0 | 0 | 0 | 0 | 0 | 45.0583 | 0 |
| BEV_plus_Che:regorafenib | 0 | 9.8833 | 9.8833 | 0 | 35.175 | 0 | 0 | 0 | 0 | 0 | 0 | 0 | 0 | 45.0583 |
| CET:CET_plus_Che | 0 | 7.4125 | 7.4125 | 30.8625 | 23.45 | 30.8625 | 0 | 0 | 0 | 0 | 0 | 0 | 0 | 0 |
| CET:Che | 0 | 5.93 | 5.93 | 23.5175 | 17.5875 | 23.5175 | 23.5175 | 0 | 0 | 0 | 0 | 0 | 0 | 0 |
| CET:Pan_Che | 0 | 4.9417 | 4.9417 | 19.0117 | 14.07 | 19.0117 | 19.0117 | 0 | 0 | 19.0117 | 0 | 0 | 0 | 0 |
| CET:enc_plus_CET | 0 | 4.9417 | 4.9417 | 22.0087 | 17.067 | 22.0087 | 0 | 14.985 | 7.0237 | 0 | 7.0237 | 0 | 0 | 0 |
| CET:enc_plus_CET_plus_bin | 0 | 4.9417 | 4.9417 | 22.0432 | 17.1015 | 22.0432 | 0 | 6.8857 | 15.1575 | 0 | 6.8857 | 0 | 0 | 0 |
| CET:famitinib | 0 | 0 | 0 | 0 | 0 | 50 | 0 | 0 | 0 | 0 | 0 | 50 | 0 | 0 |
| CET:nintedanib | 0 | 0 | 0 | 0 | 0 | 50 | 0 | 0 | 0 | 0 | 0 | 0 | 50 | 0 |
| CET:regorafenib | 0 | 0 | 0 | 0 | 0 | 50 | 0 | 0 | 0 | 0 | 0 | 0 | 0 | 50 |
| CET_plus_Che:No_active_treatment | 0 | 9.8833 | 9.8833 | 45.0583 | 35.175 | 0 | 0 | 0 | 0 | 0 | 0 | 0 | 0 | 0 |
| CET_plus_Che:Pan_Che | 0 | 0 | 0 | 0 | 0 | 0 | 50 | 0 | 0 | 50 | 0 | 0 | 0 | 0 |
| CET_plus_Che:famitinib | 0 | 7.4125 | 7.4125 | 30.8625 | 23.45 | 0 | 0 | 0 | 0 | 0 | 0 | 30.8625 | 0 | 0 |
| CET_plus_Che:nintedanib | 0 | 7.4125 | 7.4125 | 30.8625 | 23.45 | 0 | 0 | 0 | 0 | 0 | 0 | 0 | 30.8625 | 0 |
| CET_plus_Che:regorafenib | 0 | 7.4125 | 7.4125 | 30.8625 | 23.45 | 0 | 0 | 0 | 0 | 0 | 0 | 0 | 0 | 30.8625 |
| Che:No_active_treatment | 0 | 7.4125 | 7.4125 | 30.8625 | 23.45 | 0 | 30.8625 | 0 | 0 | 0 | 0 | 0 | 0 | 0 |
| Che:enc_plus_CET | 0 | 0 | 0 | 0 | 0 | 0 | 43.3233 | 29.97 | 13.3533 | 0 | 13.3533 | 0 | 0 | 0 |
| Che:enc_plus_CET_plus_bin | 0 | 0 | 0 | 0 | 0 | 0 | 43.4383 | 13.1233 | 30.315 | 0 | 13.1233 | 0 | 0 | 0 |
| Che:famitinib | 0 | 5.93 | 5.93 | 23.5175 | 17.5875 | 0 | 23.5175 | 0 | 0 | 0 | 0 | 23.5175 | 0 | 0 |
| Che:nintedanib | 0 | 5.93 | 5.93 | 23.5175 | 17.5875 | 0 | 23.5175 | 0 | 0 | 0 | 0 | 0 | 23.5175 | 0 |
| Che:regorafenib | 0 | 5.93 | 5.93 | 23.5175 | 17.5875 | 0 | 23.5175 | 0 | 0 | 0 | 0 | 0 | 0 | 23.5175 |
| No_active_treatment:Pan_Che | 0 | 5.93 | 5.93 | 23.5175 | 17.5875 | 0 | 23.5175 | 0 | 0 | 23.5175 | 0 | 0 | 0 | 0 |
| Pan_Che:regorafenib | 0 | 4.9417 | 4.9417 | 19.0117 | 14.07 | 0 | 19.0117 | 0 | 0 | 19.0117 | 0 | 0 | 0 | 19.0117 |
| anlotinib:BEV | 44.805 | 10.39 | 34.415 | 0 | 10.39 | 0 | 0 | 0 | 0 | 0 | 0 | 0 | 0 | 0 |
| anlotinib:BEV_plus_Che | 45.0583 | 9.8833 | 9.8833 | 0 | 35.175 | 0 | 0 | 0 | 0 | 0 | 0 | 0 | 0 | 0 |
| anlotinib:CET | 50 | 0 | 0 | 0 | 0 | 50 | 0 | 0 | 0 | 0 | 0 | 0 | 0 | 0 |
| anlotinib:CET_plus_Che | 30.8625 | 7.4125 | 7.4125 | 30.8625 | 23.45 | 0 | 0 | 0 | 0 | 0 | 0 | 0 | 0 | 0 |
| anlotinib:Che | 23.5175 | 5.93 | 5.93 | 23.5175 | 17.5875 | 0 | 23.5175 | 0 | 0 | 0 | 0 | 0 | 0 | 0 |
| anlotinib:Pan_Che | 19.0117 | 4.9417 | 4.9417 | 19.0117 | 14.07 | 0 | 19.0117 | 0 | 0 | 19.0117 | 0 | 0 | 0 | 0 |
| anlotinib:enc_plus_CET | 22.0087 | 4.9417 | 4.9417 | 22.0087 | 17.067 | 0 | 0 | 14.985 | 7.0237 | 0 | 7.0237 | 0 | 0 | 0 |
| anlotinib:enc_plus_CET_plus_bin | 22.0432 | 4.9417 | 4.9417 | 22.0432 | 17.1015 | 0 | 0 | 6.8857 | 15.1575 | 0 | 6.8857 | 0 | 0 | 0 |
| anlotinib:famitinib | 50 | 0 | 0 | 0 | 0 | 0 | 0 | 0 | 0 | 0 | 0 | 50 | 0 | 0 |
| anlotinib:nintedanib | 50 | 0 | 0 | 0 | 0 | 0 | 0 | 0 | 0 | 0 | 0 | 0 | 50 | 0 |
| anlotinib:regorafenib | 50 | 0 | 0 | 0 | 0 | 0 | 0 | 0 | 0 | 0 | 0 | 0 | 0 | 50 |
| enc_plus_CET:No_active_treatment | 0 | 5.93 | 5.93 | 28.5125 | 22.5825 | 0 | 0 | 19.98 | 8.5325 | 0 | 8.5325 | 0 | 0 | 0 |
| enc_plus_CET:Pan_Che | 0 | 0 | 0 | 0 | 0 | 0 | 29.995 | 19.98 | 10.015 | 29.995 | 10.015 | 0 | 0 | 0 |
| enc_plus_CET:famitinib | 0 | 4.9417 | 4.9417 | 22.0087 | 17.067 | 0 | 0 | 14.985 | 7.0237 | 0 | 7.0237 | 22.0087 | 0 | 0 |
| enc_plus_CET:nintedanib | 0 | 4.9417 | 4.9417 | 22.0087 | 17.067 | 0 | 0 | 14.985 | 7.0237 | 0 | 7.0237 | 0 | 22.0087 | 0 |
| enc_plus_CET:regorafenib | 0 | 4.9417 | 4.9417 | 22.0087 | 17.067 | 0 | 0 | 14.985 | 7.0237 | 0 | 7.0237 | 0 | 0 | 22.0087 |
| enc_plus_CET_plus_bin:No_active_treatment | 0 | 5.93 | 5.93 | 28.57 | 22.64 | 0 | 0 | 8.36 | 20.21 | 0 | 8.36 | 0 | 0 | 0 |
| enc_plus_CET_plus_bin:Pan_Che | 0 | 0 | 0 | 0 | 0 | 0 | 30.0525 | 9.8425 | 20.21 | 30.0525 | 9.8425 | 0 | 0 | 0 |
| enc_plus_CET_plus_bin:famitinib | 0 | 4.9417 | 4.9417 | 22.0432 | 17.1015 | 0 | 0 | 6.8857 | 15.1575 | 0 | 6.8857 | 22.0432 | 0 | 0 |
| enc_plus_CET_plus_bin:nintedanib | 0 | 4.9417 | 4.9417 | 22.0432 | 17.1015 | 0 | 0 | 6.8857 | 15.1575 | 0 | 6.8857 | 0 | 22.0432 | 0 |
| enc_plus_CET_plus_bin:regorafenib | 0 | 4.9417 | 4.9417 | 22.0432 | 17.1015 | 0 | 0 | 6.8857 | 15.1575 | 0 | 6.8857 | 0 | 0 | 22.0432 |
| famitinib:Pan_Che | 0 | 4.9417 | 4.9417 | 19.0117 | 14.07 | 0 | 19.0117 | 0 | 0 | 19.0117 | 0 | 19.0117 | 0 | 0 |
| famitinib:nintedanib | 0 | 0 | 0 | 0 | 0 | 0 | 0 | 0 | 0 | 0 | 0 | 50 | 50 | 0 |
| famitinib:regorafenib | 0 | 0 | 0 | 0 | 0 | 0 | 0 | 0 | 0 | 0 | 0 | 50 | 0 | 50 |
| nintedanib:Pan_Che | 0 | 4.9417 | 4.9417 | 19.0117 | 14.07 | 0 | 19.0117 | 0 | 0 | 19.0117 | 0 | 0 | 19.0117 | 0 |
| nintedanib:regorafenib | 0 | 0 | 0 | 0 | 0 | 0 | 0 | 0 | 0 | 0 | 0 | 0 | 50 | 50 |

**Table S7 Short-term Overall Analysis for First-line Patients (Without Differentiating Chemotherapy)**

| fixed MD | cet_plus_Che:cet_plus_intermittentChe | cet_plus_Che:Che | cet_plus_intermittentChe:Che | Che:Interferon_plus_Che | Che:panitumumab_plus_Che | Che:pembrolizumab | Che:SIRT_plus_Che | panitumumab:panitumumab_plus_Che |
| --- | --- | --- | --- | --- | --- | --- | --- | --- |
| Mixed estimates |  |  |  |  |  |  |  |  |
| Che:Interferon_plus_Che | 0 | 0 | 0 | 100 | 0 | 0 | 0 | 0 |
| Che:SIRT_plus_Che | 0 | 0 | 0 | 0 | 0 | 0 | 100 | 0 |
| Che:panitumumab_plus_Che | 0 | 0 | 0 | 0 | 100 | 0 | 0 | 0 |
| Che:pembrolizumab | 0 | 0 | 0 | 0 | 0 | 100 | 0 | 0 |
| cet_plus_Che:Che | 11.74 | 76.52 | 11.74 | 0 | 0 | 0 | 0 | 0 |
| cet_plus_Che:cet_plus_intermittentChe | 60.89 | 19.555 | 19.555 | 0 | 0 | 0 | 0 | 0 |
| cet_plus_intermittentChe:Che | 18.7 | 18.7 | 62.6 | 0 | 0 | 0 | 0 | 0 |
| panitumumab:panitumumab_plus_Che | 0 | 0 | 0 | 0 | 0 | 0 | 0 | 100 |
| Indirect estimates |  |  |  |  |  |  |  |  |
| Che:panitumumab | 0 | 0 | 0 | 0 | 50 | 0 | 0 | 50 |
| Interferon_plus_Che:SIRT_plus_Che | 0 | 0 | 0 | 50 | 0 | 0 | 50 | 0 |
| Interferon_plus_Che:panitumumab | 0 | 0 | 0 | 33.3333 | 33.3333 | 0 | 0 | 33.3333 |
| Interferon_plus_Che:panitumumab_plus_Che | 0 | 0 | 0 | 50 | 50 | 0 | 0 | 0 |
| Interferon_plus_Che:pembrolizumab | 0 | 0 | 0 | 50 | 0 | 50 | 0 | 0 |
| cet_plus_Che:Interferon_plus_Che | 7.8267 | 38.26 | 7.8267 | 46.0867 | 0 | 0 | 0 | 0 |
| cet_plus_Che:SIRT_plus_Che | 7.8267 | 38.26 | 7.8267 | 0 | 0 | 0 | 46.0867 | 0 |
| cet_plus_Che:panitumumab | 5.87 | 25.5067 | 5.87 | 0 | 31.3767 | 0 | 0 | 31.3767 |
| cet_plus_Che:panitumumab_plus_Che | 7.8267 | 38.26 | 7.8267 | 0 | 46.0867 | 0 | 0 | 0 |
| cet_plus_Che:pembrolizumab | 7.8267 | 38.26 | 7.8267 | 0 | 0 | 46.0867 | 0 | 0 |
| cet_plus_intermittentChe:Interferon_plus_Che | 12.4667 | 12.4667 | 31.3 | 43.7667 | 0 | 0 | 0 | 0 |
| cet_plus_intermittentChe:SIRT_plus_Che | 12.4667 | 12.4667 | 31.3 | 0 | 0 | 0 | 43.7667 | 0 |
| cet_plus_intermittentChe:panitumumab | 9.35 | 9.35 | 20.8667 | 0 | 30.2167 | 0 | 0 | 30.2167 |
| cet_plus_intermittentChe:panitumumab_plus_Che | 12.4667 | 12.4667 | 31.3 | 0 | 43.7667 | 0 | 0 | 0 |
| cet_plus_intermittentChe:pembrolizumab | 12.4667 | 12.4667 | 31.3 | 0 | 0 | 43.7667 | 0 | 0 |
| panitumumab:SIRT_plus_Che | 0 | 0 | 0 | 0 | 33.3333 | 0 | 33.3333 | 33.3333 |
| panitumumab:pembrolizumab | 0 | 0 | 0 | 0 | 33.3333 | 33.3333 | 0 | 33.3333 |
| panitumumab_plus_Che:SIRT_plus_Che | 0 | 0 | 0 | 0 | 50 | 0 | 50 | 0 |
| panitumumab_plus_Che:pembrolizumab | 0 | 0 | 0 | 0 | 50 | 50 | 0 | 0 |
| pembrolizumab:SIRT_plus_Che | 0 | 0 | 0 | 0 | 0 | 50 | 50 | 0 |

**Table S8 Short-term Overall Analysis for Subsequent-line Patients (Without Differentiating Chemotherapy)**

| fixed MD | anlotinib:NAT | bev:bev_plus_Che | bev:NAT | bev_plus_Che:cet_plus_Che | bev_plus_Che:NAT | cet:NAT | cet_plus_Che:Che | cet_plus_Che:encorafenib_plus_cet | cet_plus_Che:encorafenib_plus_cet_plus_binimetinib | encorafenib_plus_cet:encorafenib_plus_cet_plus_binimetinib | famitinib:NAT |
| --- | --- | --- | --- | --- | --- | --- | --- | --- | --- | --- | --- |
| Mixed estimates |  |  |  |  |  |  |  |  |  |  |  |
| anlotinib:NAT | 100 | 0 | 0 | 0 | 0 | 0 | 0 | 0 | 0 | 0 | 0 |
| bev:NAT | 0 | 16.215 | 67.57 | 0 | 16.215 | 0 | 0 | 0 | 0 | 0 | 0 |
| bev:bev_plus_Che | 0 | 65.28 | 17.36 | 0 | 17.36 | 0 | 0 | 0 | 0 | 0 | 0 |
| bev_plus_Che:NAT | 0 | 16.42 | 16.42 | 0 | 67.16 | 0 | 0 | 0 | 0 | 0 | 0 |
| bev_plus_Che:cet_plus_Che | 0 | 0 | 0 | 100 | 0 | 0 | 0 | 0 | 0 | 0 | 0 |
| cet:NAT | 0 | 0 | 0 | 0 | 0 | 100 | 0 | 0 | 0 | 0 | 0 |
| cet_plus_Che:Che | 0 | 0 | 0 | 0 | 0 | 0 | 100 | 0 | 0 | 0 | 0 |
| cet_plus_Che:encorafenib_plus_cet | 0 | 0 | 0 | 0 | 0 | 0 | 0 | 58.64 | 20.68 | 20.68 | 0 |
| cet_plus_Che:encorafenib_plus_cet_plus_binimetinib | 0 | 0 | 0 | 0 | 0 | 0 | 0 | 20.02 | 59.96 | 20.02 | 0 |
| encorafenib_plus_cet:encorafenib_plus_cet_plus_binimetinib | 0 | 0 | 0 | 0 | 0 | 0 | 0 | 9.3 | 9.3 | 81.4 | 0 |
| famitinib:NAT | 0 | 0 | 0 | 0 | 0 | 0 | 0 | 0 | 0 | 0 | 100 |
| Indirect estimates |  |  |  |  |  |  |  |  |  |  |  |
| Che:NAT | 0 | 8.21 | 8.21 | 30.5967 | 22.3867 | 0 | 30.5967 | 0 | 0 | 0 | 0 |
| Che:encorafenib_plus_cet | 0 | 0 | 0 | 0 | 0 | 0 | 43.1067 | 29.32 | 13.7867 | 13.7867 | 0 |
| Che:encorafenib_plus_cet_plus_binimetinib | 0 | 0 | 0 | 0 | 0 | 0 | 43.3267 | 13.3467 | 29.98 | 13.3467 | 0 |
| Che:famitinib | 0 | 6.568 | 6.568 | 23.358 | 16.79 | 0 | 23.358 | 0 | 0 | 0 | 23.358 |
| anlotinib:Che | 23.358 | 6.568 | 6.568 | 23.358 | 16.79 | 0 | 23.358 | 0 | 0 | 0 | 0 |
| anlotinib:bev | 44.595 | 10.81 | 33.785 | 0 | 10.81 | 0 | 0 | 0 | 0 | 0 | 0 |
| anlotinib:bev_plus_Che | 44.5267 | 10.9467 | 10.9467 | 0 | 33.58 | 0 | 0 | 0 | 0 | 0 | 0 |
| anlotinib:cet | 50 | 0 | 0 | 0 | 0 | 50 | 0 | 0 | 0 | 0 | 0 |
| anlotinib:cet_plus_Che | 30.5967 | 8.21 | 8.21 | 30.5967 | 22.3867 | 0 | 0 | 0 | 0 | 0 | 0 |
| anlotinib:encorafenib_plus_cet | 21.8373 | 5.4733 | 5.4733 | 21.8373 | 16.364 | 0 | 0 | 14.66 | 7.1773 | 7.1773 | 0 |
| anlotinib:encorafenib_plus_cet_plus_binimetinib | 21.9033 | 5.4733 | 5.4733 | 21.9033 | 16.43 | 0 | 0 | 6.9133 | 14.99 | 6.9133 | 0 |
| anlotinib:famitinib | 50 | 0 | 0 | 0 | 0 | 0 | 0 | 0 | 0 | 0 | 50 |
| bev:Che | 0 | 21.76 | 8.68 | 30.44 | 8.68 | 0 | 30.44 | 0 | 0 | 0 | 0 |
| bev:cet | 0 | 10.81 | 33.785 | 0 | 10.81 | 44.595 | 0 | 0 | 0 | 0 | 0 |
| bev:cet_plus_Che | 0 | 32.64 | 11.5733 | 44.2133 | 11.5733 | 0 | 0 | 0 | 0 | 0 | 0 |
| bev:encorafenib_plus_cet | 0 | 21.2067 | 6.944 | 28.1507 | 6.944 | 0 | 0 | 19.5467 | 8.604 | 8.604 | 0 |
| bev:encorafenib_plus_cet_plus_binimetinib | 0 | 21.3167 | 6.944 | 28.2607 | 6.944 | 0 | 0 | 8.274 | 19.9867 | 8.274 | 0 |
| bev:famitinib | 0 | 10.81 | 33.785 | 0 | 10.81 | 0 | 0 | 0 | 0 | 0 | 44.595 |
| bev_plus_Che:Che | 0 | 0 | 0 | 50 | 0 | 0 | 50 | 0 | 0 | 0 | 0 |
| bev_plus_Che:cet | 0 | 10.9467 | 10.9467 | 0 | 33.58 | 44.5267 | 0 | 0 | 0 | 0 | 0 |
| bev_plus_Che:encorafenib_plus_cet | 0 | 0 | 0 | 43.1067 | 0 | 0 | 0 | 29.32 | 13.7867 | 13.7867 | 0 |
| bev_plus_Che:encorafenib_plus_cet_plus_binimetinib | 0 | 0 | 0 | 43.3267 | 0 | 0 | 0 | 13.3467 | 29.98 | 13.3467 | 0 |
| bev_plus_Che:famitinib | 0 | 10.9467 | 10.9467 | 0 | 33.58 | 0 | 0 | 0 | 0 | 0 | 44.5267 |
| cet:Che | 0 | 6.568 | 6.568 | 23.358 | 16.79 | 23.358 | 23.358 | 0 | 0 | 0 | 0 |
| cet:cet_plus_Che | 0 | 8.21 | 8.21 | 30.5967 | 22.3867 | 30.5967 | 0 | 0 | 0 | 0 | 0 |
| cet:encorafenib_plus_cet | 0 | 5.4733 | 5.4733 | 21.8373 | 16.364 | 21.8373 | 0 | 14.66 | 7.1773 | 7.1773 | 0 |
| cet:encorafenib_plus_cet_plus_binimetinib | 0 | 5.4733 | 5.4733 | 21.9033 | 16.43 | 21.9033 | 0 | 6.9133 | 14.99 | 6.9133 | 0 |
| cet:famitinib | 0 | 0 | 0 | 0 | 0 | 50 | 0 | 0 | 0 | 0 | 50 |
| cet_plus_Che:NAT | 0 | 10.9467 | 10.9467 | 44.5267 | 33.58 | 0 | 0 | 0 | 0 | 0 | 0 |
| cet_plus_Che:famitinib | 0 | 8.21 | 8.21 | 30.5967 | 22.3867 | 0 | 0 | 0 | 0 | 0 | 30.5967 |
| encorafenib_plus_cet:NAT | 0 | 6.568 | 6.568 | 28.2447 | 21.6767 | 0 | 0 | 19.5467 | 8.698 | 8.698 | 0 |
| encorafenib_plus_cet:famitinib | 0 | 5.4733 | 5.4733 | 21.8373 | 16.364 | 0 | 0 | 14.66 | 7.1773 | 7.1773 | 21.8373 |
| encorafenib_plus_cet_plus_binimetinib:NAT | 0 | 6.568 | 6.568 | 28.3547 | 21.7867 | 0 | 0 | 8.368 | 19.9867 | 8.368 | 0 |
| encorafenib_plus_cet_plus_binimetinib:famitinib | 0 | 5.4733 | 5.4733 | 21.9033 | 16.43 | 0 | 0 | 6.9133 | 14.99 | 6.9133 | 21.9033 |

1. **Predictive Interval Analyses**

Abbreviation ANL: anlotinib; BEV: bevacizumab; BEV+Che:bevacizumab plus chemotherapy;CET: cetuximab; CET+Che: cetuximab plus chemotherapy; CET+inter Che: cetuximab plus intermittent chemotherapy; CET+BEV+Che: cetuximab combined with bevacizumab plus chemotherapy; Che: chemotherapy; Che+INT: interferon plus chemotherapy; ENC+CET: encorafenib plus cetuximab; ENC+CET+BIN: encorafenib combined with cetuximab plus binimetinib; FAM: famitinib; ICI: Immunotherapy; NAT: No active treatment; NIN: nintedanib; Target: Target therapy; Target+Che: Target therapy plus chemotherapy; TCM+BEV+Che: traditional Chinese medicine combined with bevacizumab plus chemotherapy; PAN: panitumumab; PAN+Che: panitumumab plus chemotherapy; PEM: pembrolizumab; REG: regorafenib; SIRT+Che: SIRT plus chemotherapy.

**Fig.S1 Comparison of Therapeutic Modalities (Short-term)**


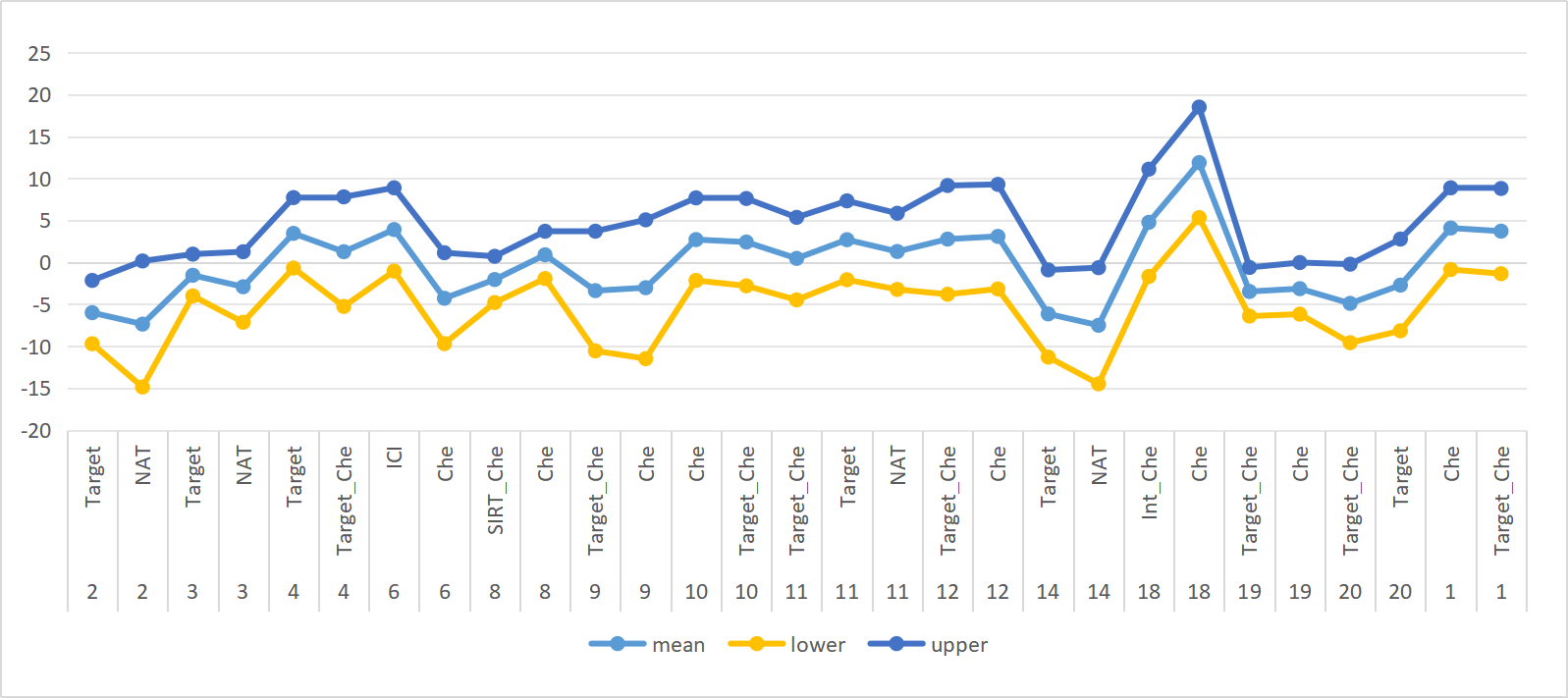


**Fig.S2 Comparison of Therapeutic Modalities (Long-term)**


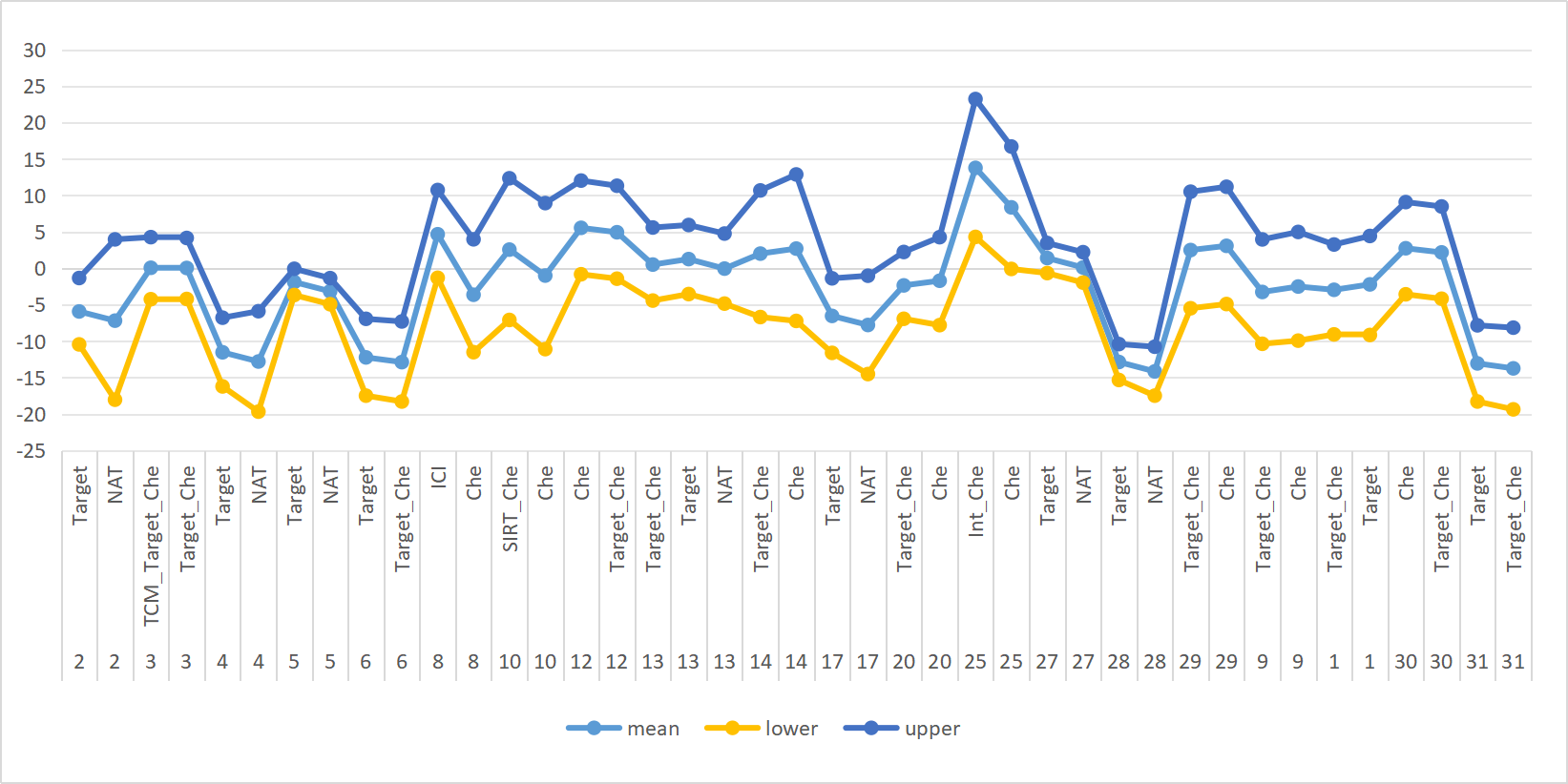


**Fig.S3 Short-term Comparison of Treatment Quantities**


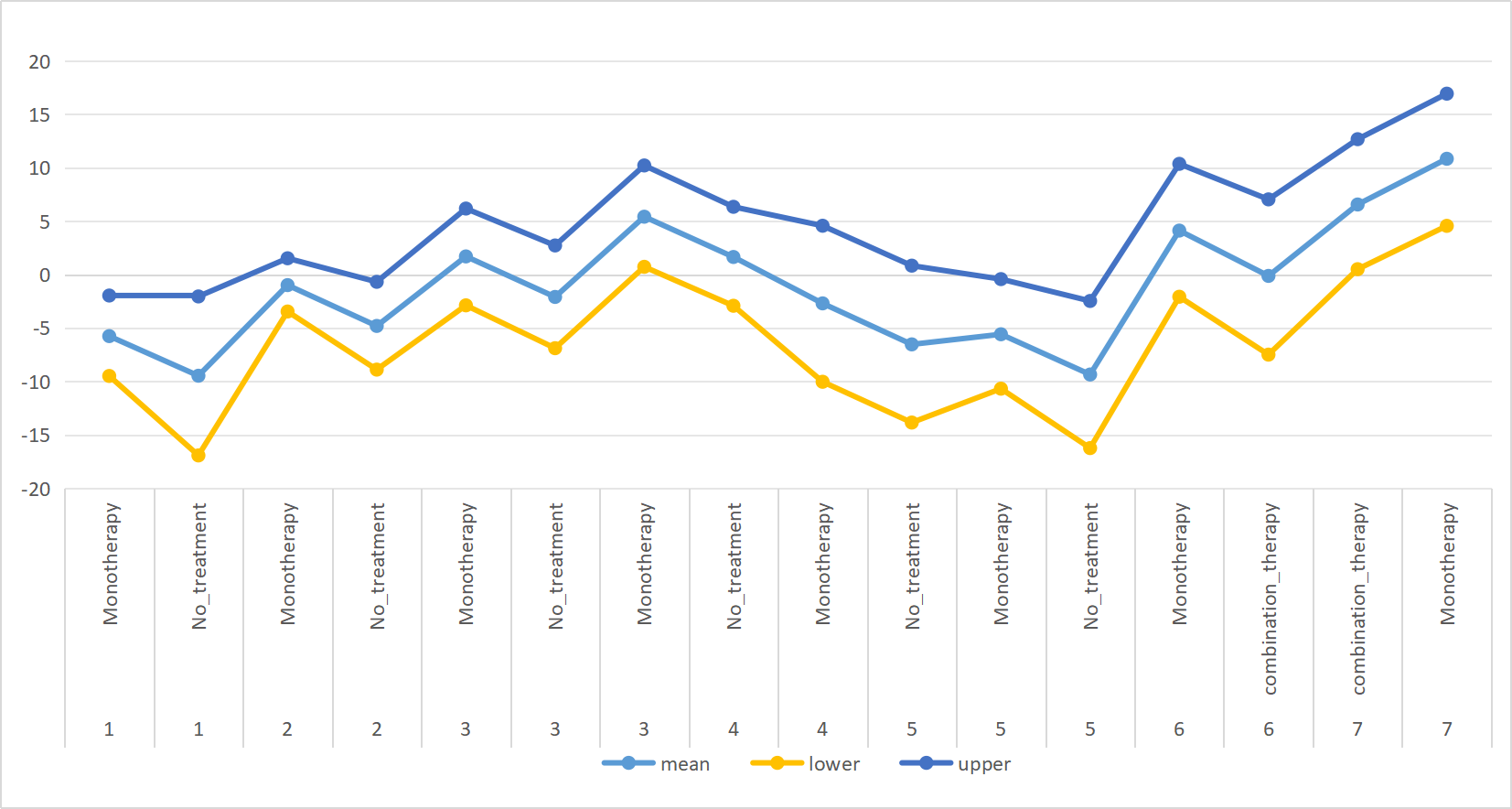


**Fig.S4 Long-term Comparison of Treatment Quantities**


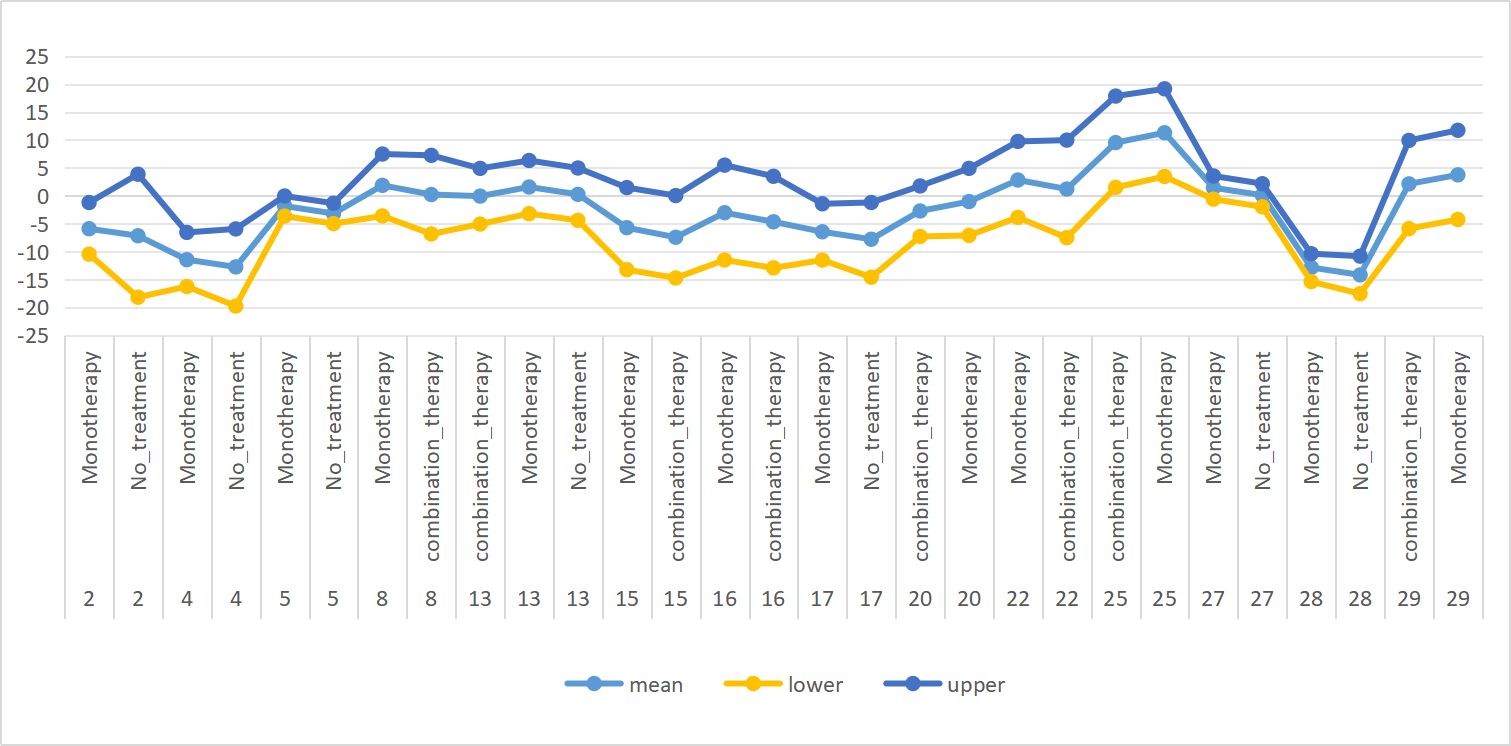


**Fig.S5 Long-term Overall Analysis for Overall Patients (Without Differentiating Chemotherapy)**


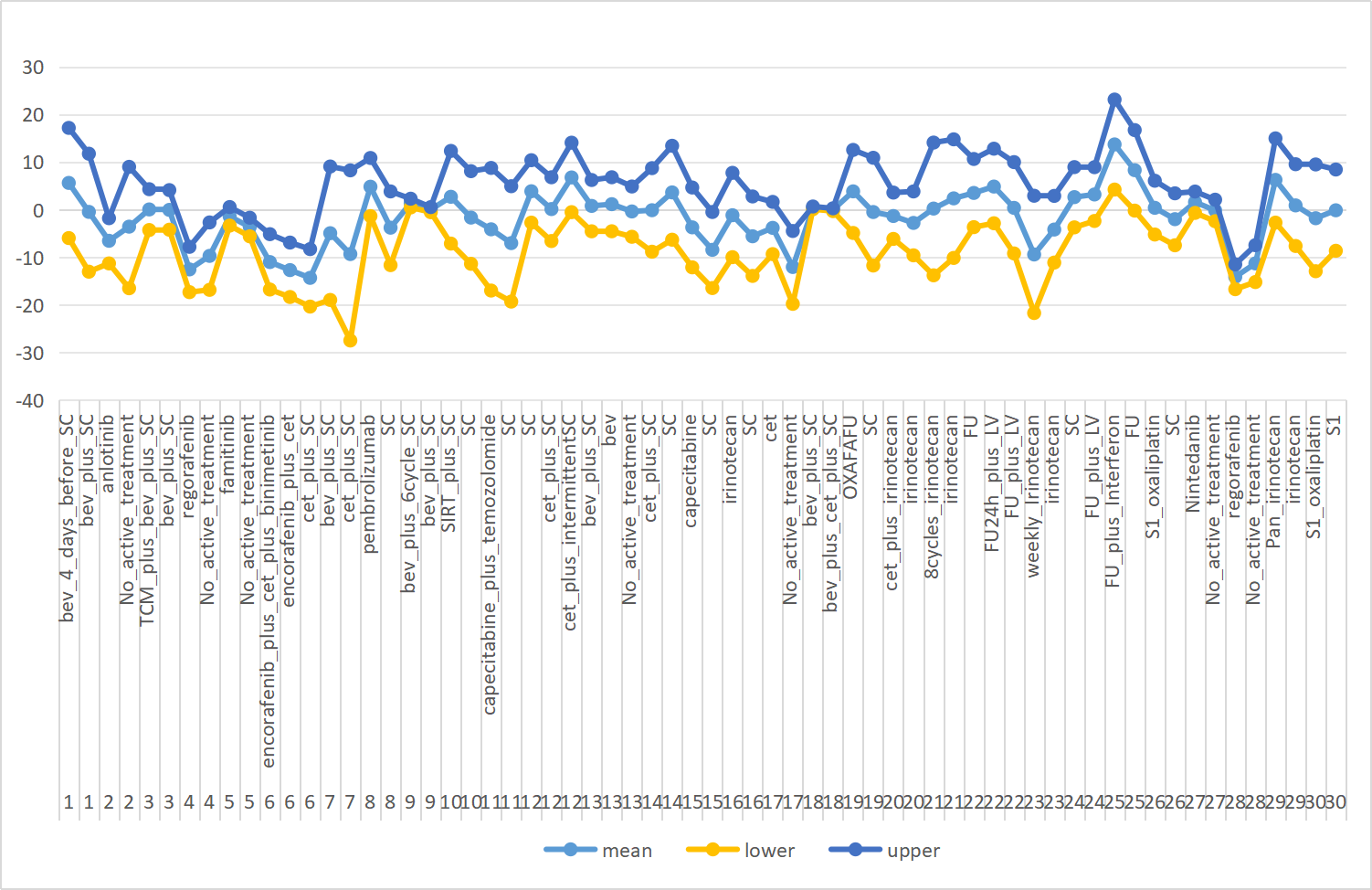


**Fig.S6 Long-term Overall Analysis for First-line Patients (Without Differentiating Chemotherapy)**


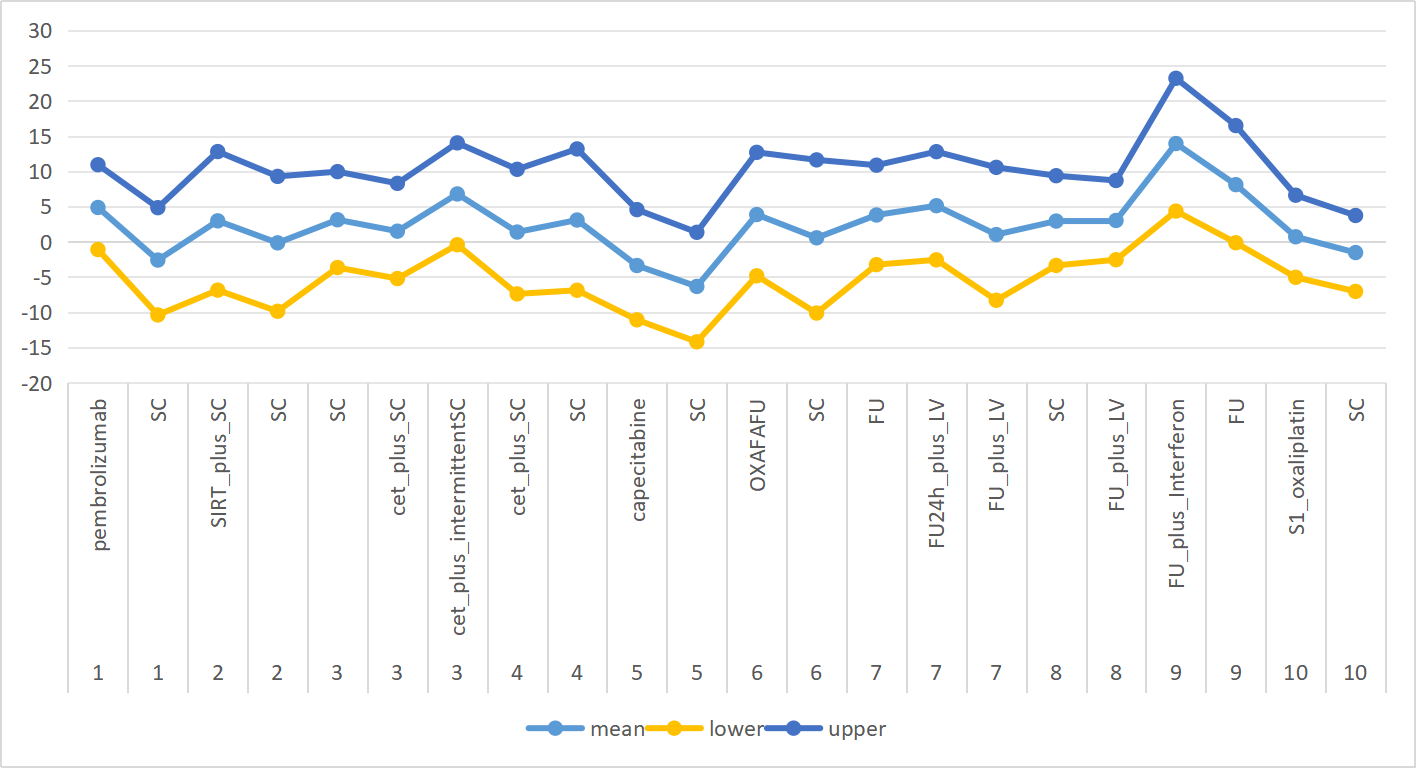


**Fig.S7 Long-term Overall Analysis for Subsequent-line Patients (Without Differentiating Chemotherapy)**


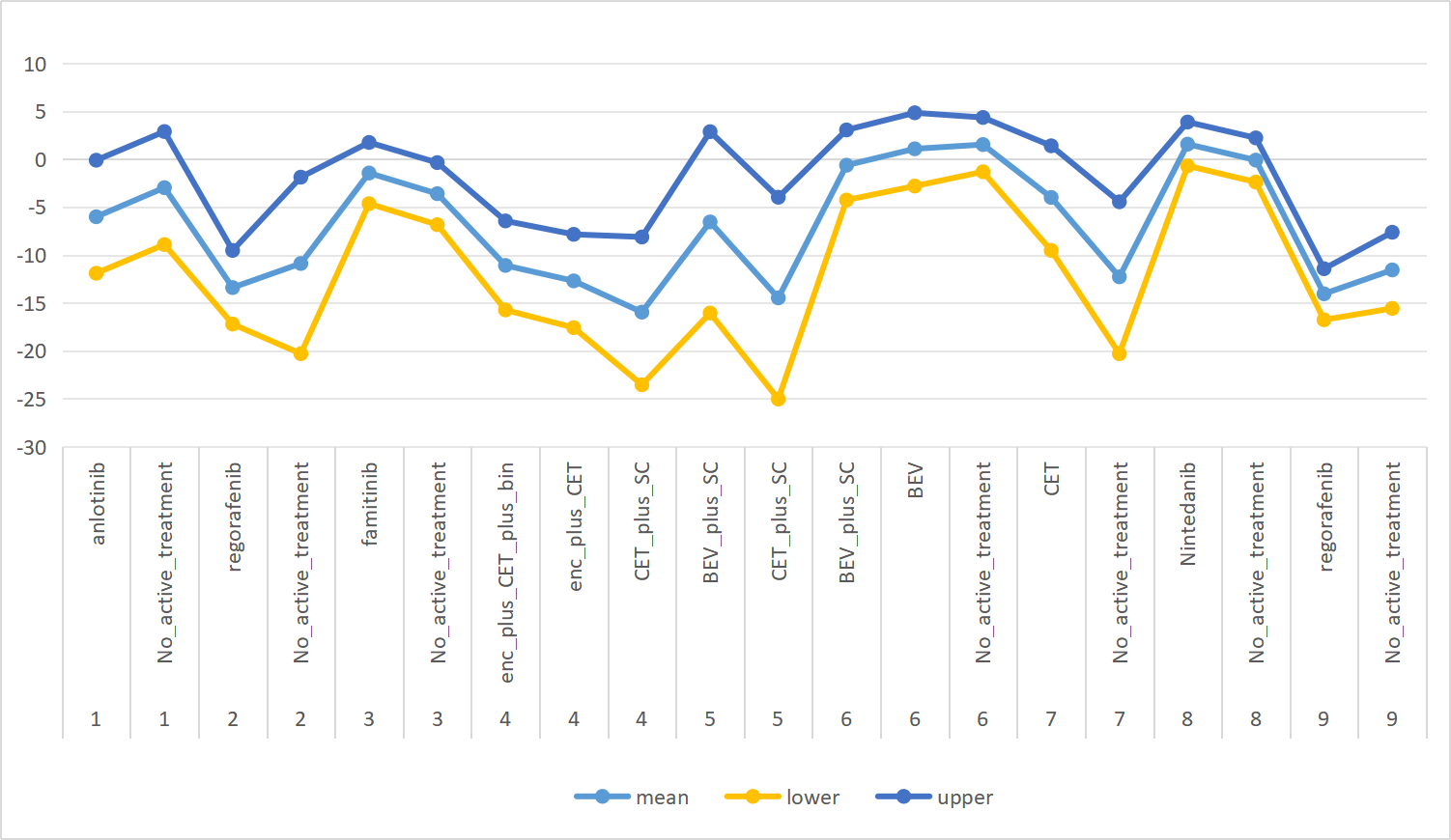


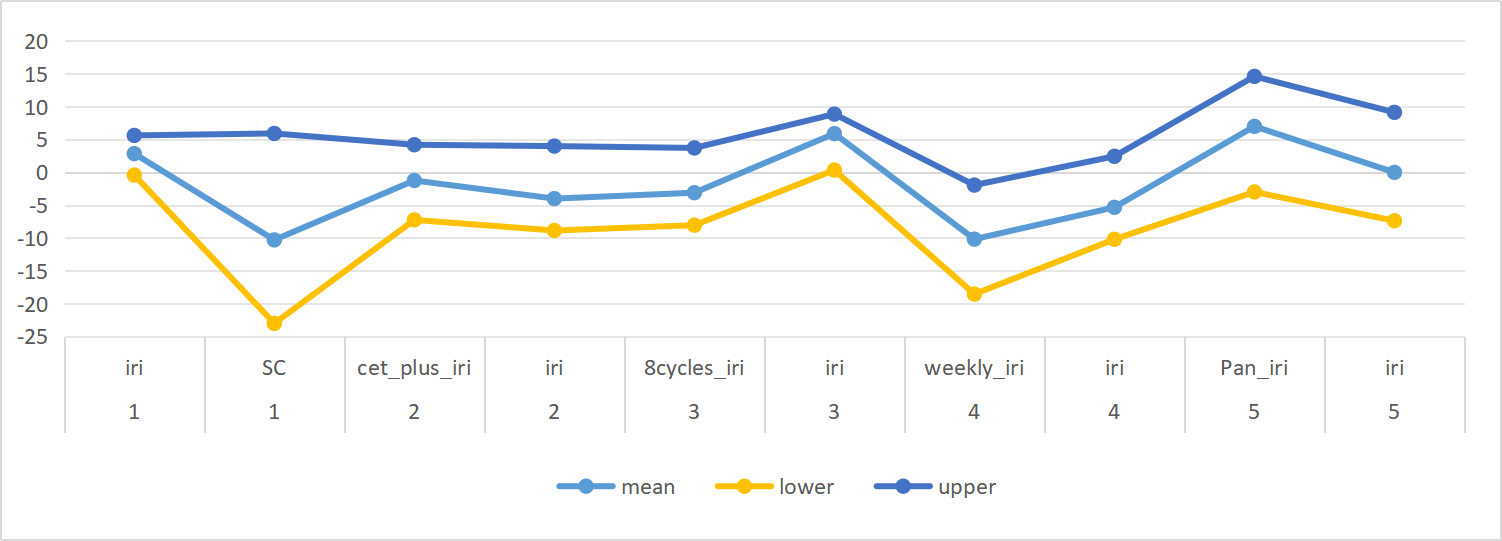


**Fig.S8 Short-term Overall Analysis for Overall Patients (Without Differentiating Chemotherapy)**


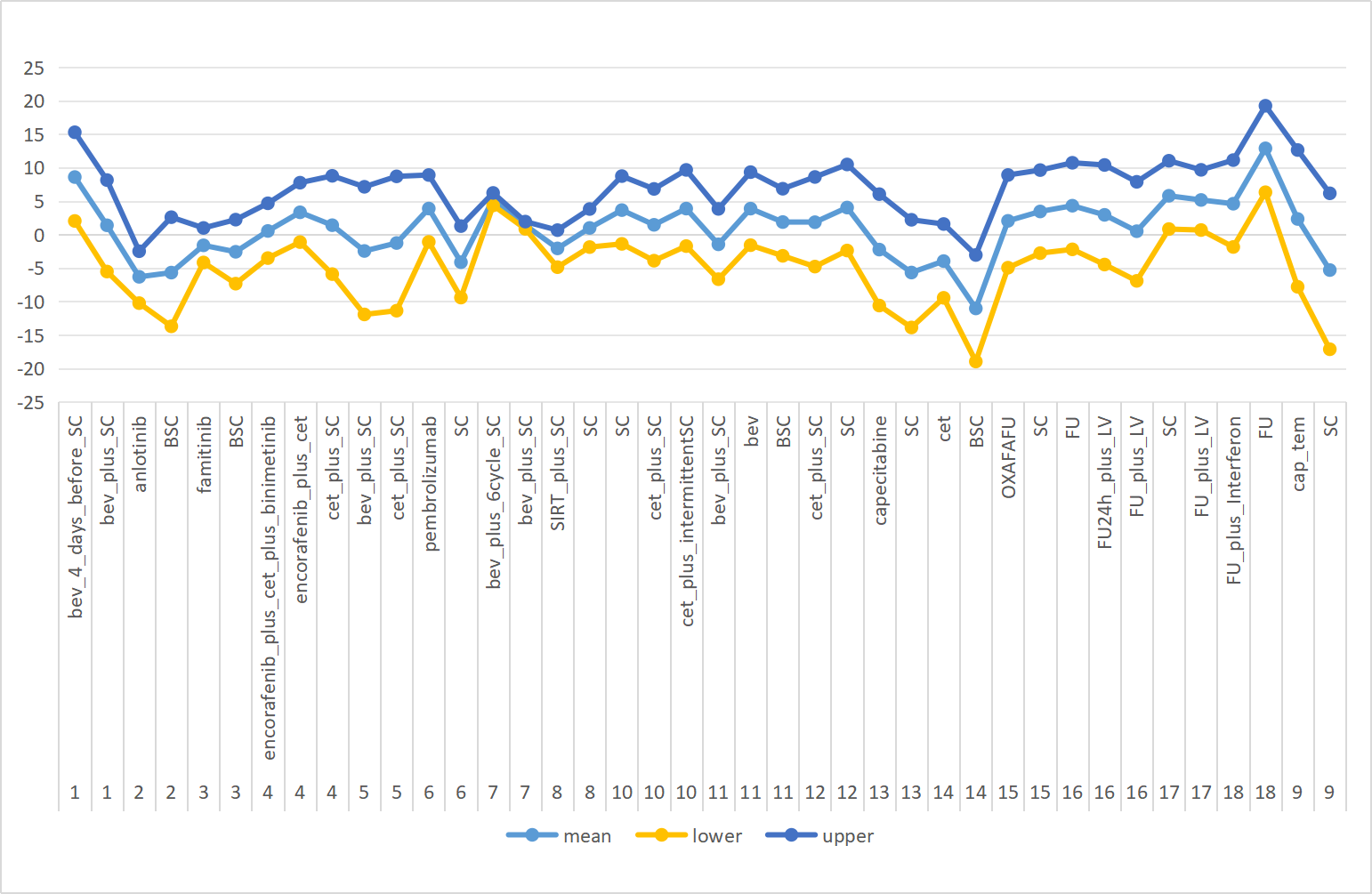


**Fig.S9 Short-term Overall Analysis for First-line Patients (Without Differentiating Chemotherapy)**


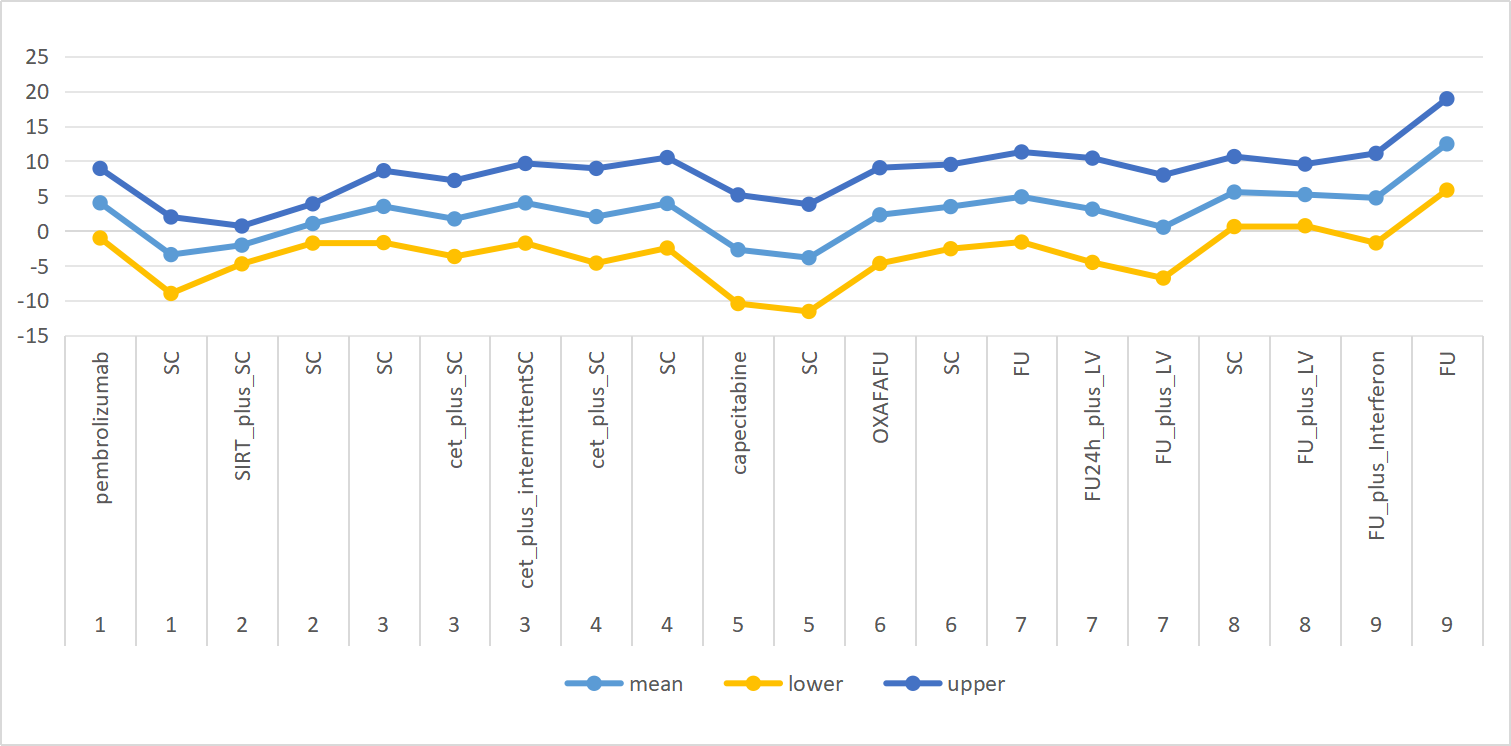


**Fig.S10 Short-term Overall Analysis for Subsequent-line Patients (Without Differentiating Chemotherapy)**


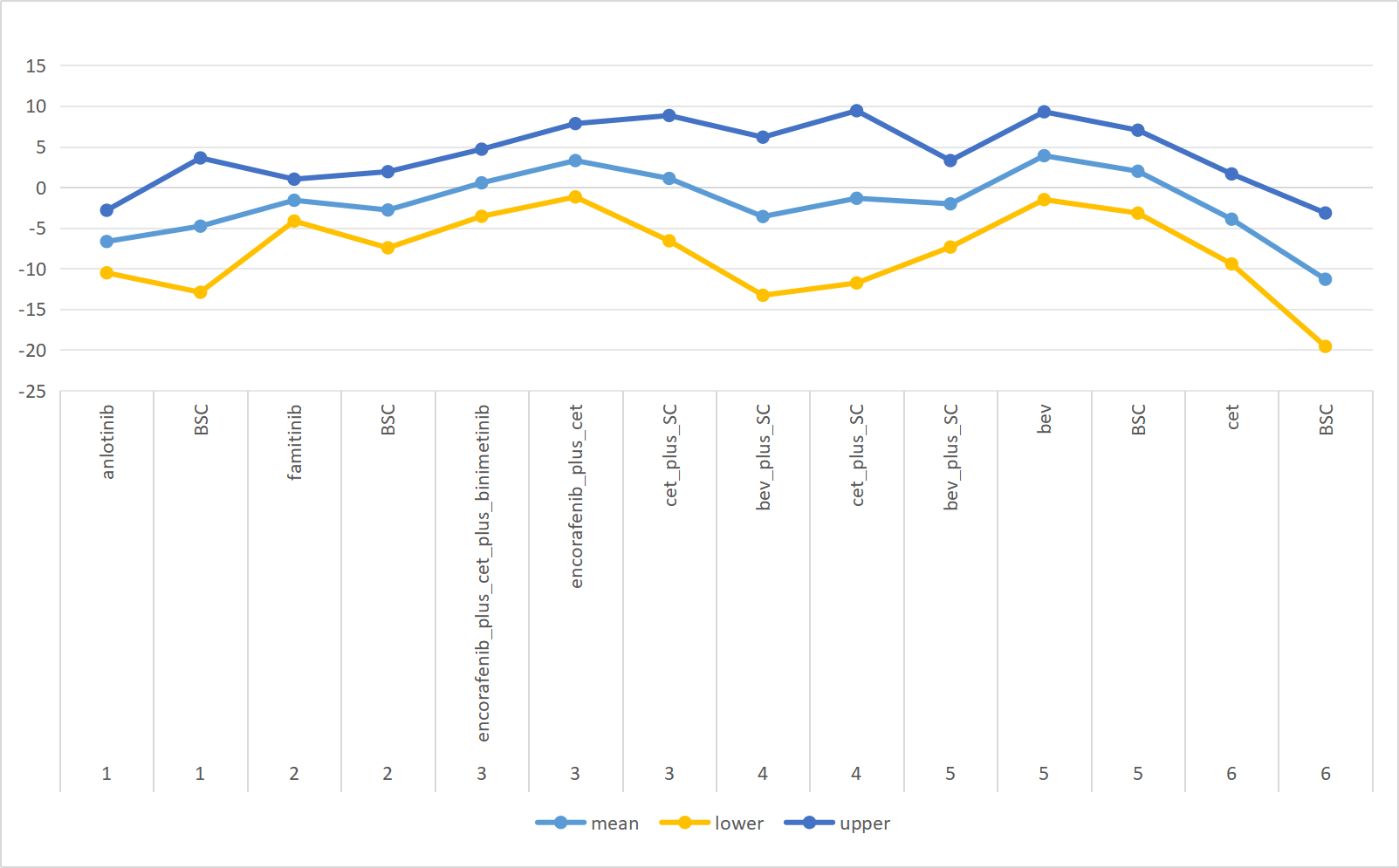


# **Supplementary File 12. Forest Plots Depicting Results of Head-to-head Comparisons**

**Fig.S1 Network: Long-term QLQ-C30 GHS/QOL for Overall Patients**


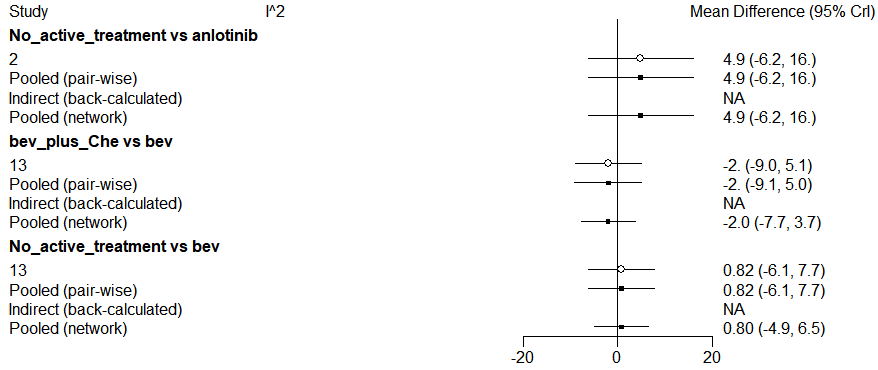


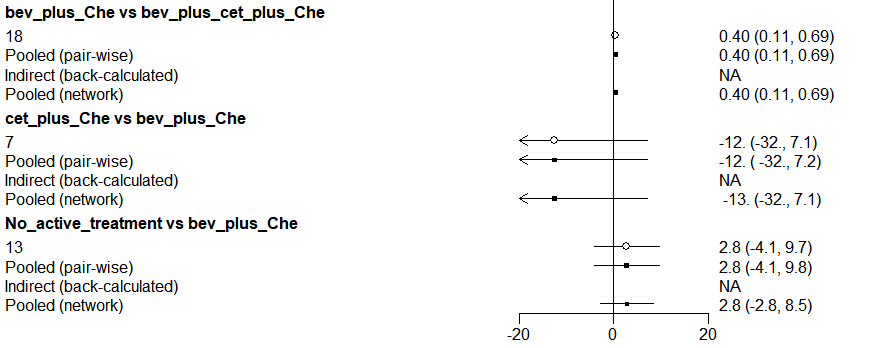


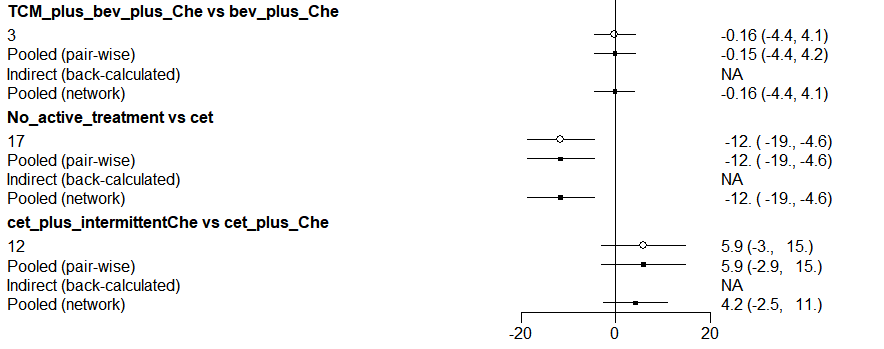


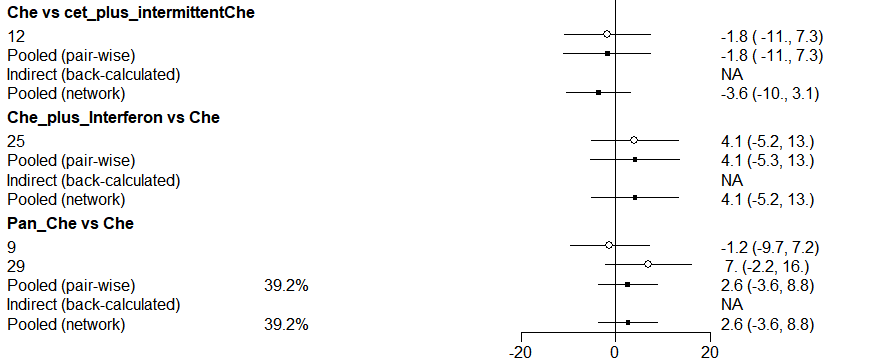


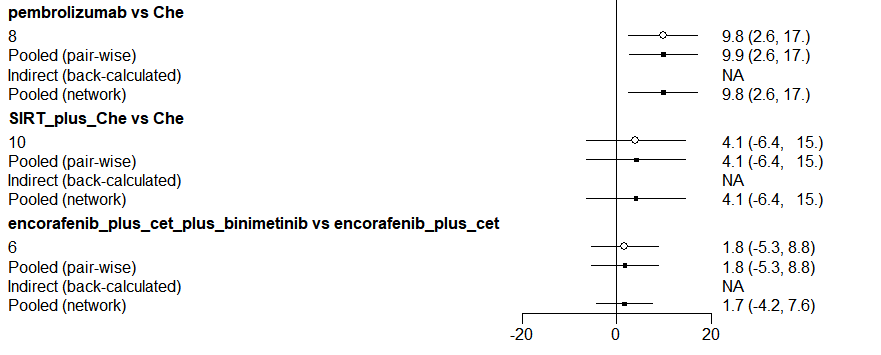


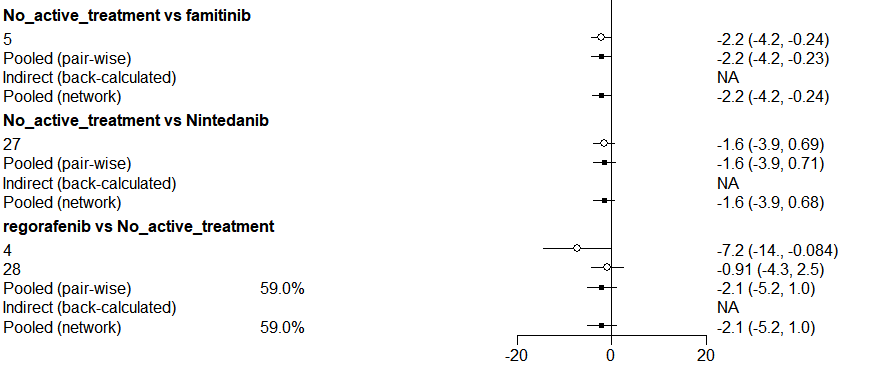


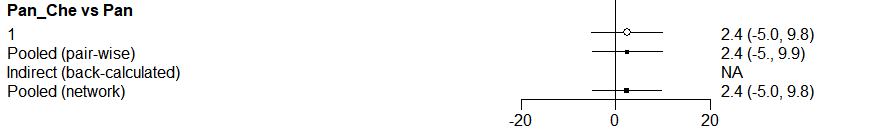


**Fig.S2 Network: Long-term QLQ-C30 GHS/QOL for Patients Received First-line Treatments**


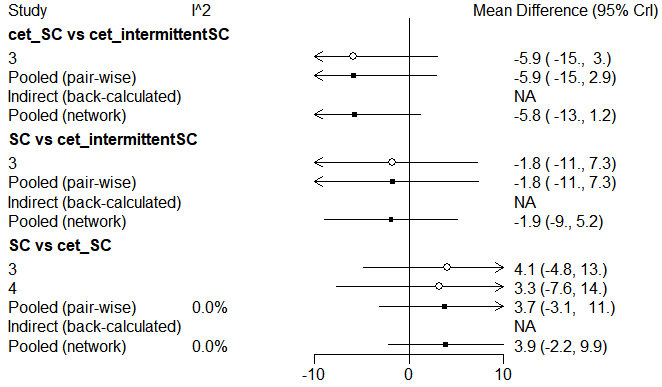


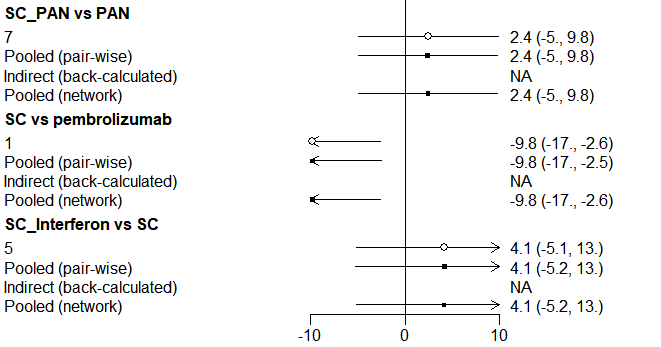


**Fig.S3 Network: Long-term QLQ-C30 GHS/QOL for Patients Received Subsequent-line Treatments**


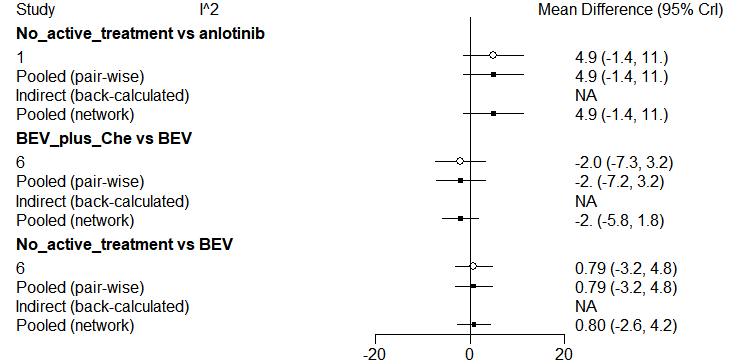


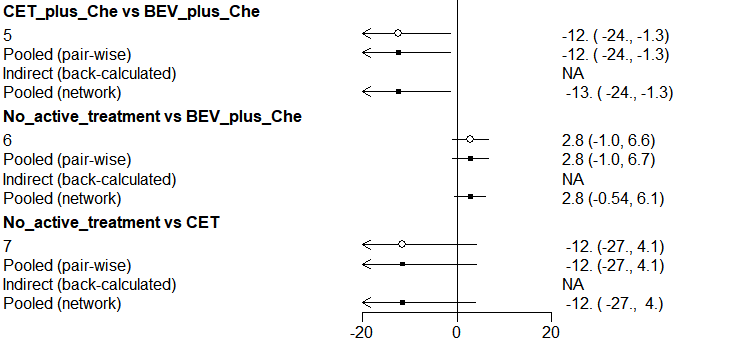


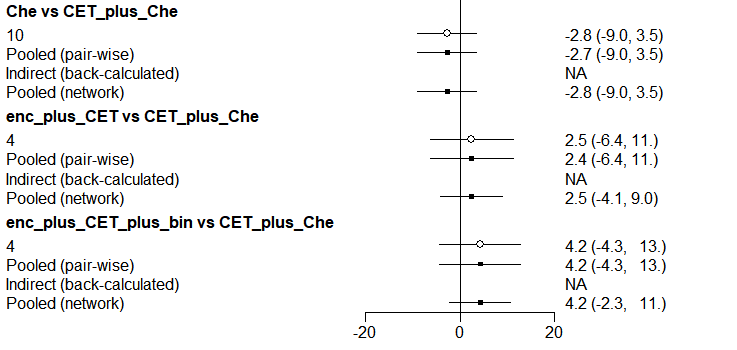


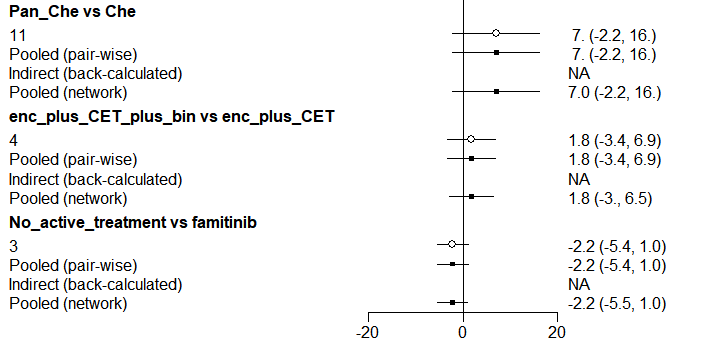


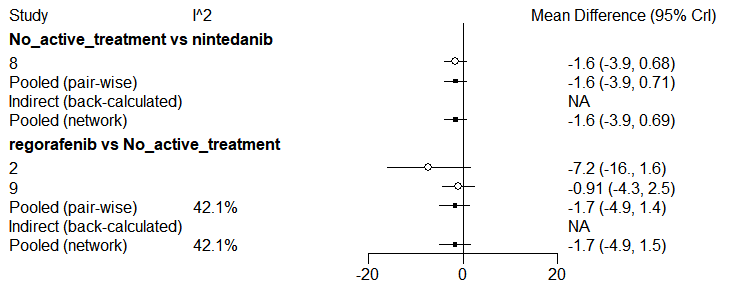


**Fig.S4 Short-term QLQ-C30 GHS/QOL for Overall Patients**


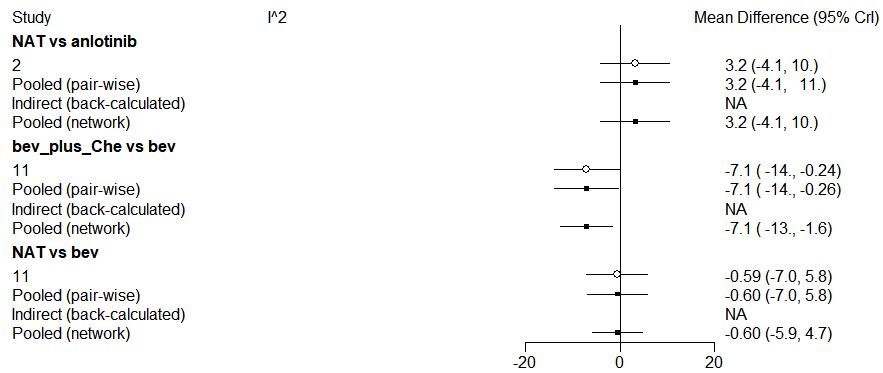


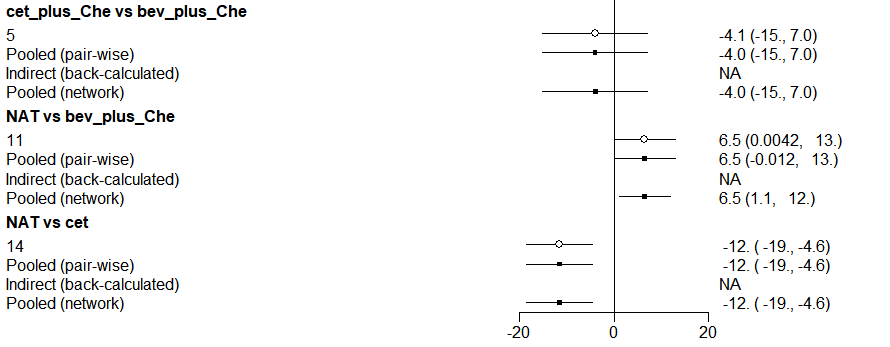

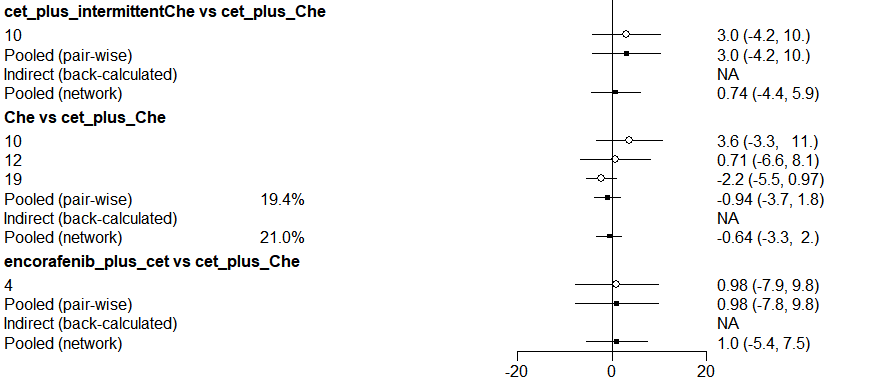


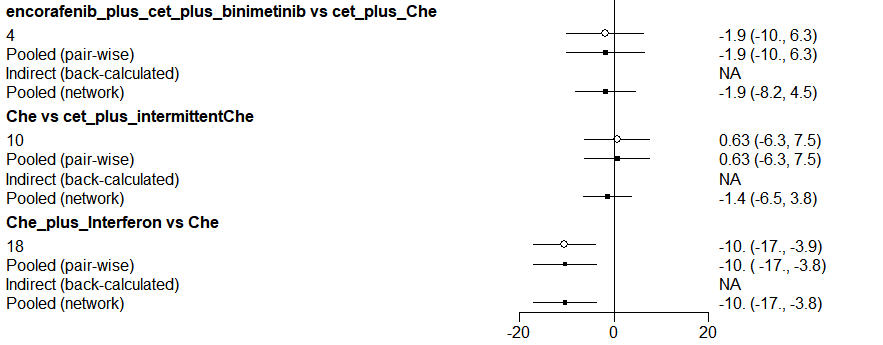


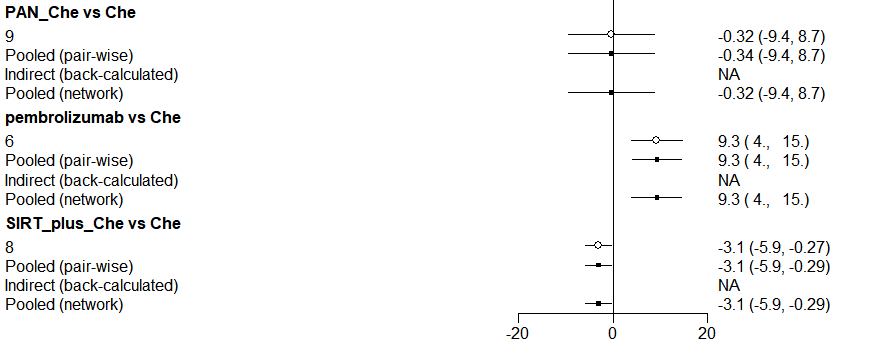


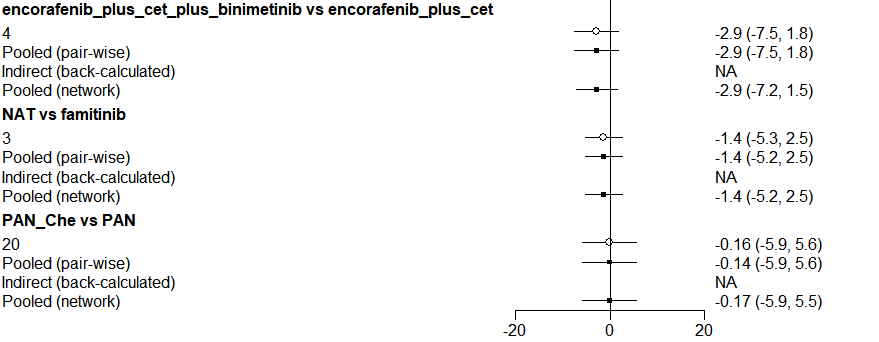


**Fig.S5 Short-term QLQ-C30 GHS/QOL for Patients Received First-line Treatments**


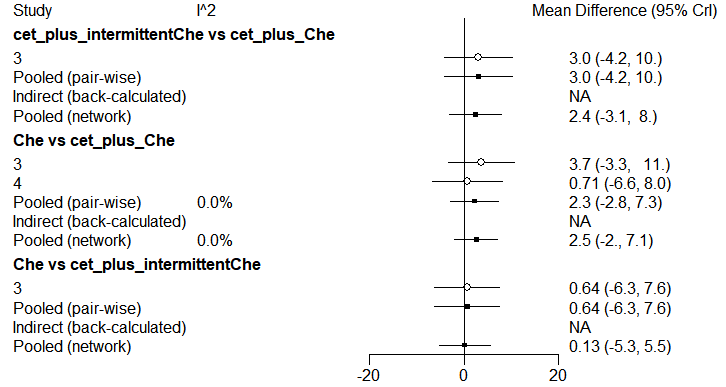


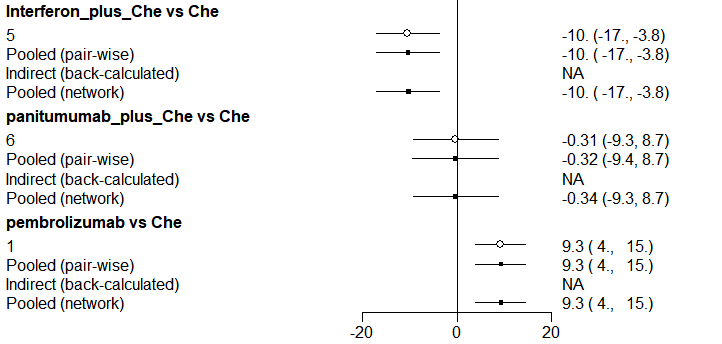


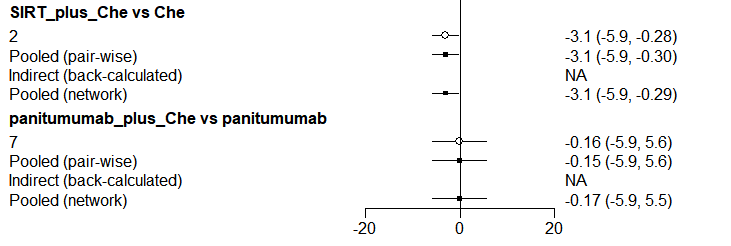


**Fig.S6 EQ-5D**


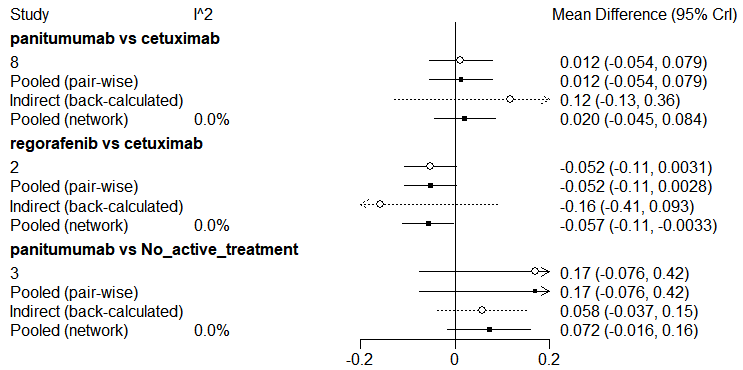


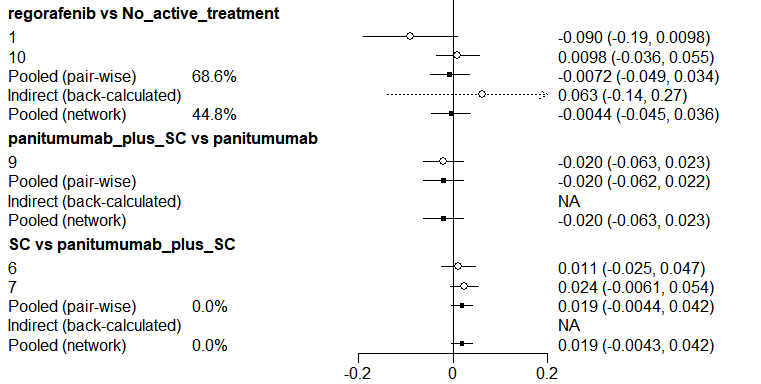


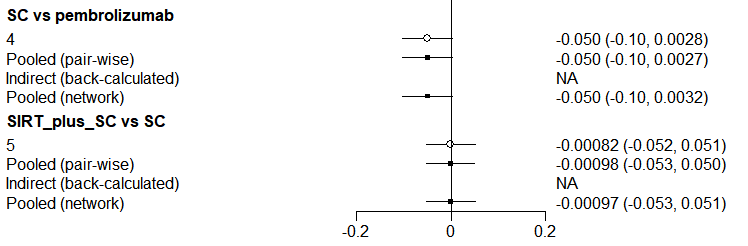


**Fig.S7 Long-term QLQ-C30 GHS/QOL for Overall Patients (Monotherapy VS Combination Therapy VS No Active Treatment)**


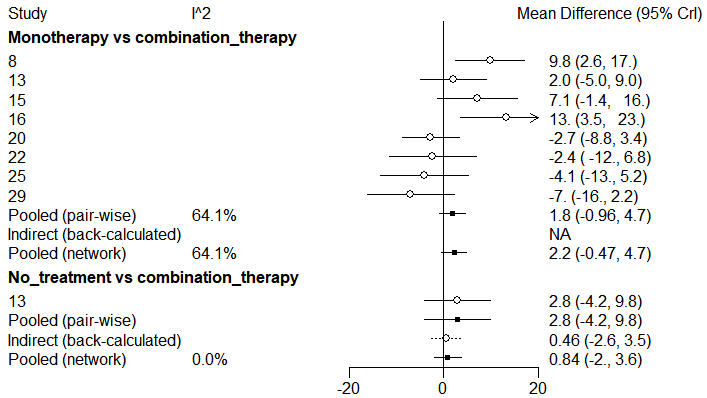


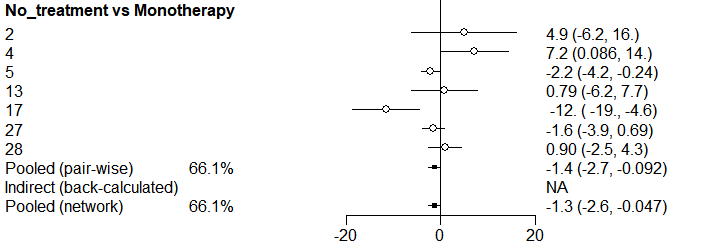


**Fig.S8 Short-term QLQ-C30 GHS/QOL for overall patients (Monotherapy VS Combination therapy VS No active treatment)**


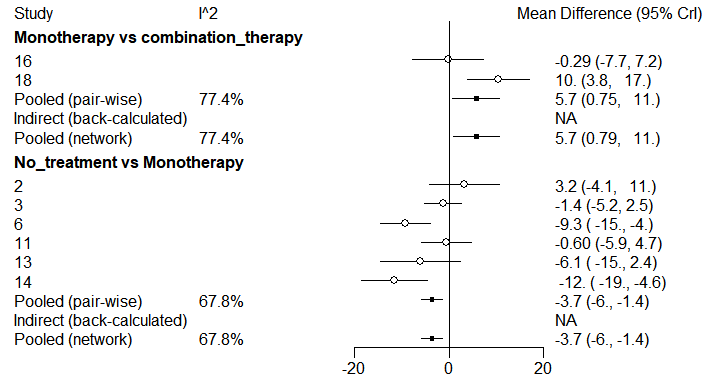


**Fig.S9 Long-term QLQ-C30 GHS/QOL for overall Patients (Therapeutic Modalities Comparison)**


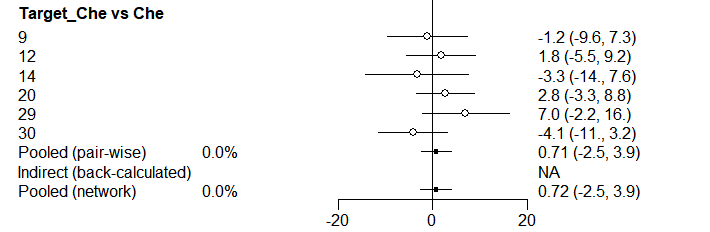


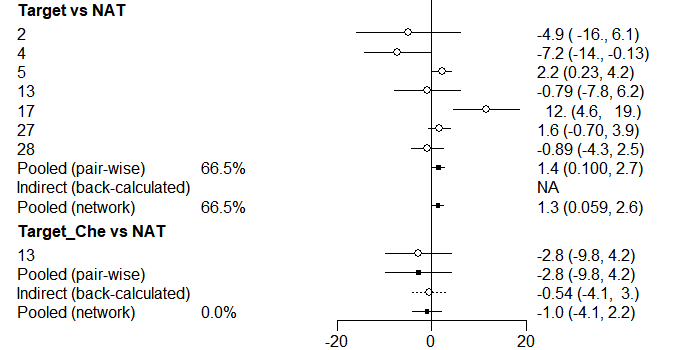


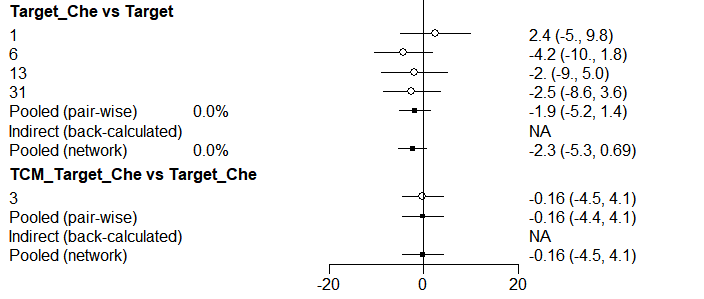


**Fig.S10 Short-term QLQ-C30 GHS/QOL for Overall Patients (Therapeutic Modalities Comparison)**


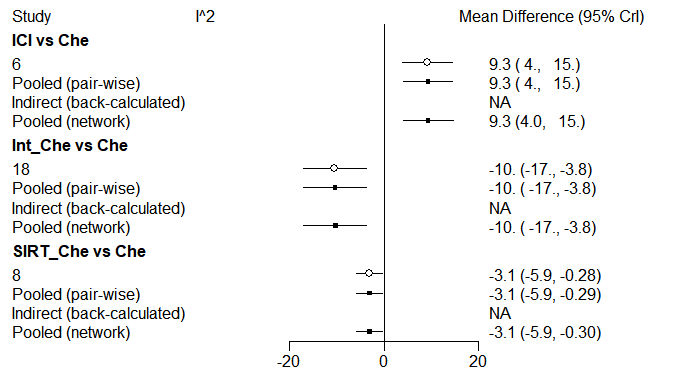


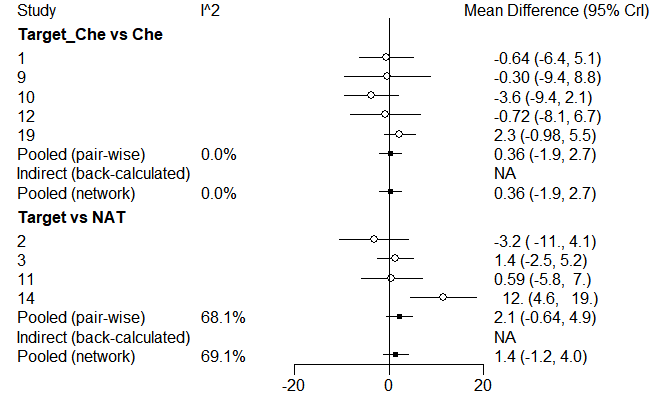


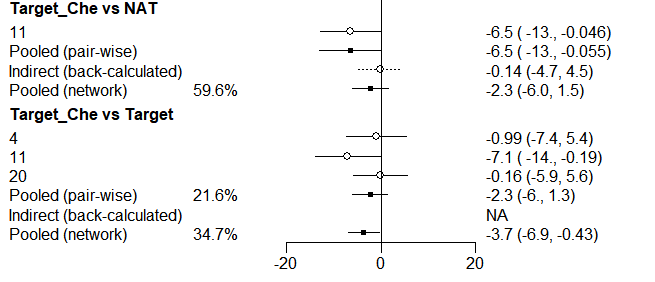


**Supplementary File 13. Node-splitting Analysis of Inconsistency**

**Fig.S1 EQ-5D**


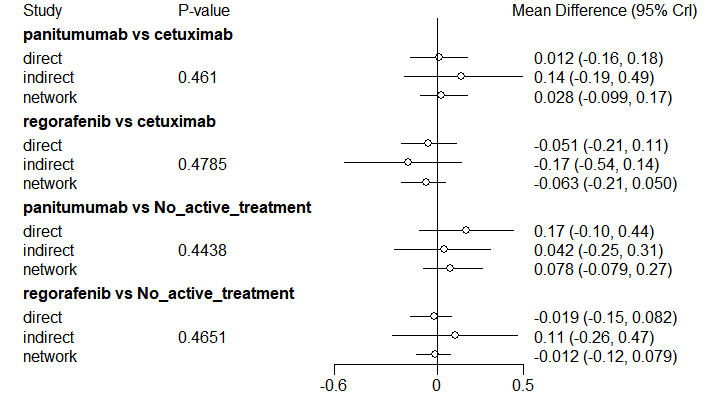


**Fig.S2 Long-term QLQ-C30 GHS/QOL for Overall Patients (Monotherapy VS Combination Therapy VS No Active Treatment)**


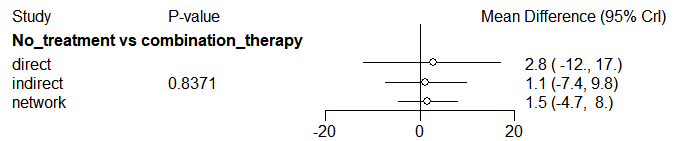
**Fig.S3 Long-term QLQ-C30 GHS/QOL for Overall Patients (Therapeutic Modalities Comparison)**


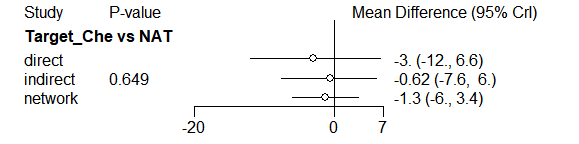


**Fig.S4 Short-term QLQ-C30 GHS/QOL for Overall Patients (Therapeutic Modalities Comparison)**


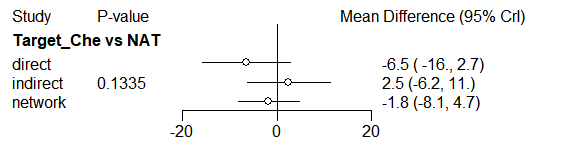


Note: The p-value assesses whether the difference between two sources of evidence is statistically significant. If the p-value is less than 0.05, it suggests a significant inconsistency between direct and indirect evidence, indicating a contradiction.

# **Supplementary File 14. Brooks-Gelman-Rubin Diagnostic**

**Fig.S1 Network: Long-term QLQ-C30 GHS/QOL for Overall Patients**


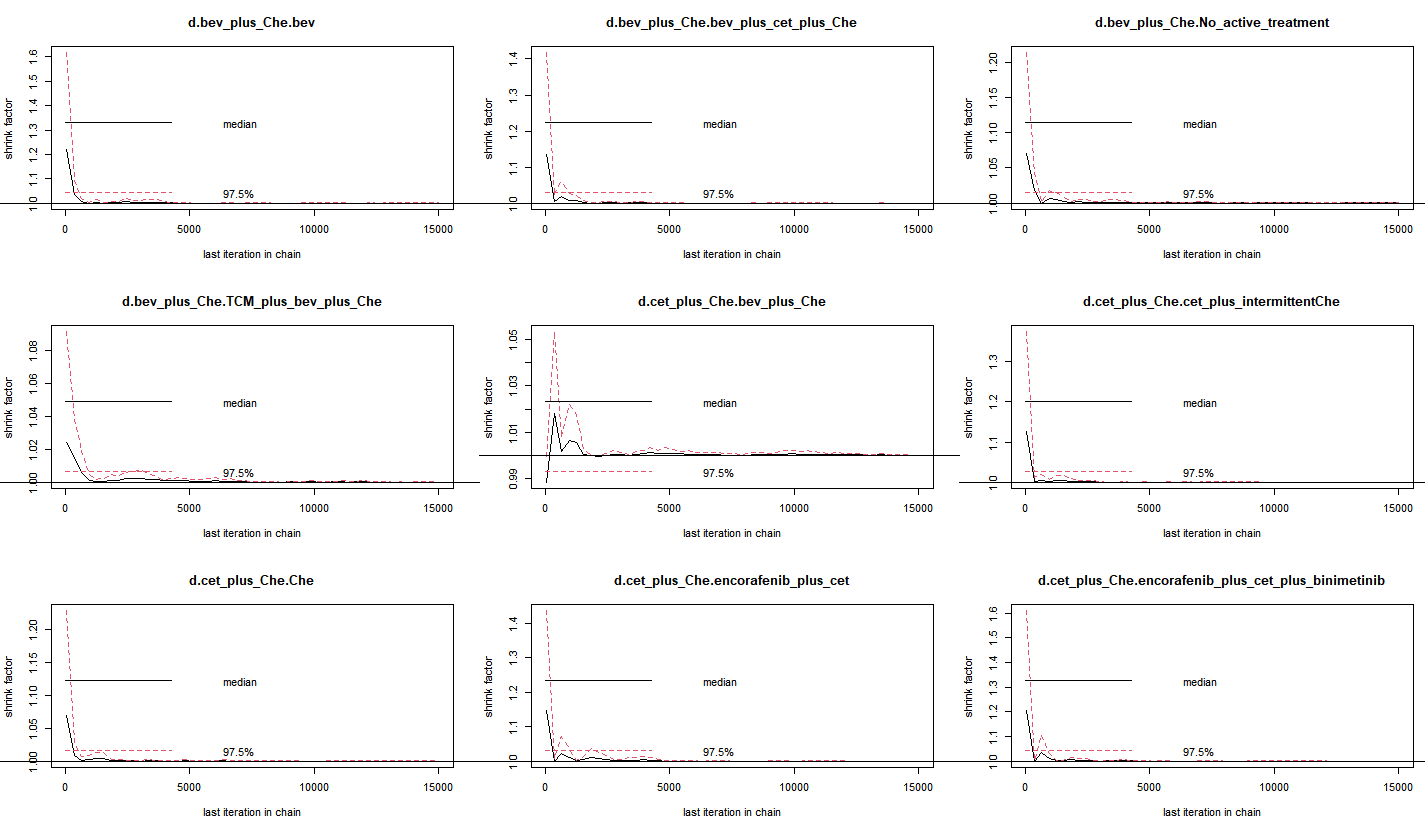


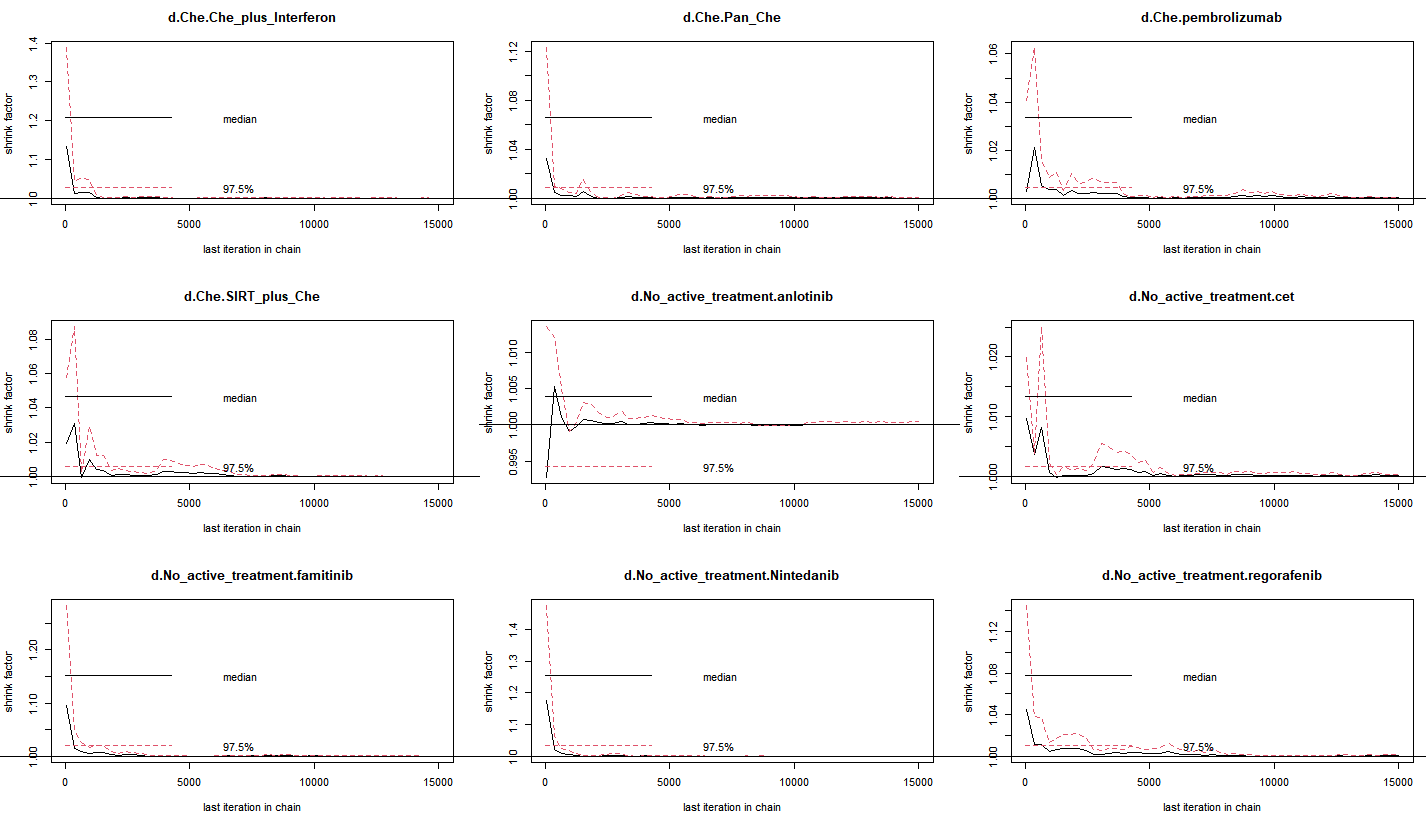


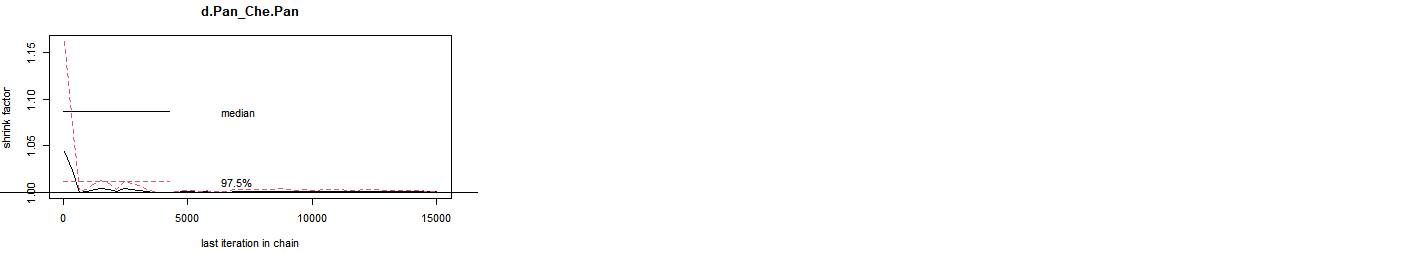


**Fig.S2 Network: Long-term QLQ-C30 GHS/QOL for Patients Received First-line Treatments**


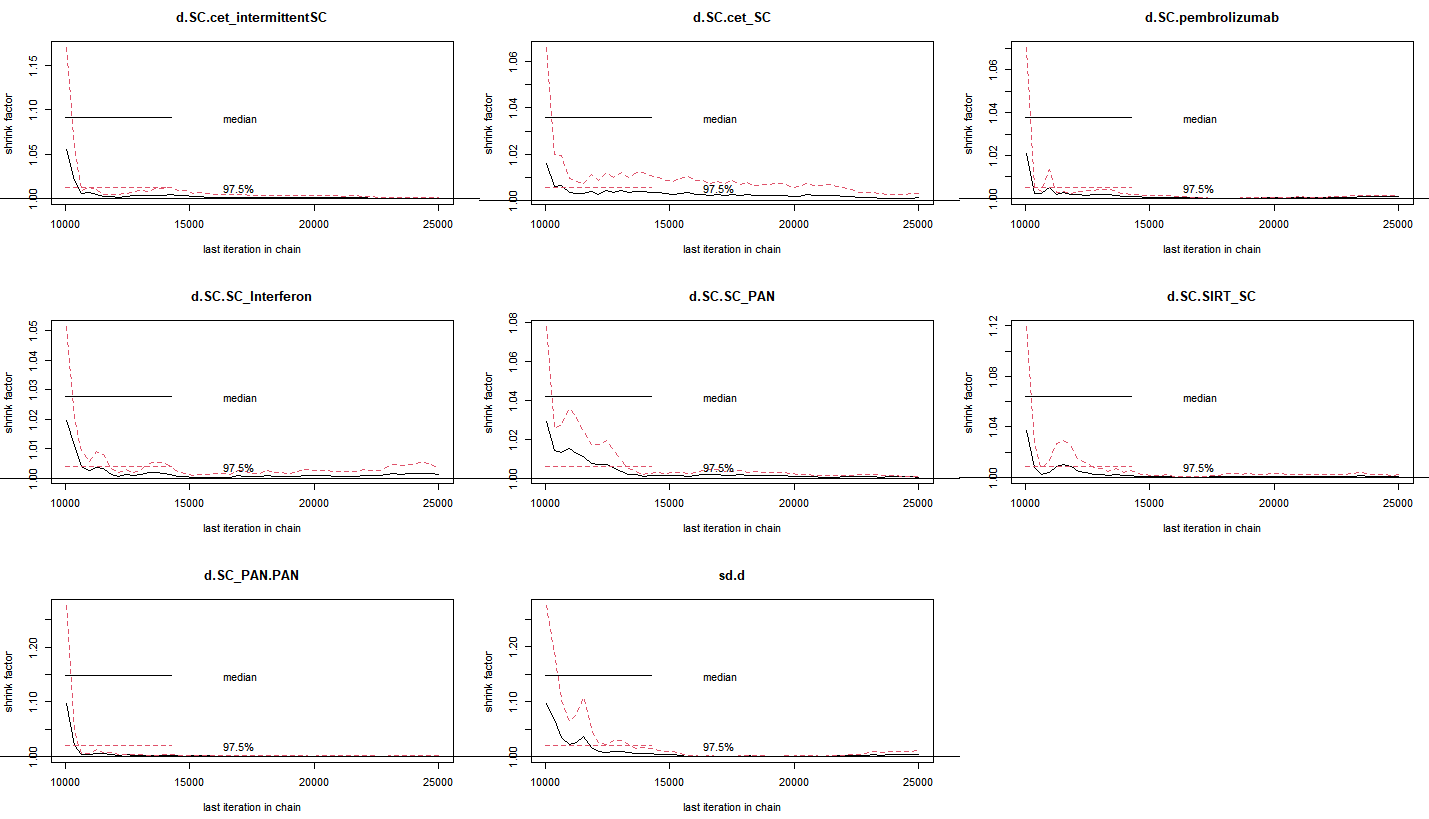


**Fig.S3 Network: Long-term QLQ-C30 GHS/QOL for Patients Received Subsequent-line Treatments**


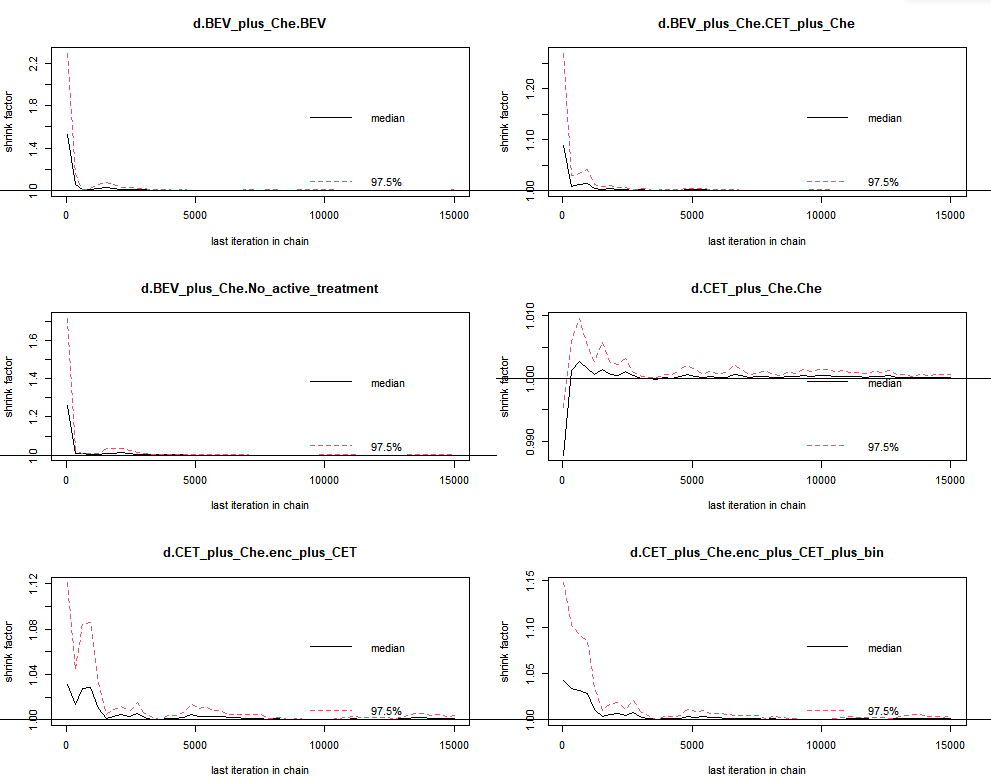


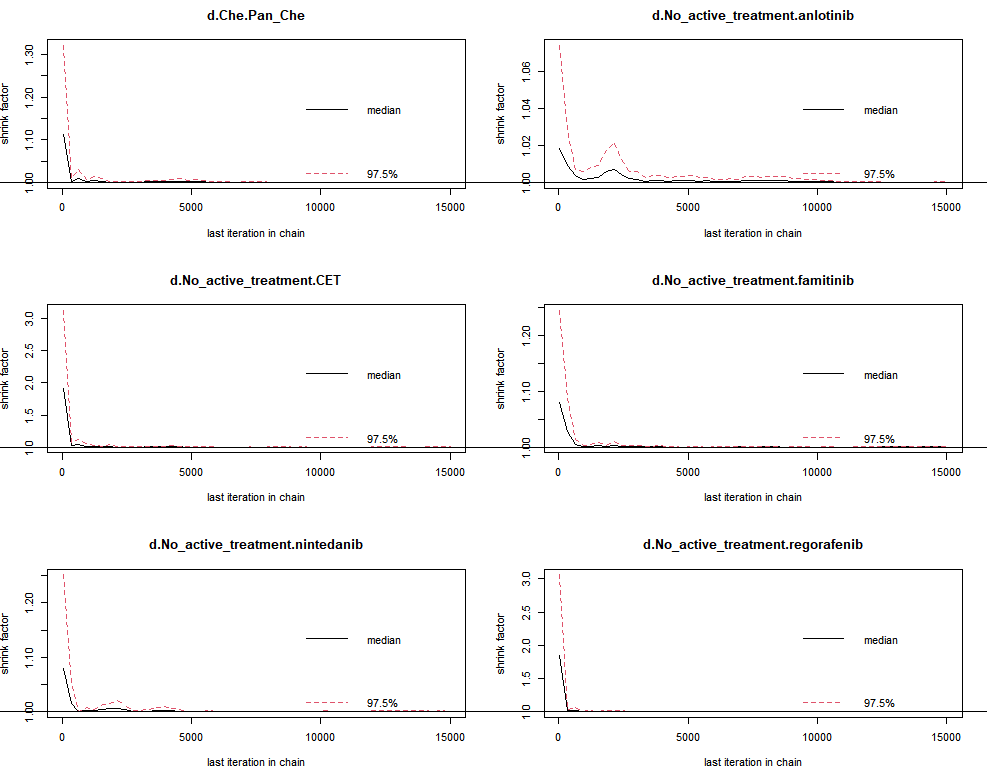


**Fig.S4 Short-term QLQ-C30 GHS/QOL for Overall Patients**


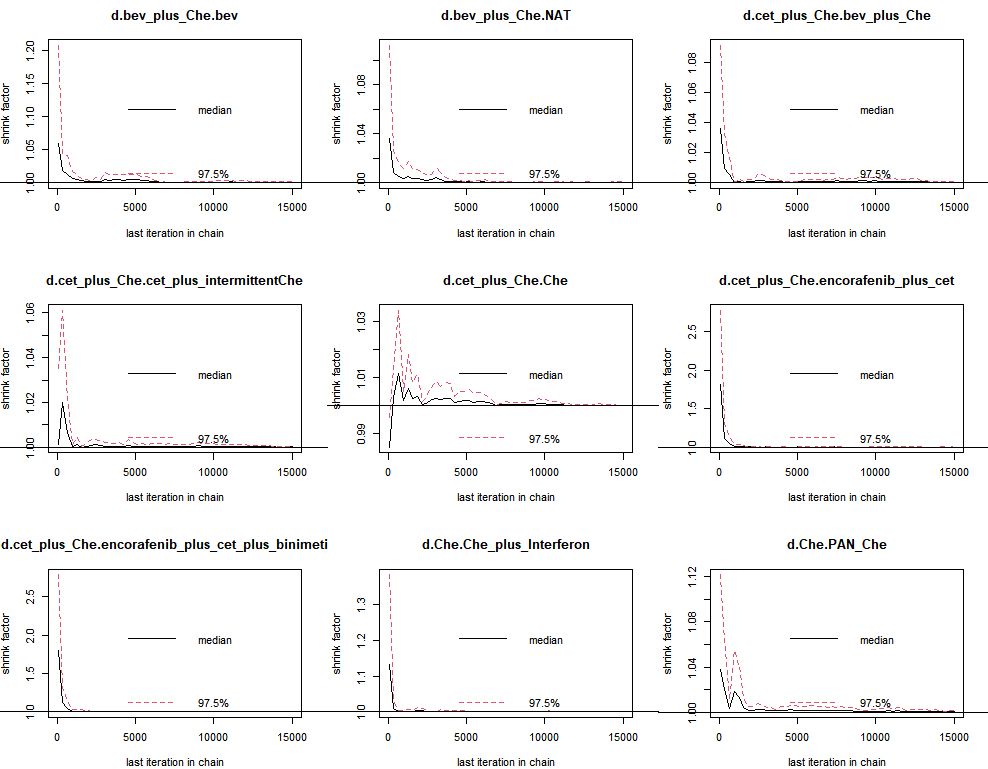


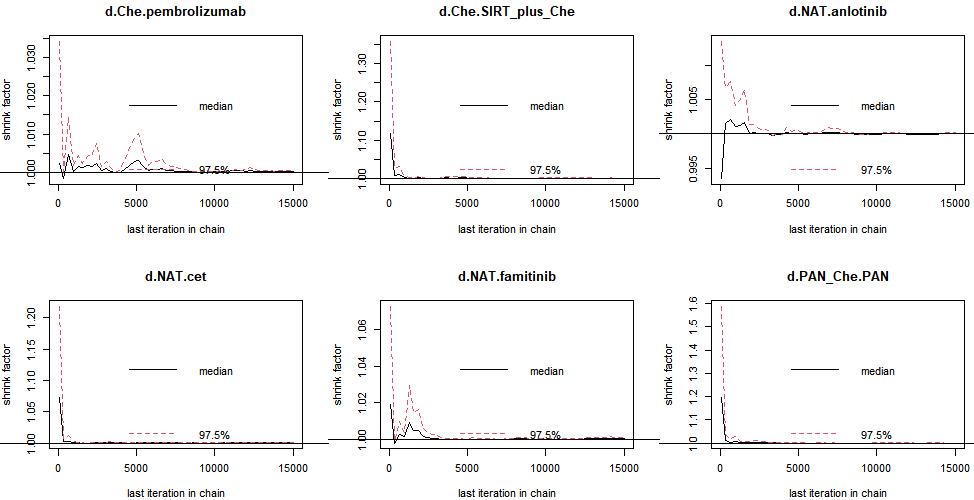


**Fig.S5 Short-term QLQ-C30 GHS/QOL for Patients Received First-line Treatments**


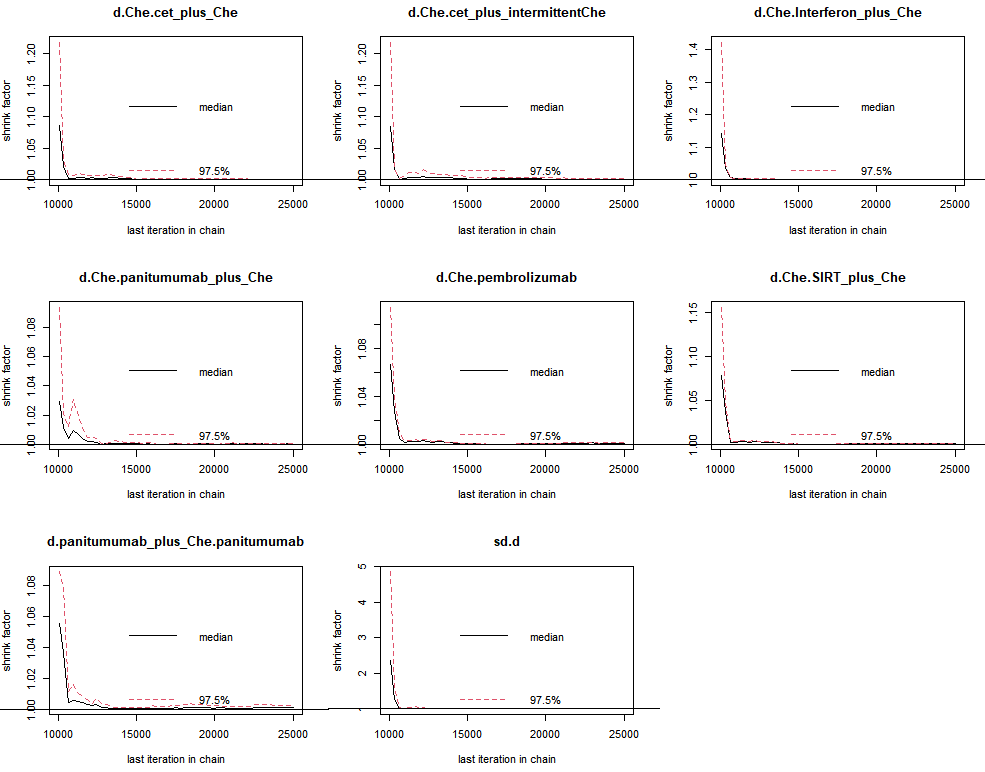


**Fig.S6 Short-term QLQ-C30 GHS/QOL for Patients Received Subsequent-line Treatments**


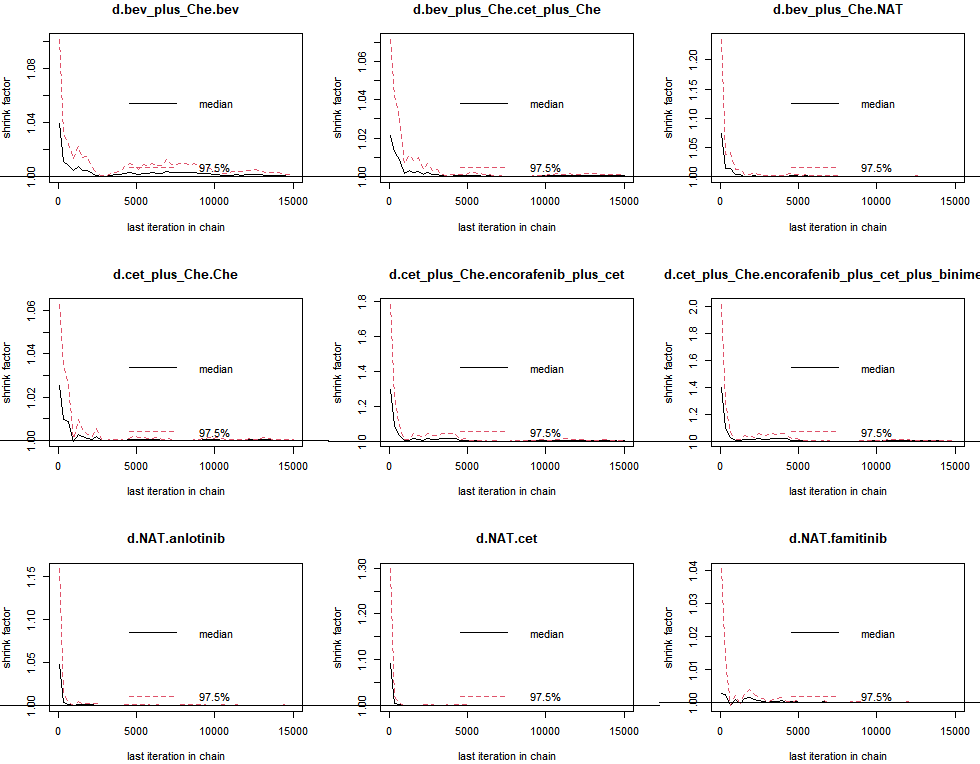


**Fig.S7 EQ-5D**


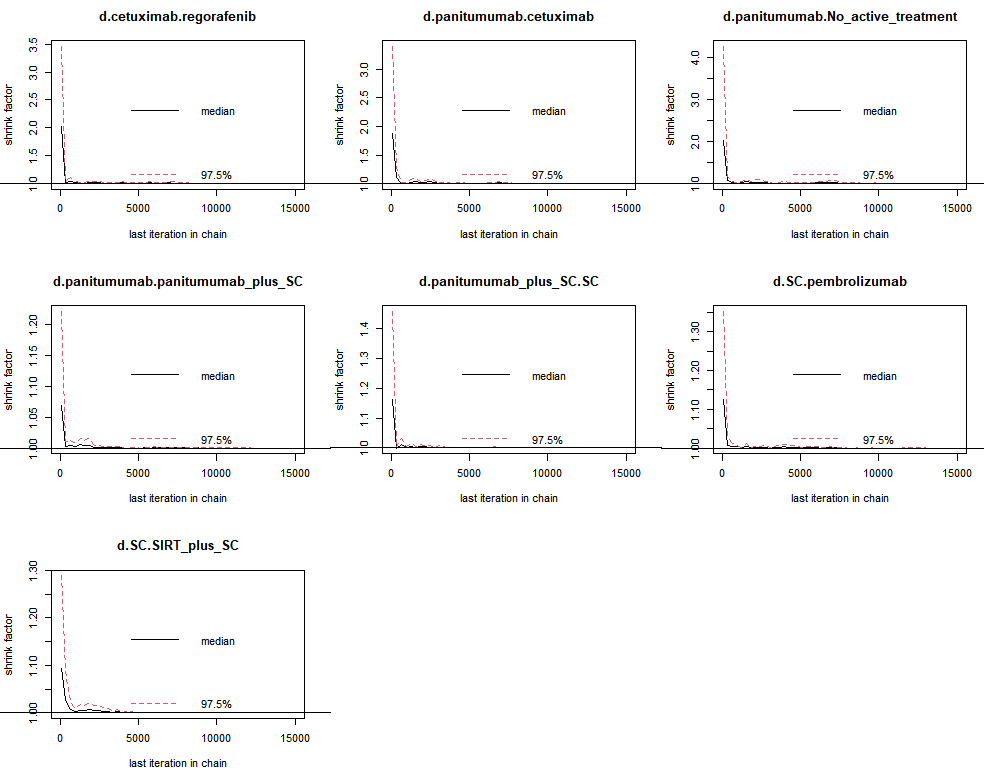


**Fig.S8 Long-term QLQ-C30 GHS/QOL for Overall Patients (Monotherapy VS Combination Therapy VS No Active Treatment)**


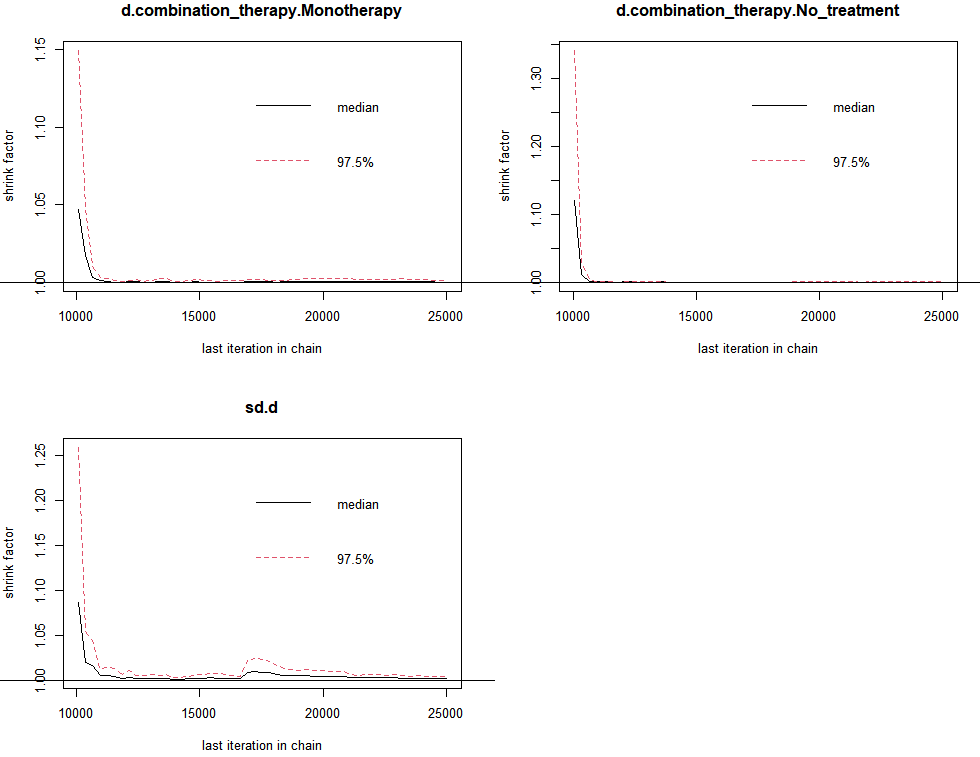


**Fig.S9 Short-term QLQ-C30 GHS/QOL for Overall Patients (Monotherapy VS Combination Therapy VS No Active Treatment)**


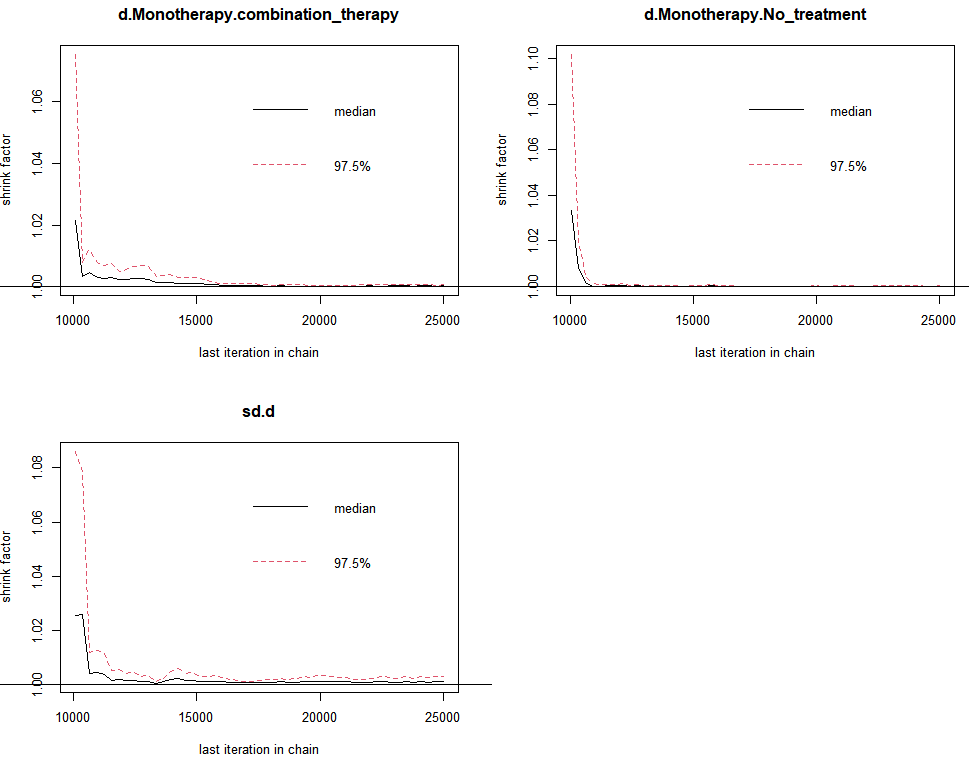


**Fig.S10 Long-term QLQ-C30 GHS/QOL for Overall Patients (Therapeutic Modalities Comparison)**


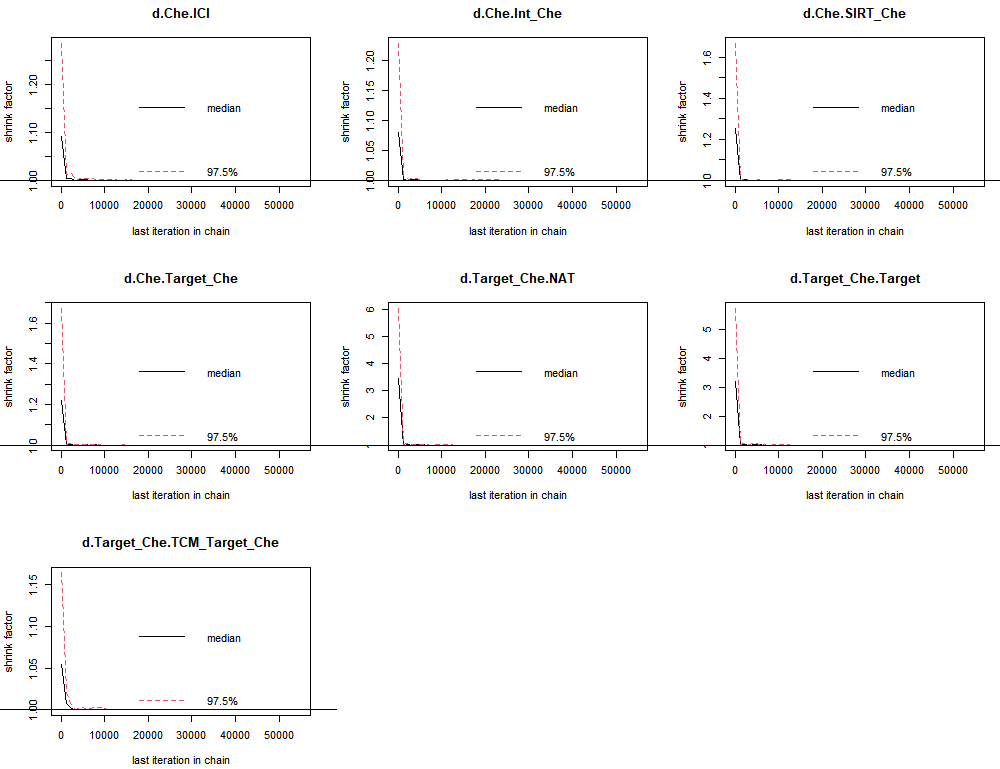


**Fig.S11 Short-term QLQ-C30 GHS/QOL for Overall Patients (Therapeutic Modalities Comparison)**


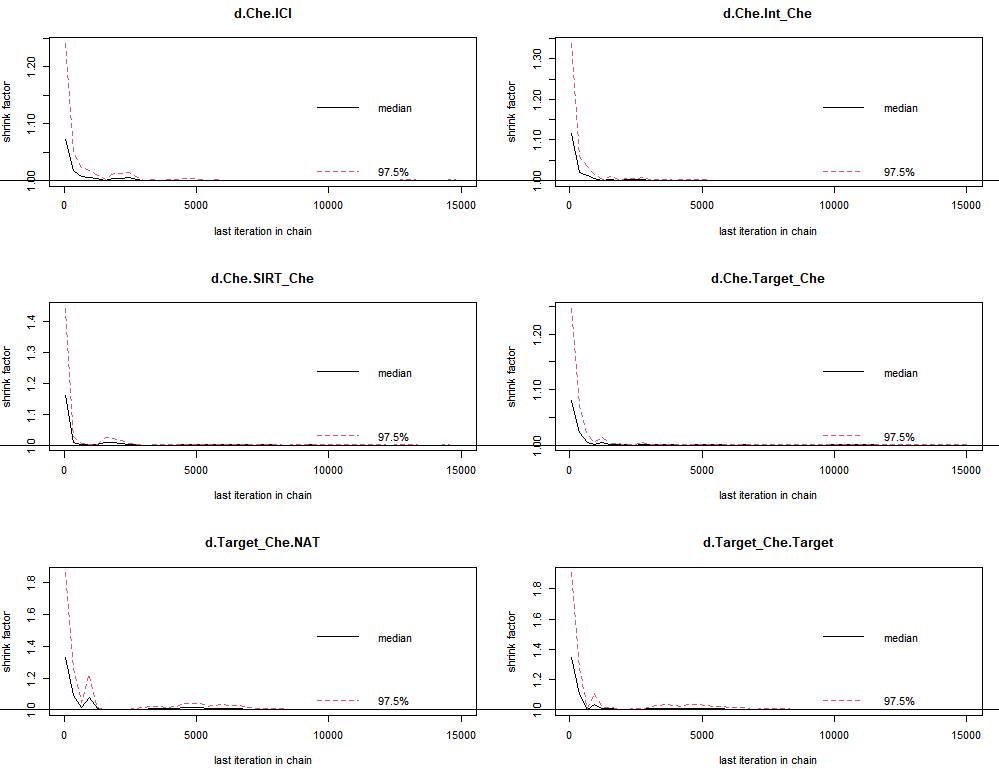


# **Supplementary File 15 Summarized Results of Sensitivity Analysis, Part 1 (QLQ-C30, Differentiating Chemotherapy)**

Abbreviation ANL: anlotinib; BEV: bevacizumab; CAP, capecitabine; CET: cetuximab; inter Che: intermittent chemotherapy; Che: chemotherapy; INT: interferon; ENC: encorafenib; BIN: binimetinib; FAM: famitinib; FU, fluorouracil; ICI: Immunotherapy; IRI: Irinotecan; LV, leucovorin; NAT: No active treatment; NIN: nintedanib; Target: Target therapy; TCM: traditional Chinese medicine; PAN: panitumumab; PEM: pembrolizumab; REG: regorafenib; SIRT: Selective Internal Radiation Therapy

*:The asterisk (*) symbol used in the file denotes statistical significance. Specifically, we have used it to highlight results where the differences were found to be statistically significant. Conventionally, this indicates that the p-value for the associated test was below the predetermined threshold for significance, set at 0.05.

Note: Red coloration signifies that the treatment under consideration shows superiority over the control. Conversely, green indicates inferiority of the treatment relative to the control. White is used to denote that the effects between the treatment and control groups are relatively similar. Additionally, the intensity of the color correlates with the magnitude of the effect difference or the level of statistical significance; the deeper the color, the more pronounced the treatment effect or the higher the statistical significance

QLQ-C30: Regarding the range of the GHS/QoL subscale from the EORTC QLQ-C30 questionnaire, it typically ranges from 0 to 100. A high value on the GHS/QoL scale is considered indicative of good global health status and higher quality of life, whereas a low score would suggest poorer health and quality of life issues. As for the minimal clinically important difference for the GHS/QoL, a difference of 5 to 10 points on the GHS/QoL scale is often considered as a minimal clinically significant change

SUCRA：SUCRA values are interpreted as indicating the relative ranking of treatments. These values, which range from 0 to 100%, reflect the likelihood of each treatment being the most effective option within the network, with higher values suggesting better relative performance

**Fig S1 Forest Plots (A. Long-term; B, Short-term)**

| A | 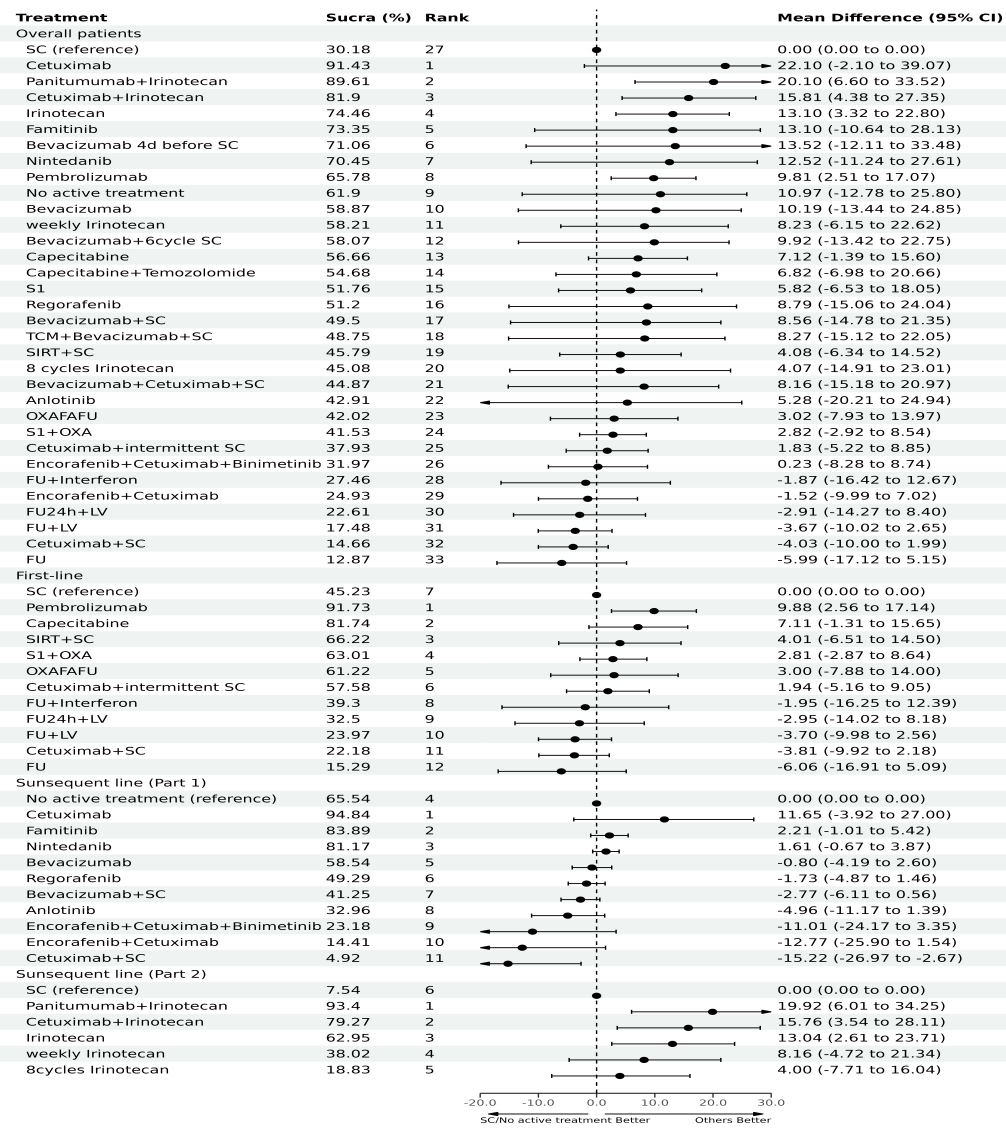 | B | 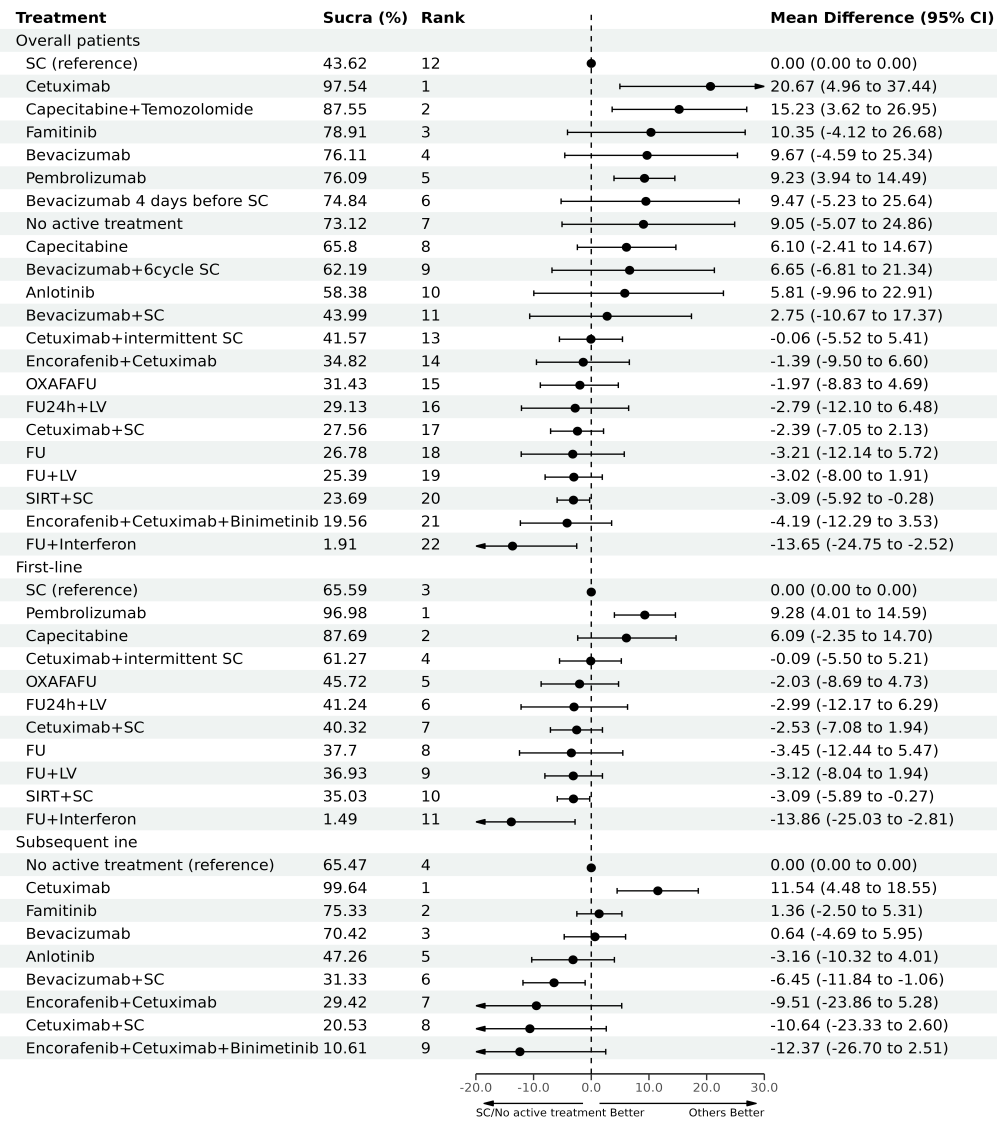 |
| --- | --- | --- | --- |

**Fig.S2 Long-term Sensitivity Analysis for First-line Patients (QLQ-C30, Differentiating Chemotherapy)**


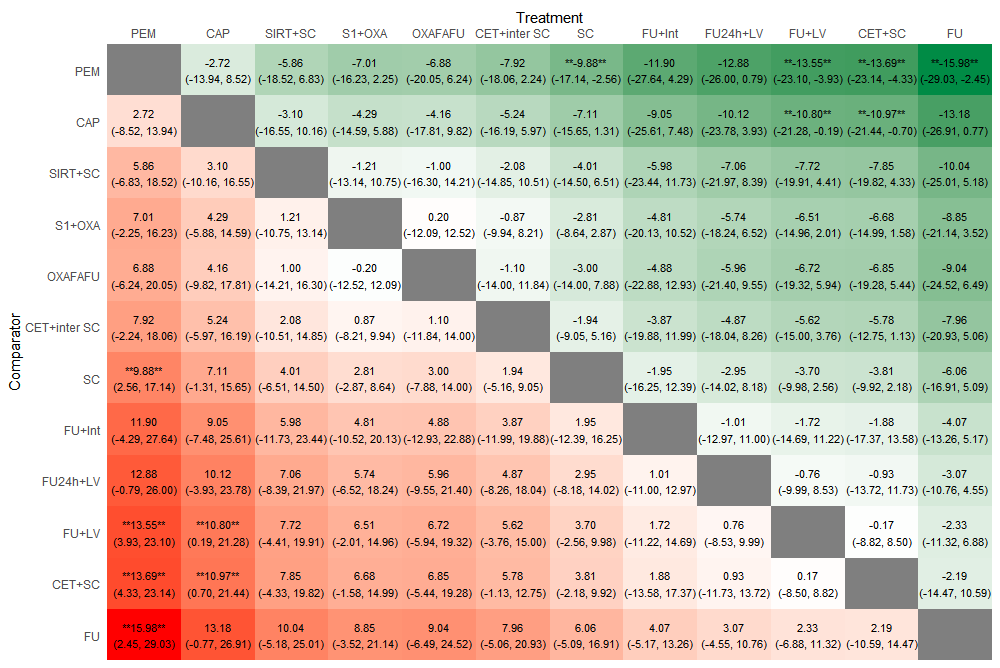


**Fig.S3 Long-term Sensitivity Analysis for Subsequent-line Patients (QLQ-C30, Differentiating Chemotherapy)**


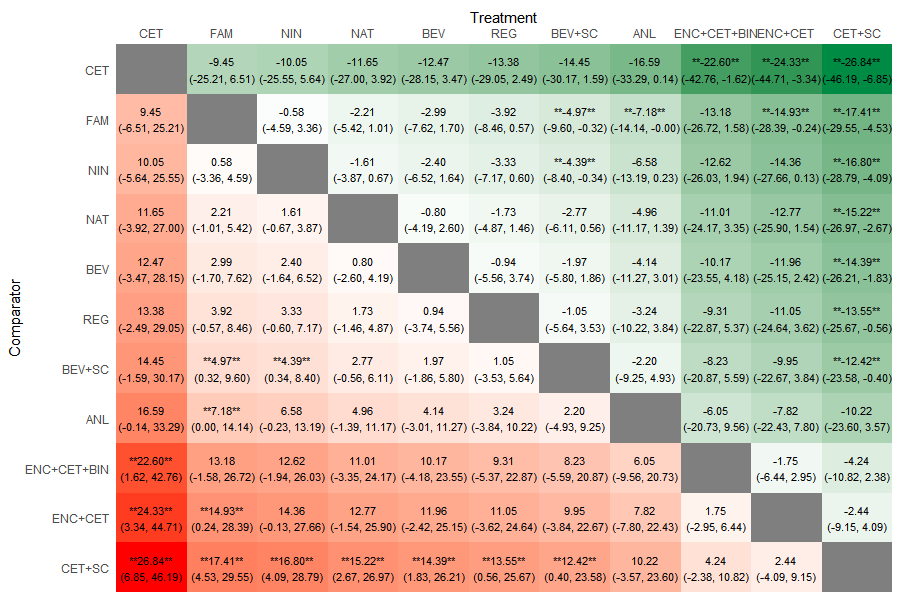


**Fig.S4 Long-term Sensitivity Analysis for All Patients (QLQ-C30, Differentiating Chemotherapy)**

**Fig.S5 Short-term Sensitivity Analysis for First-line Patients (QLQ-C30, Differentiating Chemotherapy)**

**Fig.S6 Short-term Sensitivity Analysis for Subsequent-line Patients (QLQ-C30, Differentiating Chemotherapy)**

**Fig.S7 Short-term Sensitivity Analysis for All Patients (QLQ-C30, Differentiating Chemotherapy)**

**Fig S8 Network Plots (differentiating chemotherapy. A, long-term QLQ-C30 for all patients; B, long-term QLQ-C30 for first-line patients; C, long-term QLQ-C30 for subsequent-line patients (part 1); D, long-term QLQ-C30 for subsequent-line patients (part 2); D, short-term QLQ-C30 for all patients; E, short-term QLQ-C30 for first-line patients; F, long-term QLQ-C30 for subsequent-line patients)**

| **A** |  | **B** |  | **C** |  | **D** |  |
| --- | --- | --- | --- | --- | --- | --- | --- |
| **E** |  | **F** |  | **G** |  |  |  |

Abbreviation ANL: anlotinib; BEV: bevacizumab; BEV+Che: bevacizumab plus chemotherapy; CET: cetuximab; CET+Che: cetuximab plus chemotherapy; CET+inter Che: cetuximab plus intermittent chemotherapy; CET+BEV+Che: cetuximab combined with bevacizumab plus chemotherapy; Che: chemotherapy; Che+INT: interferon plus chemotherapy; ENC+CET: encorafenib plus cetuximab; ENC+CET+BIN: encorafenib combined with cetuximab plus binimetinib; FAM: famitinib; ICI: Immunotherapy; NAT: No active treatment; NIN: nintedanib; Target: Target therapy; Target+Che: Target therapy plus chemotherapy; TCM+BEV+Che: traditional Chinese medicine combined with bevacizumab plus chemotherapy; PAN: panitumumab; PAN+Che: panitumumab plus chemotherapy; PEM: pembrolizumab; REG: regorafenib; SC, standard chemotherapy; SIRT+Che: SIRT plus chemotherapy.

# **Supplementary File 16 Summarized Results of Sensitivity Analysis, Part 2 (QLQ-C30, Random Effects Models)**

Abbreviation ANL: anlotinib; BEV: bevacizumab; CAP, capecitabine; CET: cetuximab; inter Che: intermittent chemotherapy; Che: chemotherapy; INT: interferon; ENC: encorafenib; BIN: binimetinib; FAM: famitinib; FU, fluorouracil; ICI: Immunotherapy; IRI: Irinotecan; LV, leucovorin; NAT: No active treatment; NIN: nintedanib; Target: Target therapy; TCM: traditional Chinese medicine; PAN: panitumumab; PEM: pembrolizumab; REG: regorafenib; SIRT: Selective Internal Radiation Therapy

QLQ-C30: Regarding the range of the GHS/QoL subscale from the EORTC QLQ-C30 questionnaire, it typically ranges from 0 to 100. A high value on the GHS/QoL scale is considered indicative of good global health status and higher quality of life, whereas a low score would suggest poorer health and quality of life issues. As for the minimal clinically important difference for the GHS/QoL, a difference of 5 to 10 points on the GHS/QoL scale is often considered as a minimal clinically significant change

SUCRA：SUCRA values are interpreted as indicating the relative ranking of treatments. These values, which range from 0 to 100%, reflect the likelihood of each treatment being the most effective option within the network, with higher values suggesting better relative performance

**Fig.S1 Comparative Analysis of the Effects of Different Treatment Mechanisms and the Number of Treatment Regimens**

**Fig.S2 Long-term overall analysis (QLQ-C30, without differentiating chemotherapy)**

**Fig.S3 Short-term overall analysis (QLQ-C30, without differentiating chemotherapy)**

# **Supplementary File 17 Summarized Results of Sensitivity Analysis, Part 3 (QLQ-C30, Long-term, Excluding RCTs Targeting on BRAF/RAS Mutated and MSI-H Patients)**

Abbreviation ANL: anlotinib; BEV: bevacizumab; CAP, capecitabine; CET: cetuximab; inter Che: intermittent chemotherapy; Che: chemotherapy; INT: interferon; ENC: encorafenib; BIN: binimetinib; FAM: famitinib; FU, fluorouracil; ICI: Immunotherapy; IRI: Irinotecan; LV, leucovorin; NAT: No active treatment; NIN: nintedanib; Target: Target therapy; TCM: traditional Chinese medicine; PAN: panitumumab; PEM: pembrolizumab; REG: regorafenib; SIRT: Selective Internal Radiation Therapy

*:The asterisk (*) symbol used in the file denotes statistical significance. Specifically, we have used it to highlight results where the differences were found to be statistically significant. Conventionally, this indicates that the p-value for the associated test was below the predetermined threshold for significance, set at 0.05.

Note: Red coloration signifies that the treatment under consideration shows superiority over the control. Conversely, green indicates inferiority of the treatment relative to the control. White is used to denote that the effects between the treatment and control groups are relatively similar. Additionally, the intensity of the color correlates with the magnitude of the effect difference or the level of statistical significance; the deeper the color, the more pronounced the treatment effect or the higher the statistical significance

QLQ-C30: Regarding the range of the GHS/QoL subscale from the EORTC QLQ-C30 questionnaire, it typically ranges from 0 to 100. A high value on the GHS/QoL scale is considered indicative of good global health status and higher quality of life, whereas a low score would suggest poorer health and quality of life issues. As for the minimal clinically important difference for the GHS/QoL, a difference of 5 to 10 points on the GHS/QoL scale is often considered as a minimal clinically significant change

SUCRA：SUCRA values are interpreted as indicating the relative ranking of treatments. These values, which range from 0 to 100%, reflect the likelihood of each treatment being the most effective option within the network, with higher values suggesting better relative performance

**Fig.S1 All patients (QLQ-C30, excluding RCTs targeting on BRAF/RAS mutated and MSI-H patients)**

**Fig.S2 All patients (QLQ-C30, excluding RCTs targeting on BRAF/RAS mutated and MSI-H patients)**

**Fig.S3 First-line patients (QLQ-C30, excluding RCTs targeting on BRAF/RAS mutated and MSI-H patients)**

**Fig.S4 Subsequent-line patients (QLQ-C30, excluding RCTs targeting on BRAF/RAS mutated and MSI-H patients)**

# **Supplementary File 18 Summarized Results of Sensitivity Analysis, Part 4 (QLQ-C30, Long-term, Evidence from Randomized Controlled Trials Focused Solely on Patients with Unrestricted Gene Expression)**

Abbreviation ANL: anlotinib; BEV: bevacizumab; CAP, capecitabine; CET: cetuximab; inter Che: intermittent chemotherapy; Che: chemotherapy; INT: interferon; ENC: encorafenib; BIN: binimetinib; FAM: famitinib; FU, fluorouracil; ICI: Immunotherapy; IRI: Irinotecan; LV, leucovorin; NAT: No active treatment; NIN: nintedanib; Target: Target therapy; TCM: traditional Chinese medicine; PAN: panitumumab; PEM: pembrolizumab; REG: regorafenib; SIRT: Selective Internal Radiation Therapy

QLQ-C30: Regarding the range of the GHS/QoL subscale from the EORTC QLQ-C30 questionnaire, it typically ranges from 0 to 100. A high value on the GHS/QoL scale is considered indicative of good global health status and higher quality of life, whereas a low score would suggest poorer health and quality of life issues. As for the minimal clinically important difference for the GHS/QoL, a difference of 5 to 10 points on the GHS/QoL scale is often considered as a minimal clinically significant change

SUCRA：SUCRA values are interpreted as indicating the relative ranking of treatments. These values, which range from 0 to 100%, reflect the likelihood of each treatment being the most effective option within the network, with higher values suggesting better relative performance

# **Supplementary File 19 Summarized Results of Sensitivity Analysis, Part 5 (QLQ-C30, Long-term, Focused on Wild-type KRAS Patients)**

Abbreviation CET: cetuximab; Che: chemotherapy; PAN: panitumumab

QLQ-C30: Regarding the range of the GHS/QoL subscale from the EORTC QLQ-C30 questionnaire, it typically ranges from 0 to 100. A high value on the GHS/QoL scale is considered indicative of good global health status and higher quality of life, whereas a low score would suggest poorer health and quality of life issues. As for the minimal clinically important difference for the GHS/QoL, a difference of 5 to 10 points on the GHS/QoL scale is often considered as a minimal clinically significant change

SUCRA：SUCRA values are interpreted as indicating the relative ranking of treatments. These values, which range from 0 to 100%, reflect the likelihood of each treatment being the most effective option within the network, with higher values suggesting better relative performance

League Table

| Che (SUCRA 69.28) |  |  |  |
| --- | --- | --- | --- |
| 1.14 (-7.35 to 9.55) | PAN+Che (SUCRA 58.79) |  |  |
| 3.27 (-6.46 to 12.99) | 2.17 (-10.81 to 14.99) | CET+Che (SUCRA 38.06) |  |
| 3.51 (-7.71 to 14.70) | 2.40 (-4.99 to 9.76) | 0.27 (-14.59 to 15.13) | PAN (SUCRA 33.87) |

**References**

[1]. Avallone, A., et al., Effect of Bevacizumab in Combination With Standard Oxaliplatin-Based Regimens in Patients With Metastatic Colorectal Cancer: A Randomized Clinical Trial. JAMA Netw Open, 2021. 4(7): p. e2118475.

[2]. Chi, Y., et al., Anlotinib Monotherapy for Refractory Metastatic Colorectal Cancer: A Double-Blinded, Placebo-Controlled, Randomized Phase III Trial (ALTER0703). Oncologist, 2021. 26(10): p. e1693-e1703.

[3]. Liu, N., et al., Traditional Chinese Medicine Combined With Chemotherapy and Cetuximab or Bevacizumab for Metastatic Colorectal Cancer: A Randomized, Double-Blind, Placebo-Controlled Clinical Trial. Front Pharmacol, 2020. 11: p. 478.

[4]. Xu, J., et al., Regorafenib in Chinese patients with metastatic colorectal cancer: Subgroup analysis of the phase 3 CONCUR trial. J Gastroenterol Hepatol, 2020. 35(8): p. 1307-1316.

[5]. Li, J., et al., Regorafenib plus best supportive care versus placebo plus best supportive care in Asian patients with previously treated metastatic colorectal cancer (CONCUR): a randomised, double-blind, placebo-controlled, phase 3 trial. Lancet Oncol, 2015. 16(6): p. 619-29.

[6]. Xu, R.H., et al., Famitinib versus placebo in the treatment of refractory metastatic colorectal cancer: a multicenter, randomized, double-blinded, placebo-controlled, phase II clinical trial. Chin J Cancer, 2017. 36(1): p. 97.

[7]. Kopetz, S., et al., Quality of life with encorafenib plus cetuximab with or without binimetinib treatment in patients with BRAF V600E-mutant metastatic colorectal cancer: patient-reported outcomes from BEACON CRC. ESMO Open, 2022. 7(3): p. 100477.

[8]. Bertaut, A., et al., Health-Related Quality of Life Analysis in Metastatic Colorectal Cancer Patients Treated by subsequent-line Chemotherapy, Associated With Either Cetuximab or Bevacizumab: The PRODIGE 18 Randomized Phase II Study. Clin Colorectal Cancer, 2022. 21(2): p. e49-e61.

[9]. Liposits, G., et al., Quality of Life in Vulnerable Older Patients with Metastatic Colorectal Cancer Receiving Palliative Chemotherapy-The Randomized NORDIC9-Study. Cancers (Basel), 2021. 13(11).

[10]. Andre, T., et al., Health-related quality of life in patients with microsatellite instability-high or mismatch repair deficient metastatic colorectal cancer treated with first-line pembrolizumab versus chemotherapy (KEYNOTE-177): an open-label, randomised, phase 3 trial. Lancet Oncol, 2021. 22(5): p. 665-677.

[11]. Raimondi, A., et al., Health-related quality of life in patients with RAS wild-type metastatic colorectal cancer treated with panitumumab-based first-line treatment strategy: A pre-specified secondary analysis of the Valentino study. Eur J Cancer, 2020. 135: p. 230-239.

[12]. Wolstenholme, J., et al., Quality of life in the FOXFIRE, SIRFLOX and FOXFIRE-global randomised trials of selective internal radiotherapy for metastatic colorectal cancer. Int J Cancer, 2020. 147(4): p. 1078-1085.

[13]. Pietrantonio, F., et al., Capecitabine and Temozolomide versus FOLFIRI in RAS-Mutated, MGMT-Methylated Metastatic Colorectal Cancer. Clin Cancer Res, 2020. 26(5): p. 1017-1024.

[14]. Shitara, K., et al., REVERCE: a randomized phase II study of regorafenib followed by cetuximab versus the reverse sequence for previously treated metastatic colorectal cancer patients. Ann Oncol, 2019. 30(2): p. 259-265.

[15]. Thomsen, M., et al., Health-related quality of life in patients with metastatic colorectal cancer, association with systemic inflammatory response and RAS and BRAF mutation status. Eur J Cancer, 2017. 81: p. 26-35.

[16]. Yamaguchi, K., et al., Quality of Life Analysis in Patients With RAS Wild-Type Metastatic Colorectal Cancer Treated With First-Line Cetuximab Plus Chemotherapy. Clin Colorectal Cancer, 2017. 16(2): p. e29-e37.

[17]. Quidde, J., et al., Quality of life assessment in patients with metastatic colorectal cancer receiving maintenance therapy after first-line induction treatment: a preplanned analysis of the phase III AIO KRK 0207 trial. Ann Oncol, 2016. 27(12): p. 2203-2210.

[18]. Láng, I., et al., Quality of life analysis in patients with KRAS wild-type metastatic colorectal cancer treated first-line with cetuximab plus irinotecan, fluorouracil and leucovorin. Eur J Cancer, 2013. 49(2): p. 439-48.

[19]. Hong, Y.S., et al., Randomized phase II study of capecitabine with or without oxaliplatin as first-line treatment for elderly or fragile patients with metastatic colorectal cancer: a prospective, multicenter trial of the Korean Cancer Study Group CO06-01. Am J Clin Oncol, 2013. 36(6): p. 565-71.

[20]. Bennett, L., et al., Health-related quality of life in patients with metastatic colorectal cancer treated with panitumumab in first- or subsequent-line treatment. Br J Cancer, 2011. 105(10): p. 1495-502.

[21]. Clarke, S.J., et al., Single-agent irinotecan or FOLFIRI as subsequent-line chemotherapy for advanced colorectal cancer; results of a randomised phase II study (DaVINCI) and meta-analysis [corrected]. Eur J Cancer, 2011. 47(12): p. 1826-36.

[22]. Odom, D., et al., Health-related quality of life and colorectal cancer-specific symptoms in patients with chemotherapy-refractory metastatic disease treated with panitumumab. Int J Colorectal Dis, 2011. 26(2): p. 173-81.

[23]. Au, H.J., et al., Health-related quality of life in patients with advanced colorectal cancer treated with cetuximab: overall and KRAS-specific results of the NCIC CTG and AGITG CO.17 Trial. J Clin Oncol, 2009. 27(11): p. 1822-8.

[24]. Tol, J., et al., Chemotherapy, bevacizumab, and cetuximab in metastatic colorectal cancer. N Engl J Med, 2009. 360(6): p. 563-72.

[25]. Comella, P., et al., Randomised trial comparing biweekly oxaliplatin plus oral capecitabine versus oxaliplatin plus i.v. bolus fluorouracil/leucovorin in metastatic colorectal cancer patients: results of the Southern Italy Cooperative Oncology study 0401. J Cancer Res Clin Oncol, 2009. 135(2): p. 217-26.

[26]. Sobrero, A.F., et al., EPIC: phase III trial of cetuximab plus irinotecan after fluoropyrimidine and oxaliplatin failure in patients with metastatic colorectal cancer. J Clin Oncol, 2008. 26(14): p. 2311-9.

[27]. Lal, R., et al., A randomized trial comparing defined-duration with continuous irinotecan until disease progression in fluoropyrimidine and thymidylate synthase inhibitor-resistant advanced colorectal cancer. J Clin Oncol, 2004. 22(15): p. 3023-31.

[28]. Köhne, C.H., et al., Randomized phase III study of high-dose fluorouracil given as a weekly 24-hour infusion with or without leucovorin versus bolus fluorouracil plus leucovorin in advanced colorectal cancer: European organization of Research and Treatment of Cancer Gastrointestinal Group Study 40952. J Clin Oncol, 2003. 21(20): p. 3721-8.

[29]. Fuchs, C.S., et al., Phase III comparison of two irinotecan dosing regimens in subsequent-line therapy of metastatic colorectal cancer. J Clin Oncol, 2003. 21(5): p. 807-14.

[30]. Saltz, L.B., et al., Irinotecan plus fluorouracil and leucovorin for metastatic colorectal cancer. Irinotecan Study Group. N Engl J Med, 2000. 343(13): p. 905-14.

[31]. Hill, M., et al., Impact of protracted venous infusion fluorouracil with or without interferon alfa-2b on tumor response, survival, and quality of life in advanced colorectal cancer. J Clin Oncol, 1995. 13(9): p. 2317-23.

[32]. Price, T.J., et al., Panitumumab versus cetuximab in patients with chemotherapy-refractory wild-type KRAS exon 2 metastatic colorectal cancer (ASPECCT): a randomised, multicentre, open-label, non-inferiority phase 3 study. Lancet Oncol, 2014. 15(6): p. 569-79.

[33]. Peeters, M., et al., Final results from a randomized phase 3 study of FOLFIRI {+/-} panitumumab for subsequent-line treatment of metastatic colorectal cancer. Ann Oncol, 2014. 25(1): p. 107-16.

[34]. Seymour, M.T., et al., Panitumumab and irinotecan versus irinotecan alone for patients with KRAS wild-type, fluorouracil-resistant advanced colorectal cancer (PICCOLO): a prospectively stratified randomised trial. Lancet Oncol, 2013. 14(8): p. 749-59.

[35]. Van Cutsem, E., et al., Nintedanib for the treatment of patients with refractory metastatic colorectal cancer (LUME-Colon 1): a phase III, international, randomized, placebo-controlled study. Ann Oncol, 2018. 29(9): p. 1955-1963.

[36]. Grothey, A., et al., Regorafenib monotherapy for previously treated metastatic colorectal cancer (CORRECT): an international, multicentre, randomised, placebo-controlled, phase 3 trial. Lancet, 2013. 381(9863): p. 303-12.

[37]. Hong, Y.S., et al., S-1 plus oxaliplatin versus capecitabine plus oxaliplatin for first-line treatment of patients with metastatic colorectal cancer: a randomised, non-inferiority phase 3 trial. Lancet Oncol, 2012. 13(11): p. 1125-32.

[38]. Ballhausen, A., et al., Health-related quality of life in patients with RAS wild-type metastatic colorectal cancer treated with fluorouracil and folinic acid with or without panitumumab as maintenance therapy: a prespecified secondary analysis of the PanaMa (AIO KRK 0212) trial. Eur J Cancer, 2023. 190: p. 112955.
